# Computational Study of Mechanism and Thermodynamics of Ni/IPr-Catalyzed Amidation of Esters

**DOI:** 10.3390/molecules23102681

**Published:** 2018-10-18

**Authors:** Chong-Lei Ji, Pei-Pei Xie, Xin Hong

**Affiliations:** Department of Chemistry, Zhejiang University, Hangzhou 310027, China; chjicl@zju.edu.cn (C.-L.J.); xppyyfd@zju.edu.cn (P.-P.X.)

**Keywords:** amide C–N bond activation, nickel catalysis, amidation, DFT study, reaction thermodynamics

## Abstract

Nickel catalysis has shown remarkable potential in amide C–N bond activation and functionalization. Particularly for the transformation between ester and amide, nickel catalysis has realized both the forward (ester to amide) and reverse (amide to ester) reactions, allowing a powerful approach for the ester and amide synthesis. Based on density functional theory (DFT) calculations, we explored the mechanism and thermodynamics of Ni/IPr-catalyzed amidation with both aromatic and aliphatic esters. The reaction follows the general cross-coupling mechanism, involving sequential oxidative addition, proton transfer, and reductive elimination. The calculations indicated the reversible nature of amidation, which highlights the importance of reaction thermodynamics in related reaction designs. To shed light on the control of thermodynamics, we also investigated the thermodynamic free energy changes of amidation with a series of esters and amides.

## 1. Introduction

Amide C–N bond activation and functionalization provide a powerful strategy to utilize amide as a central synthon [1,2,3,4,5,6]. Despite the remarkable value of this strategy, the development of amide C–N bond activation has progressed slowly due to the resonance nature of amide and the resulting difficulty for bond dissociation [7,8,9,10,11,12,13,14,15,16]. Recently, Garg and coworkers discovered the outstanding performance of nickel catalysis for amide C–N bond activation [17], leading to the development of a series of exciting Ni-catalyzed cross coupling reactions [18,19,20,21,22,23,24,25,26]. Szostak and coworkers developed a family of geometrically twisted amides which serves as a powerful synthetic platform [27,28,29,30,31,32,33,34,35,36,37,38,39] for both metal-catalyzed and metal-free transformations. Independent studies from Shi, Zou, Rueping, Maiti, Stanley, and Molander also contribute significantly to the synthetic advances involving amide C–N bond activation [40,41,42,43,44,45,46,47,48,49,50,51,52].

**Scheme 1:**
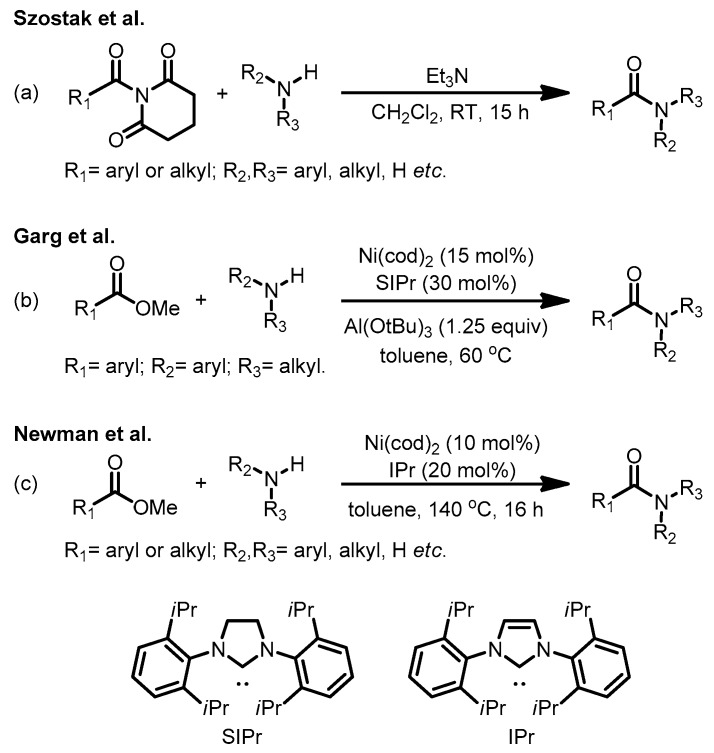
(**a**) Transamidation of *N*-acyl-glutarimides with amines; (**b**) Ni/SIPr-catalyzed amidation of ester under Lewis acid conditions; and (**c**) Ni/IPr-catalyzed amidation of ester under Lewis acid-free conditions.

In addition, amide C–N bond activation stimulated the synthetic developments of amide C–N bond formation. Szostak and coworkers reported a series of transamidation reactions from twisted amides, which provides a distinctive approach for amide synthesis [39] (Scheme 1a). Garg, Houk, and coworkers discovered that Lewis acid plays an important role on the kinetics and thermodynamics of amidation of ester, which reverses the thermodynamic equilibrium of esterification of amide [53] (Scheme 1b). Newman and coworkers recently disclosed a Ni/IPr-catalyzed amidation of ester under Lewis acid-free conditions [54] (Scheme 1c). This transformation attracts our mechanistic interests [55,56,57,58,59,60,61,62,63,64,65,66]; particularly for the Lewis-acid free Ni-mediated C–O bond cleavage and C–N bond formation. Here we report a computational study on the mechanism of Ni/IPr-catalyzed amidation of ester and the thermodynamic equilibrium of amidation.

## 2. Results and Discussion

### 2.1. Reaction Mechanism of Aromatic Ester

**Figure 1:**
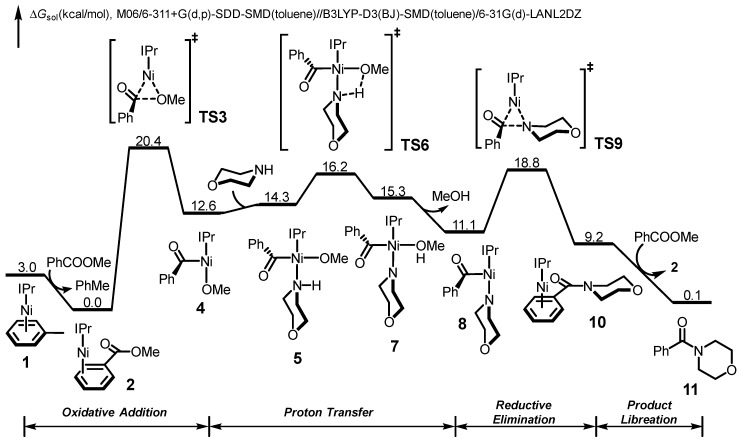
DFT-computed free energy profile of Ni/IPr-catalyzed amidation of aromatic ester.

**Figure 2:**
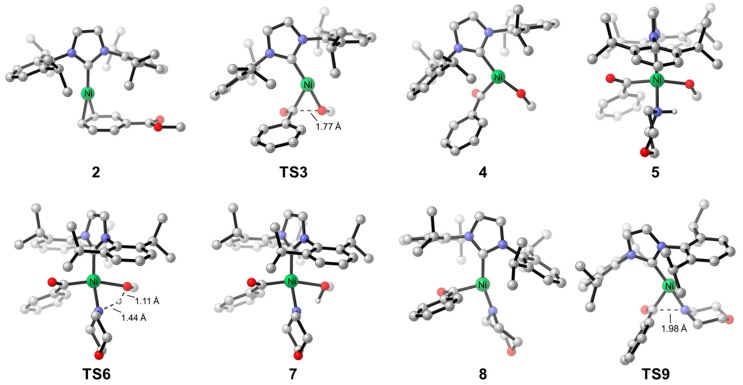
Optimized structures of selected intermediates and transition states in the catalytic cycle of Ni/IPr-catalyzed amidation of ester.

Using methyl benzoate and morpholine as the model substrates, we first explored the free energy changes of Ni/IPr-catalyzed amidation with aromatic ester. The free energy profile is shown in Figure 1, and the optimized structures of selected species in the catalytic cycle are shown in Figure 2. From the Ni(IPr)(toluene) complex **1** [67], the initial ligand exchange with substrate is exergonic and leads to the substrate-coordinated complex **2**. Subsequent oxidative addition via TS3 requires a barrier of 20.4 kcal/mol, which generates the acylnickel species **4**. **5** then undergoes a facile inner-sphere proton transfer through TS6, and the dissociation of methanol leads to the LNi^II^(acyl)(amino) intermediate **8**. From **8**, the C–N bond formation occurs via the reductive elimination transition state TS9, and the final exergonic product liberation produces the amidation product **11** as well as regenerates the catalytically active species **2**. We have confirmed the stability of wavefunctions for all Ni(II) species involved in the catalytic cycle. We were not able to locate the transition states for the ligand exchange steps (**4** to **5**, **7** to **8**) due to the flat energy surface. A series of constrained optimizations were performed to verify the flat energy surface of ligand exchange. Details are included in Supplementary Materials (Figure S1). Based on the DFT-computed free energy changes of the overall catalytic cycle, the on-cycle resting state is the substrate-coordinated complex **2**, and the rate-limiting step is the C–O bond activation step via TS3 with an overall barrier of 20.4 kcal/mol. Our computations also suggested that the overall amidation is reversible and almost thermodynamic neutral. The reaction barrier for the reversed transformation from **11** to **2** is only 20.3 kcal/mol. This corresponds well with the reversible nature of this amidation transformation, as reported by Newman [54]. It should be pointed out the generated methanol may leave the reaction system due to its volatility, which serves as an additional driving force for the amide formation.

### 2.2. Reaction Mechanism of Aliphatic Ester

**Figure 3:**
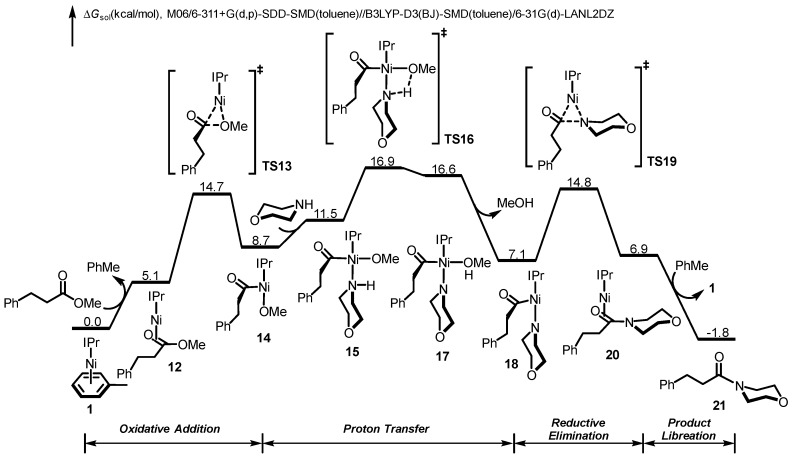
Density functional theory (DFT)-computed free energy profile of Ni/IPr-catalyzed amidation of aliphatic ester.

**Figure 4:**
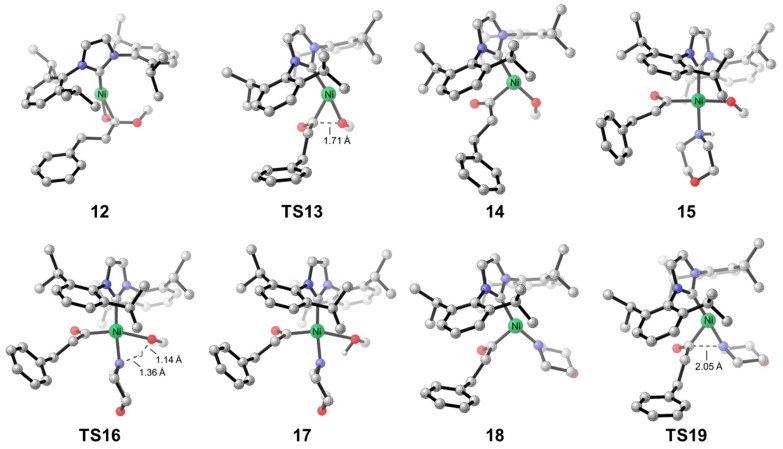
Optimized structures of selected intermediates and transition states in the catalytic cycle of Ni/IPr-catalyzed amidation of ester.

We next explored the Ni-catalyzed amidation with aliphatic ester, using methyl 3-phenylpropionate as the model substrate. The DFT-computed free energy profile is shown in Figure 3, and the optimized structures of selected species in the catalytic cycle are shown in Figure 4. The reaction mechanism of aliphatic ester is similar to that of aromatic ester, which involves sequential oxidative addition, proton transfer, reductive elimination and product liberation. The resting state is toluene-coordinated complex **1**, and the rate-limiting step is the proton transfer step with an overall barrier of 16.9 kcal/mol. The free energy change of the amidation with methyl 3-phenylpropionate is exergonic by 1.8 kcal/mol. Comparing the free energy profiles of aromatic and aliphatic esters, the major difference is the resting state and overall reaction barrier. Due to the lack of electron-deficient aromatic functionality in methyl 3-phenylpropionate, the substrate-coordinated complex **12** is 6.1 kcal/mol higher in free energy comparing with the toluene-coordinated species **1**. This leads to the low overall barrier of the amidation with aliphatic ester.

### 2.3. Reaction Thermodynamics

**Figure 5:**
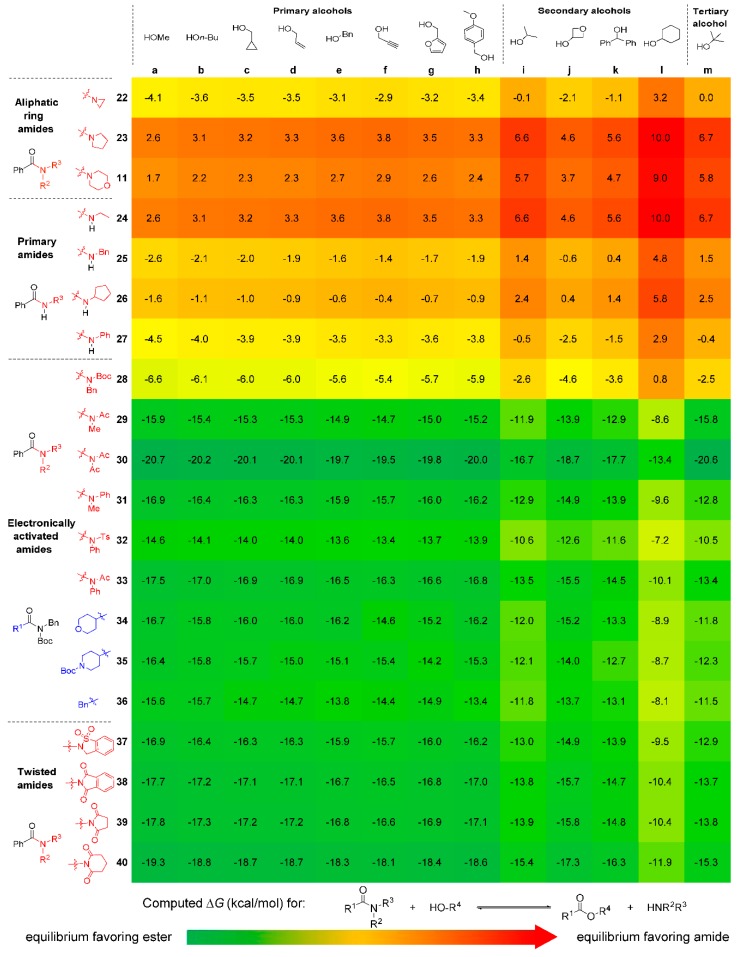
DFT-computed thermodynamics of esterification of amides.

Based on the computed free energy profiles of Ni-catalyzed amidation of ester, we noticed that the major bottleneck for this type of transformation is the control of thermodynamics. The thermodynamic equilibrium has to be designed cautiously because the overall transformation does not have a strong force, unlike many other organic transformations. Therefore, the synthetic efforts could be unfruitful even the designed amidation or esterification has a surmountable reaction barrier. In order to understand the controlling factors of the thermodynamic equilibrium of amidation as well as provide a general thermodynamic guideline for related reaction designs, we computed the free energy changes with a series of esters and amides. The results are summarized as a heat map in Figure 5. Green represents the thermodynamic equilibrium that favors the formation of ester, and red indicates the opposite trend.

For the explored esters and amides, the alcohol component (derivatization of ester) have limited effects on the reaction thermodynamics. The effects from the change of primary alcohols are less than 1 kcal/mol for most amides (**a** to **h**). Comparing with the primary alcohols, the esters with secondary alcohols are less stable (**i** to **l**), especially for cyclohexanol. The thermodynamic equilibrium involving cyclohexanol favors the amide formation the most among all the studied alcohols. This suggest that the cyclohexanol derived esters can be useful substrates for amide synthesis. Interestingly, the tertiary alcohol is not the best alcohol for amide formation, potentially due to the steric repulsions in the corresponding esters.

Unlike the alcohols, the structure of amides has profound effects on the thermodynamic equilibrium of amidation. Without strong electron-activating groups or geometric twisting, amides are more stable than the corresponding esters regardless of the effects from alcohol (**23** and **24**). This follows the consensus that amide is generally more stable than ester due to the strong resonance nature. However, the electronically activated amides (**28**–**36**) and twisted amides (**37**–**40**) are significantly less stable in terms of thermodynamics. The equilibrium involving these amides all favor the ester formation, providing significant driving force for amide C–N bond activation and functionalization. Based on the computed thermodynamics, we found that the *N*-substitution has important effect on the thermodynamic stability of amides. This corroborates Szostak’s design of twisted amides [27,28,29,30,31,32,33,34,35,36,37,38,39], and also highlights the importance of amide design for future synthetic transformations involving amides.

## 3. Computational Methods

All density functional theory (DFT) calculations were carried out using Gaussian16 software package [68]. Geometry optimizations and frequency calculations were conducted using B3LYP [69,70] functional with Grimme’s D3(BJ) empirical dispersion correction [71], the LANL2DZ [72] basis set for nickel, and 6-31G(d) basis set for all the other elements. The solvation energy corrections were also included in the geometry optimizations and frequency calculations. Based on the solution phase-optimized geometries, single-point energies were calculated with the M06 [73] functional, the SDD [74,75] basis set for nickel, and 6-311G++(d,p) basis set for all other elements. Solvation energies were calculated using the SMD solvation model [76] with toluene as the solvent. No frequency scaling factor was used to calculate Gibbs energy corrections. The 3D diagrams of computed species were visualized using CYLview [77].

## 4. Conclusions

In summary, the mechanism and thermodynamics of Ni/IPr-catalyzed amidation of esters have been explored with DFT calculations. The reaction proceeds via the general cross-coupling mechanism. Nickel catalyst first cleaves the C–O bond of ester via oxidative addition, and subsequent proton transfer between LNi(acyl)(OR) species and amine leads to LNi(acyl)(amino) intermediate. This intermediate undergoes C–N reductive elimination to produce the amidation product. The studied aromatic and aliphatic esters both have fairly low reaction barriers, 20.4 kcal/mol for methyl benzoate and 16.9 kcal/mol for methyl 3-phenylpropionate. However, the overall amidation with morpholine is almost thermodynamic neutral. This suggests that the amidation only has limited thermodynamic driving force, and the evaporation of methanol could be critical for productivity. Therefore, the key to reaction success of Ni/NHC-catalyzed amidation of ester is the control of thermodynamic equilibrium.

For the thermodynamic free energy changes of the esterification of amide, the O-substitution of ester has limited effects, and cyclohexanol derivative is the most stable ester among all the studied cases. In contrast to the O-substitution of ester, the N-substitution of amide has profound effects on the thermodynamic stability of amides. Electron-activating groups and geometric twisting can significantly destabilize amides and provide the desired driving force towards the amide C–N bond activation. These computations highlight the design of amide for future reaction development involving amide C–N bond activation.

## Supplementary Materials

Detailed energies and coordinates of computed saddle points are available online.

## References

[1] Meng G., Shi S., Szostak M. (2016). Cross-Coupling of Amides by N–C Bond Activation. Synlett.

[2] Meng G., Szostak M. (2016). Palladium-Catalyzed Suzuki–Miyaura Coupling of Amides by Carbon–Nitrogen Cleavage: General Strategy for Amide N–C Bond Activation. Org. Biomol. Chem..

[3] Liu C., Szostak M. (2017). Twisted Amides: From Obscurity to Broadly Useful Transition-Metal-Catalyzed Reactions by N–C Amide Bond Activation. Chem. Eur. J..

[4] Dander J.E., Garg N.K. (2017). Breaking Amides using Nickel Catalysis. ACS Catal..

[5] Takise R., Muto K., Yamaguchi J. (2017). Cross-coupling of aromatic esters and amides. Chem. Soc. Rev..

[6] Gao Y., Ji C.-L., Hong X. (2017). Ni-mediated C–N activation of amides and derived catalytic transformations. Sci. China Chem..

[7] Wiberg K.B., Breneman C.M. (1992). Resonance interactions in acyclic systems. 3. Formamide internal rotation revisited. Charge and energy redistribution along the C–N bond rotational pathway. J. Am. Chem. Soc..

[8] Wiberg K.B. (1999). The Interaction of Carbonyl Groups with Substituents. Acc. Chem. Res..

[9] Kemnitz C.R., Loewen M.J. (2007). “Amide Resonance” Correlates with a Breadth of C–N Rotation Barriers. J. Am. Chem. Soc..

[10] Glover S.A., Rosser A.A. (2012). Reliable Determination of Amidicity in Acyclic Amides and Lactams. J. Org. Chem..

[11] Szostak R., Aubé J., Szostak M. (2015). Determination of Structures and Energetics of Small- and Medium-Sized One-Carbon Bridged Twisted Amides using ab Initio Molecular Orbital Methods. Implications for Amidic Resonance along the C–N Rotational Pathway. J. Org. Chem..

[12] Hu F., Lalancette R., Szostak M. (2016). Structural Characterization of *N*–Alkylated Twisted Amides: Consequences for Amide Bond Resonance and N–C Cleavage. Angew. Chem. Int. Ed..

[13] Szostak R., Meng G., Szostak M. (2017). Resonance Destabilization in *N*-Acylanilines (Anilides): Electronically-Activated Planar Amides of Relevance in N–C(O) Cross-Coupling. J. Org. Chem..

[14] Szostak R., Szostak M. (2018). *N*-Acyl-Glutarimides: Resonance and Proton Affinities of Rotationally-Inverted Twisted Amides Relevant to N–C(O) Cross-Coupling. Org. Lett..

[15] Bisz E., Piontek A., Dziuk B., Szostak R., Szostak M. (2018). Barriers to Rotation in ortho-Substituted Tertiary Aromatic Amides: Effect of Chloro-Substitution on Resonance and Distortion. J. Org. Chem..

[16] Meng G., Shi S., Lalancette R., Szostak R., Szostak M. (2018). Reversible Twisting of Primary Amides via Ground State N–C(O) Destabilization: Highly Twisted Rotationally Inverted Acyclic Amides. J. Am. Chem. Soc..

[17] Hie L., Nathel N.F.F., Shah T.K., Baker E.L., Hong X., Yang Y.-F., Liu P., Houk K.N., Garg N.K. (2015). Conversion of amides to esters by the nickel-catalysed activation of amide C–N bonds. Nature.

[18] Weires N.A., Baker E.L., Garg N.K. (2016). Nickel-Catalysed Suzuki-Miyaura Coupling of Amides. Nat. Chem..

[19] Simmons B.J., Weires N.A., Dander J.E., Garg N.K. (2016). Nickel-Catalyzed Alkylation of Amide Derivatives. ACS Catal..

[20] Baker E.L., Yamano M.M., Zhou Y., Anthony S.M., Garg N.K. (2016). A Two-Step Approach to Achieve Secondary Amide Transamidation Enabled by Nickel Catalysis. Nat. Commun..

[21] Dander J.E., Weires N.A., Garg N.K. (2016). Benchtop Delivery of Ni(cod)_2_ using Paraffin Capsules. Org. Lett..

[22] Hie L., Baker E.L., Anthony S.M., Desrosier J., Senanayake C., Garg N.K. (2016). Nickel-Catalyzed Esterification of Aliphatic Amides. Angew. Chem. Int. Ed..

[23] Medina J.M., Moreno J., Racine S., Du S., Garg N.K. (2017). Mizoroki–Heck Cyclizations of Amide Derivatives for the Introduction of Quaternary Centers. Angew. Chem. Int. Ed..

[24] Weires N.A., Caspi D.D., Garg N.K. (2017). Kinetic Modeling of the Nickel-Catalyzed Esterification of Amides. ACS Catal..

[25] Dander J.E., Baker E.L., Garg N.K. (2017). Nickel-Catalyzed Transamidation of Aliphatic Amide Derivatives. Chem. Sci..

[26] Boit T.B., Weires N.A., Kim J., Garg N.K. (2018). Nickel-Catalyzed Suzuki–Miyaura Coupling of Aliphatic Amides. ACS Catal..

[27] Meng G., Szostak M. (2015). Sterically-Controlled Pd-Catalyzed Chemoselective Ketone Synthesis via N–C Cleavage in Twisted Amides. Org. Lett..

[28] Meng G., Szostak M. (2015). General Olefin Synthesis by the Palladium-Catalyzed Heck Reaction of Amides: Sterically-Controlled Chemoselective N–C Activation. Angew. Chem. Int. Ed..

[29] Meng G., Szostak M. (2016). Rhodium-Catalyzed C–H Bond Functionalization with Amides by Double C–H/C–N Bond Activation. Org. Lett..

[30] Shi S., Meng G., Szostak M. (2016). Synthesis of Biaryls via Nickel Catalyzed Suzuki–Miyaura Coupling of Amides by Carbon–Nitrogen Cleavage. Angew. Chem. Int. Ed..

[31] Liu Y., Meng G., Liu R., Szostak M. (2016). Sterically-Controlled Intermolecular Friedel-Crafts Acylation with Twisted Amides via Selective N–C Cleavage under Mild Conditions. Chem. Commun..

[32] Shi S., Szostak M. (2016). Efficient Synthesis of Diaryl Ketones by Nickel-Catalyzed Negishi Cross-Coupling of Amides via Carbon–Nitrogen Bond Cleavage at Room Temperature Accelerated by Solvent Effect. Chem. Eur. J..

[33] Lei P., Meng G., Szostak M. (2017). General Method for the Suzuki–Miyaura Cross-Coupling of Amides Using Commercially Available, Air- and Moisture-Stable Palladium/NHC (NHC = N-Heterocyclic Carbene) Complexes. ACS Catal..

[34] Shi S., Szostak M. (2017). Decarbonylative Cyanation of Amides by Palladium Catalysis. Org. Lett..

[35] Shi S., Szostak M. (2017). Nickel-Catalyzed Negishi Cross-Coupling of N-Acylsuccinimides: Stable, Amide-Based, Twist-Controlled Acyl-Transfer Reagents via N–C Activation. Synthesis.

[36] Lei P., Meng G., Ling Y., An J., Szostak M. (2017). Pd-PEPPSI: Pd-NHC Precatalyst for Suzuki−Miyaura Cross-Coupling Reactions of Amides. J. Org. Chem..

[37] Liu C., Szostak M. (2017). Decarbonylative Phosphorylation of Amides by Palladium and Nickel Catalysis: The Hirao Cross-Coupling of Amide Derivatives. Angew. Chem. Int. Ed..

[38] Osumi Y., Liu C., Szostak M. (2017). *N*-Acylsuccinimides: Twist-Controlled, Acyl-Transfer Reagents in Suzuki–Miyaura Cross-Coupling by N–C Amide Bond Activation. Org. Biomol. Chem..

[39] Liu Y., Achtenhagen M., Liu R., Szostak M. (2018). Transamidation of *N*-Acyl-Glutarimides with Amines. Org. Biomol. Chem..

[40] Hu J., Zhao Y., Liu J., Zhang Y., Shi Z. (2016). Nickel-Catalyzed Decarbonylative Borylation of Amides: Evidence for Acyl C-N Bond Activation. Angew. Chem. Int. Ed..

[41] Hu J., Wang M., Pu X., Shi Z. (2017). Nickel-Catalyzed Retro-Hydroamidocarbonylation of Aliphatic Amides to Olefins. Nat. Commun..

[42] Li X., Zou G. (2015). Acylative Suzuki coupling of amides: Acyl-nitrogen activation via synergy of independently modifiable activating groups. Chem. Commun..

[43] Yue H., Guo L., Lee S., Liu X., Rueping M. (2017). Selective Reductive Removal of Ester and Amide Groups from Arenes and Heteroarenes through Nickel–Catalyzed C–O and C–N Bond Activation. Angew. Chem. Int. Ed..

[44] Srimontree W., Chatupheeraphat A., Liao H., Rueping M. (2017). Amide to Alkyne Interconversion via a Nickel/Copper-Catalyzed Deamidative Cross-Coupling of Aryl and Alkenyl Amides. Org. Lett..

[45] Chatupheeraphat A., Liao H., Lee S., Rueping M. (2017). Nickel-Catalyzed C–CN Bond Formation via Decarbonylative Cyanation of Esters, Amides, and Intramolecular Recombination Fragment Coupling of Acyl Cyanides. Org. Lett..

[46] Liu X., Yue H., Jia J., Guo L., Rueping M. (2017). Synthesis of Amidines from Amides using a Nickel Catalyzed Decarbonylative Amination via CO Extrusion Intramolecular Recombination Fragment Coupling. Chem. Eur. J..

[47] Lee S., Guo L., Yue H., Liao H., Rueping M. (2017). Nickel-Catalyzed Decarbonylative Silylation, Borylation, and Amination of Arylamides via a Deamidative Reaction Pathway. Synlett.

[48] Lee S., Liao H., Chatupheeraphat A., Rueping M. (2018). Nickel-Catalyzed C–S Bond Formation via Decarbonylative Thioetherification of Esters, Amides and Intramolecular Recombination Fragment Coupling of Thioesters. Chem. Eur. J..

[49] Liu X., Hsiao C., Guo L., Rueping M. (2018). Cross-Coupling of Amides with Alkylboranes via Nickel-Catalyzed C–N Bond Cleavage. Org. Lett..

[50] Dey A., Sasmal S., Seth K., Lahiri G.K., Maiti D. (2017). Nickel-Catalyzed Deamidative Step-Down Reduction of Amides to Aromatic Hydrocarbons. ACS Catal..

[51] Walker J.A., Vickerman K.L., Humke J.N., Stanley L.M. (2017). Ni-Catalyzed Alkene Carboacylation via Amide C–N Bond Activation. J. Am. Chem. Soc..

[52] Amani J., Alam R., Badir S., Molander G.A. (2017). Synergistic Visible-Light Photoredox/Nickel-Catalyzed Synthesis of Aliphatic Ketones via N–C Cleavage of Imides. Org. Lett..

[53] Hie L., Nathel N.F.F., Hong X., Yang Y.-F., Houk K.N., Garg N.K. (2016). Nickel-Catalyzed Activation of Acyl C–O Bonds of Methyl Esters. Angew. Chem. Int. Ed..

[54] Halima T.B., Masson-Makdissi J., Newman S.G. (2018). Nickel-Catalyzed Amide Bond Formation from Methyl Esters. Angew. Chem. Int. Ed..

[55] Li Z., Zhang S., Fu Y., Guo Q., Liu L. (2009). Mechanism of Ni-Catalyzed Selective C–O Bond Activation in Cross-Coupling of Aryl Esters. J. Am. Chem. Soc..

[56] Yu H.-Z., Fu Y. (2012). Mechanistic Origin of Cross-Coupling Selectivity in Ni-Catalysed Tishchenko Reactions. Chem. Eur. J..

[57] Hong X., Liang Y., Houk K.N. (2014). Mechanisms and Origins of Switchable Chemoselectivity of Ni-Catalyzed C(aryl)–O and C(acyl)–O Activation of Aryl Esters with Phosphine Ligands. J. Am. Chem. Soc..

[58] Lu Q., Yu H.-Z., Fu Y. (2014). Mechanistic Study of Chemoselectivity in Ni-Catalyzed Coupling Reactions between Azoles and Aryl Carboxylates. J. Am. Chem. Soc..

[59] Xu H., Muto K., Yamaguchi J., Zhao C., Itami K., Musaev D.G. (2014). Key Mechanistic Features of Ni-Catalyzed C–H/C–O Biaryl Coupling of Azoles and Naphthalen-2-yl Pivalates. J. Am. Chem. Soc..

[60] Muto K., Yamaguchi J., Musaev D.G., Itami K. (2015). Decarbonylative organoboron cross-coupling of esters by nickel catalysis. Nat. Commun..

[61] Li Z., Liu L. (2015). Recent advances in mechanistic studies on Ni catalyzed cross-coupling reactions. Chin. J. Catal..

[62] Liu L., Chen P., Sun Y., Wu Y., Chen S., Zhu J., Zhao Y. (2016). Mechanism of Nickel-Catalyzed Selective C–N Bond Activation in Suzuki–Miyaura Cross-Coupling of Amides: A Theoretical Investigation. J. Org. Chem..

[63] Xu Z.-Y., Yu H.-Z., Fu Y. (2017). Mechanism of Nickel-Catalyzed Suzuki–Miyaura Coupling of Amides. Chem. Asian J..

[64] Ji C.-L., Hong X. (2017). Factors Controlling the Reactivity and Chemoselectivity of Resonance Destabilized Amides in Ni-Catalyzed Decarbonylative and Nondecarbonylative Suzuki–Miyaura Coupling. J. Am. Chem. Soc..

[65] Jiang Y.-Y., Liu T., Sun X., Xu Z., Fan X., Zhu L., Bi S.-W. (2018). Computational study of the mechanism of amide bond formation via CS2-releasing 1,3-acyl transfer. Org. Biomol. Chem..

[66] Li Y., Zou L., Bai R., Lan Y. (2018). Ni(I)–Ni(III) vs. Ni(II)–Ni(IV): Mechanistic study of Ni-catalyzed alkylation of benzamides with alkyl halides. Org. Chem. Front..

[67] Saper N.I., Hartwig J.F. (2017). Mechanistic Investigations of the Hydrogenolysis of Diaryl Ethers Catalyzed by Nickel Complexes of N-Heterocyclic Carbene Ligands. J. Am. Chem. Soc..

[68] Frisch M.J., Trucks G.W., Schlegel H.B., Scuseria G.E., Robb M.A., Cheeseman J.R., Scalmani G., Barone V., Petersson G.A., Nakatsuji H. (2016). Gaussian 16.

[69] Head-Gordon M., Pople J.A., Frisch M. (1988). MP2 energy evaluation by direct methods. Chem. Phys. Lett..

[70] Lee C., Yang W., Parr R.G. (1988). Development of the Colle-Salvetti correlation-energy formula into a functional of the electron density. Phys. Rev. B Condens. Matter Mater. Phys..

[71] Grimme S., Antony J., Ehrlich S., Krieg H. (2010). A consistent and accurate ab initio parametrization of density functional dispersion correction (DFT-D) for the 94 elements H-Pu. J. Chem. Phys..

[72] Hay P.J., Wadt W.R. (1985). Ab initio effective core potentials for molecular calculations. Potentials for K to Au including the outermost core orbitals. J. Chem. Phys..

[73] Zhao Y., Truhlar D.G. (2008). The M06 suite of density functionals for main group thermochemistry, thermochemical kinetics, noncovalent interactions, excited states, and transition elements: Two new functionals and systematic testing of four M06-class functionals and 12 other functionals. Theor. Chem. Acc..

[74] Häussermann U., Dolg M., Stoll H., Preuss H., Schwerdtfeger P., Pitzer R.M. (1993). Accuracy of energy-adjusted quasirelativistic ab initio pseudopotentials. Mol. Phys..

[75] Küchle W., Dolg M., Stoll H., Preuss H. (1994). Energy-adjusted pseudopotentials for the actinides. Parameter sets and test calculations for thorium and thorium monoxide. J. Chem. Phys..

[76] Marenich A.V., Cramer C.J., Truhlar D.G. (2009). Universal Solvation Model Based on Solute Electron Density and on a Continuum Model of the Solvent Defined by the Bulk Dielectric Constant and Atomic Surface Tensions. J. Phys. Chem. B.

[77] (2009). CYLview, 1.0b.

