# Supplementary material for: Computational Study of Mechanism and Thermodynamics of Ni/IPr-Catalyzed Amidation of Esters
Source: Molecules. 2018 Oct 18;23(10):2681. doi: 10.3390/molecules23102681 (PMC6222384; doi:10.3390/molecules23102681)
Supplement: Supplementary file 1 [file molecules-23-02681-s001.pdf]

Supplemental Material

Computational study of mechanism and thermodynamics of Ni/IPr-catalyzed amidation of esters

Chong-Lei Ji,† Pei-Pei Xie,† Xin Hong*

Department of Chemistry, Zhejiang University, Hangzhou, 310027, China.

chjicl@zju.edu.cn (C. -L. J.); xppyyfd@zju.edu.cn (P. -P.; X.)

* Correspondence: 0016011@zju.edu.cn (X. H.); Tel.: +86-571-88273191

† These authors contributed equally to this work.

Contents

1. Energy scan for the ligand exchange steps in Ni/IPr-catalyzed amidation of aromatic ester. ………………………………………………………………..…………S2
2. Tables of energies. ……………………………………………………………………S2
3. Cartesian coordinates of the structures. ………………………………………………S9

Energy scan for the ligand exchange steps in Ni/IPr-catalyzed amidation of aromatic ester.

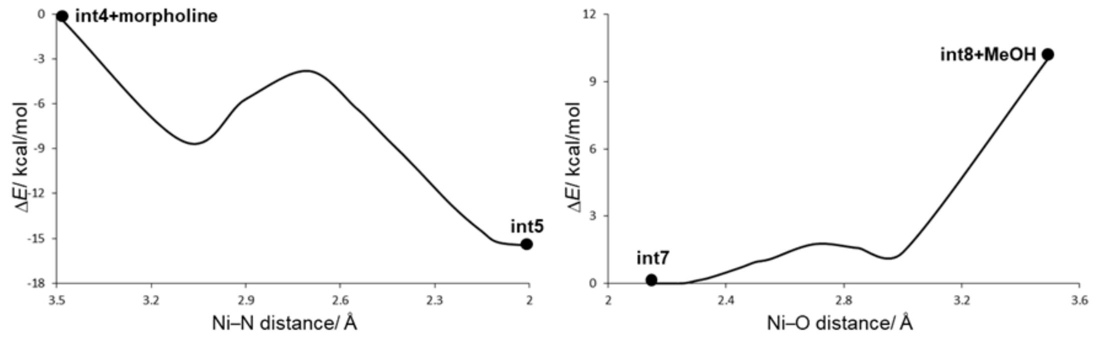

Figure S1. Energy scan for the ligand exchange steps (a) **4** to **5** (b) **7** to **8**.

We were not able to locate the ligand exchange transition states due to the flat energy surface. A series of constrained optimizations were performed to explore the energy surface of ligand exchange processes, whose results corroborate the flat energy surface of ligand exchange. Therefore, the ligand exchange steps are not rate-determining for the catalytic cycle.

Table of energies

Zero-point correction (*ZPE*), thermal correction to enthalpy (*TCH*), thermal correction to Gibbs free energy (*TCG*), energies (*E*), enthalpies (*H*), and Gibbs free energies (*G*) (in Hartree) of the structures calculated at the M06/6-311+G(d,p)-SDD-SMD(toluene)//B3LYP-D3(BJ)-SMD(toluene)/6-31G(d)-LANL2DZ level of theory.

Table S1. Energies in Figure 1, Figure 3, Figure 5

Structures	*ZPE*	*tcH*	*tcG*	*E*	*H*	*G*	Imaginary Frequency
1	0.703789	0.744124	0.630345	-1601.888877	-1601.144753	-1601.258532	
2	0.719262	0.762449	0.642065	-1790.423540	-1789.661091	-1789.781475	
TS3	0.718634	0.760789	0.644939	-1790.393962	-1789.633173	-1789.749023	253.9i
4	0.718753	0.761934	0.643570	-1790.404970	-1789.643036	-1789.761400	
5	0.859096	0.907887	0.778662	-2078.114782	-2077.206895	-2077.336120	
TS6	0.853820	0.902451	0.773990	-2078.107079	-2077.204628	-2077.333089	333.5i
7	0.855201	0.904713	0.773218	-2078.107729	-2077.203016	-2077.334511	
8	0.801590	0.847692	0.721619	-1962.400694	-1961.553002	-1961.679075	
TS9	0.801073	0.846390	0.722552	-1962.389309	-1961.542919	-1961.666757	187.1i
10	0.804009	0.849288	0.726642	-1962.408755	-1961.559467	-1961.682113	
11	0.227419	0.240017	0.188542	-631.941423	-631.701406	-631.752881	
12	0.778309	0.822935	0.701585	-1869.002843	-1868.179908	-1868.301258	
TS13	0.775525	0.820736	0.695454	-1868.981477	-1868.160741	-1868.286023	190.9i
14	0.775505	0.821642	0.694221	-1868.989665	-1868.168023	-1868.295444	
15	0.916319	0.967822	0.831251	-2156.699659	-2155.731837	-2155.868408	
TS16	0.910584	0.962250	0.824710	-2156.684619	-2155.722369	-2155.859909	587.3i
17	0.913290	0.965641	0.826054	-2156.686295	-2155.720654	-2155.860241	

18	0.858166	0.907222	0.772672	-2040.986045	-2040.078823	-2040.213373	
TS19	0.858436	0.906402	0.776103	-2040.977149	-2040.070747	-2040.201046	160.8i
20	0.860915	0.908837	0.777810	-2040.991396	-2040.082559	-2040.213586	
21	0.284678	0.300032	0.240004	-710.528489	-710.228457	-710.288485	
22	0.162149	0.172219	0.127052	-478.116863	-477.944644	-477.989811	
23	0.222158	0.234089	0.184482	-556.741560	-556.507471	-556.557078	
24	0.185336	0.196645	0.148253	-479.371013	-479.174368	-479.174368	
25	0.238793	0.252913	0.196520	-670.999873	-670.74696	-670.803353	
26	0.250644	0.264128	0.264128	-596.035368	-595.77124	-595.77124	
27	0.210073	0.222843	0.170505	-631.716051	-631.493208	-631.545546	
28	0.365631	0.388142	0.312856	-1016.703675	-1016.315533	-1016.390819	
29	0.194689	0.207702	0.155818	-592.676185	-592.468483	-592.520367	
30	0.203046	0.218159	0.160764	-705.988867	-705.770708	-705.828103	
31	0.237357	0.251839	0.195892	-670.993371	-670.741532	-670.797479	
32	0.327149	0.350027	0.272549	-1450.472304	-1450.122277	-1450.199755	
33	0.246217	0.262602	0.201615	-784.305515	-784.042913	-784.103900	
34	0.412550	0.435972	0.359238	-1056.23664	-1055.800668	-1055.877402	
35	0.552991	0.584995	0.486896	-1382.091141	-1381.506146	-1381.604245	
36	0.393956	0.417893	0.337180	-1055.995283	-1055.57739	-1055.658103	
37	0.207120	0.223976	0.162557	-1292.358959	-1292.134983	-1292.196402	
38	0.207121	0.222420	0.164342	-857.139574	-856.917154	-856.975232	
39	0.182902	0.196290	0.142096	-704.79575	-704.59946	-704.653654	
40	0.212479	0.226781	0.171000	-744.087475	-743.860694	-743.916475	
a	0.051406	0.055647	0.028679	-115.69086	-115.635213	-115.662181	
b	0.137677	0.145457	0.107544	-233.571694	-233.426237	-233.46415	
c	0.114959	0.121556	0.086356	-232.33765	-232.216094	-232.251294	
d	0.084984	0.090991	0.057711	-193.044168	-192.953177	-192.986457	
e	0.133394	0.141493	0.101287	-346.622518	-346.481025	-346.521231	
f	0.060800	0.066582	0.034141	-191.795309	-191.728727	-191.761168	
g	0.102867	0.110308	0.071807	-344.434472	-344.324164	-344.362665	
h	0.166403	0.177005	0.130763	-461.115628	-460.938623	-460.984865	
i	0.108496	0.114853	0.081128	-194.286212	-194.171359	-194.205084	
j	0.091537	0.097474	0.063585	-268.246448	-268.148974	-268.182863	
k	0.214890	0.227311	0.175615	-577.555081	-577.32777	-577.379466	
l	0.175555	0.183301	0.145267	-310.964466	-310.781165	-310.819199	
m	0.136305	0.143928	0.107356	-233.583873	-233.439945	-233.476517	
PhCOOMe	0.144266	0.154012	0.109810	-459.947633	-459.793621	-459.837823	
PhMe	0.128400	0.135613	0.095906	-271.415508	-271.279895	-271.319602	
Morpholine	0.136143	0.142356	0.107710	-287.685128	-287.542772	-287.577418	
MeOH	0.051397	0.055637	0.028670	-115.690860	-115.635223	-115.662190	
Ester product of rxn between 22 and a	0.144339	0.154048	0.109981	-459.947635	-459.793587	-459.837654	
Ester product	0.230012	0.243800	0.188839	-577.827637	-577.583837	-577.638798	

of rxn between 22 and b						
Ester product of rxn between 22 and c	0.207401	0.219950	0.167927	-576.593805	-576.373855	-576.425878
Ester product of rxn between 22 and d	0.177469	0.189359	0.138890	-537.299785	-537.110426	-537.160895
Ester product of rxn between 22 and e	0.225742	0.239860	0.182658	-690.877722	-690.637862	-690.695064
Ester product of rxn between 22 and f	0.153257	0.164861	0.115457	-536.050193	-535.885332	-535.934736
Ester product of rxn between 22 and g	0.195425	0.208733	0.153809	-688.690571	-688.481838	-688.536762
Ester product of rxn between 22 and h	0.258777	0.275409	0.212657	-805.371889	-805.09648	-805.159232
Ester product of rxn between 22 and i	0.200985	0.213284	0.163052	-538.537288	-538.324004	-538.374236
Ester product of rxn between 22 and j	0.184015	0.195979	0.145345	-612.50048	-612.304501	-612.355135
Ester product of rxn between 22 and k	0.306809	0.325514	0.256863	-921.807084	-921.48157	-921.550221
Ester product of rxn between 22 and l	0.267598	0.281829	0.225775	-655.208738	-654.926909	-654.982963
Ester product of rxn between 22 and m	0.228608	0.242260	0.189398	-577.939103	-577.696843	-577.749705
Ester product of rxn between 34 and a	0.190679	0.201511	0.154590	-499.478449	-499.276938	-499.323859
Ester product of rxn between 34 and b	0.276570	0.291452	0.233537	-617.35792	-617.066468	-617.124383
Ester product of rxn between 34 and c	0.253931	0.267598	0.212198	-616.124014	-615.856416	-615.911816

Ester product of rxn between 34 and d	0.223917	0.236908	0.183646	-576.830727	-576.593819	-576.647081
Ester product of rxn between 34 and e	0.272132	0.287371	0.226588	-730.408696	-730.121325	-730.182108
Ester product of rxn between 34 and f	0.199954	0.212572	0.161100	-575.580572	-575.368000	-575.419472
Ester product of rxn between 34 and g	0.241935	0.256339	0.198799	-728.220855	-727.964516	-728.022056
Ester product of rxn between 34 and h	0.304996	0.322788	0.256848	-844.902572	-844.579784	-844.645724
Ester product of rxn between 34 and i	0.247656	0.261003	0.208341	-578.067591	-577.806588	-577.85925
Ester product of rxn between 34 and j	0.230246	0.243408	0.189052	-652.031288	-651.78788	-651.842236
Ester product of rxn between 34 and k	0.353321	0.373131	0.301614	-961.33734	-960.964209	-961.035726
Ester product of rxn between 34 and l	0.314062	0.329345	0.271033	-694.739563	-694.410218	-694.46853
Ester product of rxn between 34 and m	0.275189	0.289930	0.234539	-617.364993	-617.075063	-617.130454
Ester product of rxn between 35 and a	0.331327	0.350587	0.283064	-825.333391	-824.982804	-825.050327
Ester product of rxn between 35 and b	0.416971	0.440323	0.362179	-943.213511	-942.773188	-942.851332
Ester product of rxn between 35 and c	0.394266	0.416404	0.341053	-941.979364	-941.56296	-941.638311
Ester product of rxn between 35 and d	0.364393	0.385797	0.312948	-902.685314	-902.299517	-902.372366
Ester product of rxn between	0.412810	0.436448	0.356513	-1056.263697	-1055.827249	-1055.907184

35 and e						
Ester product of rxn between 35 and f	0.340162	0.361327	0.288623	-901.436199	-901.074872	-901.147576
Ester product of rxn between 35 and g	0.382207	0.405144	0.327082	-1054.074361	-1053.669217	-1053.747279
Ester product of rxn between 35 and h	0.445652	0.471900	0.385989	-1170.757233	-1170.285333	-1170.371244
Ester product of rxn between 35 and i	0.387944	0.409796	0.336557	-903.922874	-903.513078	-903.586317
Ester product of rxn between 35 and j	0.370935	0.392449	0.318753	-977.885931	-977.493482	-977.567178
Ester product of rxn between 35 and k	0.493925	0.522170	0.430743	-1287.192365	-1286.670195	-1286.761622
Ester product of rxn between 35 and l	0.454526	0.478306	0.399277	-1020.594246	-1020.11594	-1020.194969
Ester product of rxn between 35 and m	0.415560	0.438774	0.362617	-943.22071	-942.781936	-942.858093
Ester product of rxn between 36 and a	0.172779	0.183950	0.134274	-499.237142	-499.053192	-499.102868
Ester product of rxn between 36 and b	0.258476	0.273723	0.212272	-617.117253	-616.84353	-616.904981
Ester product of rxn between 36 and c	0.235840	0.249816	0.192946	-615.883466	-615.63365	-615.69052
Ester product of rxn between 36 and d	0.205964	0.219271	0.163938	-576.5897	-576.370429	-576.425762
Ester product of rxn between 36 and e	0.254688	0.269898	0.210065	-730.169014	-729.899116	-729.958949
Ester product of rxn between 36 and f	0.181728	0.194762	0.140275	-575.340167	-575.145405	-575.199892
Ester product	0.223864	0.238615	0.177979	-727.980222	-727.741607	-727.802243

of rxn between 36 and g						
Ester product of rxn between 36 and h	0.287781	0.305367	0.241718	-844.663679	-844.358312	-844.421961
Ester product of rxn between 36 and i	0.229463	0.243168	0.187873	-577.827566	-577.584398	-577.639693
Ester product of rxn between 36 and j	0.212374	0.225827	0.169671	-651.790247	-651.56442	-651.620576
Ester product of rxn between 36 and k	0.335418	0.355393	0.283773	-961.099938	-960.744545	-960.816165
Ester product of rxn between 36 and l	0.296201	0.311763	0.251178	-694.499069	-694.187306	-694.247891
Ester product of rxn between 36 and m	0.257085	0.272142	0.214630	-617.125326	-616.853184	-616.910696
Amine product of rxn between 22 and a	0.070614	0.074784	0.046447	-133.867327	-133.792543	-133.82088
Amine product of rxn between 23 and a	0.130537	0.136455	0.101997	-212.479452	-212.342997	-212.377455
Amine product of rxn between 11 and a	0.135653	0.142257	0.106496	-287.674602	-287.532345	-287.568106
Amine product of rxn between 24 and a	0.093301	0.098606	0.067839	-135.110938	-135.012332	-135.043099
Amine product of rxn between 25 and a	0.146830	0.154931	0.114908	-326.747009	-326.592078	-326.632101
Amine product of rxn between 26 and a	0.158798	0.166086	0.128496	-251.781497	-251.615411	-251.653001
Amine product of rxn between 27 and a	0.117520	0.124220	0.088396	-287.465663	-287.341443	-287.377267
Amine product of rxn between 28 and a	0.274926	0.291062	0.230709	-672.47301	-672.181948	-672.242301

Amine product of rxn between 29 and a	0.102466	0.110129	0.071393	-248.441594	-248.331465	-248.370201
Amine product of rxn between 30 and a	0.112616	0.121511	0.079726	-361.765339	-361.643828	-361.685613
Amine product of rxn between 31 and a	0.146142	0.154255	0.114912	-326.747402	-326.593147	-326.63249
Amine product of rxn between 32 and a	0.236950	0.253134	0.191910	-1106.239449	-1105.986315	-1106.047539
Amine product of rxn between 33 and a	0.155988	0.165851	0.121255	-440.077631	-439.91178	-439.956376
Amine product of rxn between 37 and a	0.115962	0.126529	0.079767	-948.127675	-948.001146	-948.047908
Amine product of rxn between 38 and a	0.116551	0.125311	0.083691	-512.911664	-512.786353	512.995355
Amine product of rxn between 39 and a	0.092595	0.099446	0.062329	-360.568868	-360.469422	-360.506539
Amine product of rxn between 40 and a	0.122198	0.129965	0.090835	-399.862546	-399.732581	-399.771711

It should be noted that certain ester and amine products of the transformations are the same.

Cartesian coordinates of the structures

1

C	-0.070358	0.167356	-0.623938
N	0.965700	0.366505	-1.509211
N	-1.179146	0.297039	-1.429009
C	0.512785	0.609161	-2.804131
C	2.342896	0.297235	-1.113917
Ni	-0.037010	-0.116926	1.210034
C	-0.842078	0.563822	-2.753497
C	-2.516618	0.141405	-0.935115
H	1.191620	0.787205	-3.623375
C	2.977431	-0.956338	-1.108479
C	2.991813	1.478179	-0.711802
H	-1.591121	0.692437	-3.519086
C	-3.212826	1.280750	-0.493512
C	-3.070113	-1.149493	-0.889037
C	2.264351	-2.219466	-1.568612
C	4.295333	-1.014648	-0.641153
C	2.277001	2.821323	-0.720072
C	4.309965	1.370296	-0.255433
C	-2.570144	2.659374	-0.505522
C	-4.501564	1.095888	0.018560
C	-2.297497	-2.375148	-1.351980
C	-4.363346	-1.284229	-0.370658
C	3.082237	-2.990559	-2.616040
C	1.905209	-3.108583	-0.368013
H	1.324845	-1.921089	-2.042422
C	4.953206	0.134947	-0.211223
H	4.808974	-1.970800	-0.609665
C	3.135266	3.943167	-1.322366
C	1.799377	3.180183	0.696808
H	1.385709	2.720935	-1.346279
H	4.836741	2.260198	0.075066
C	-2.073509	3.019294	0.905188
C	-3.500554	3.744672	-1.065737
H	-1.691902	2.613923	-1.155601
C	-5.072355	-0.173900	0.081170
H	-5.062208	1.952573	0.379329
C	-3.051282	-3.140771	-2.450612
C	-1.961473	-3.285015	-0.159604
H	-1.349337	-2.035802	-1.777035
H	-4.815872	-2.269953	-0.316250
H	3.339815	-2.354135	-3.470708

H	2.506329	-3.845398	-2.990110
H	4.016067	-3.382296	-2.196586
H	1.376971	-4.010209	-0.702122
H	2.806066	-3.423993	0.172804
H	1.263400	-2.564152	0.329257
H	5.975060	0.069848	0.153085
H	3.493280	3.680865	-2.324682
H	2.546543	4.864632	-1.401788
H	4.009925	4.168768	-0.701691
H	1.240016	4.123791	0.688038
H	2.651308	3.296617	1.378494
H	1.151361	2.389761	1.086965
H	-2.911872	3.083356	1.609856
H	-1.558521	3.987690	0.899492
H	-1.375687	2.254282	1.263435
H	-3.864835	3.483346	-2.066074
H	-2.964083	4.698106	-1.137473
H	-4.372956	3.909610	-0.423090
H	-6.074107	-0.297644	0.483879
H	-3.276438	-2.492208	-3.305280
H	-2.446489	-3.981187	-2.811640
H	-3.999453	-3.549874	-2.082396
H	-2.872335	-3.657620	0.325001
H	-1.376355	-4.151667	-0.489962
H	-1.375092	-2.735064	0.582968
C	0.513269	0.504827	3.106795
C	-0.523893	-0.475525	3.132786
C	1.875289	0.110547	3.081787
H	0.267155	1.552720	3.260477
C	-1.959949	-0.113532	3.428028
C	-0.124698	-1.842572	3.054594
C	2.228231	-1.234410	3.091873
H	2.645127	0.876818	3.065430
H	-2.650289	-0.715817	2.826421
H	-2.158194	0.938569	3.204629
H	-2.204673	-0.283444	4.487236
C	1.219119	-2.213783	3.068064
H	-0.896182	-2.609065	3.048613
H	3.274419	-1.525341	3.075429
H	1.484532	-3.266917	3.032798

2

C	-0.819871	0.251926	0.580363
N	-0.083982	0.765004	1.620781

N	-2.081993	0.169040	1.113969
C	-0.865088	1.002370	2.748360
C	1.314768	1.069087	1.509476
Ni	-0.293527	-0.189398	-1.169766
C	-2.128027	0.623317	2.429442
C	-3.217143	-0.297818	0.370439
H	-0.448545	1.412708	3.654849
C	1.689053	2.312053	0.972916
C	2.252115	0.098979	1.905693
H	-3.043278	0.629059	3.000182
C	-3.561233	-1.658886	0.445454
C	-3.931664	0.624491	-0.414136
C	0.664764	3.368497	0.585864
C	3.055622	2.556872	0.796233
C	1.815752	-1.240865	2.479989
C	3.605557	0.389702	1.706954
C	-2.735722	-2.647799	1.255336
C	-4.660615	-2.091656	-0.304467
C	-3.519305	2.085325	-0.512858
C	-5.021918	0.143433	-1.147898
C	0.882545	4.670582	1.373382
C	0.671524	3.620981	-0.929490
H	-0.327575	2.990245	0.845314
C	4.004481	1.601243	1.147019
H	3.377586	3.501174	0.367582
C	2.688200	-1.693257	3.659820
C	1.782306	-2.307849	1.373379
H	0.794133	-1.126776	2.856031
H	4.355007	-0.344997	1.982769
C	-1.820562	-3.456763	0.319762
C	-3.602487	-3.570175	2.124257
H	-2.089002	-2.076594	1.927178
C	-5.384395	-1.200477	-1.093749
H	-4.949485	-3.137688	-0.275150
C	-2.855354	2.358767	-1.872932
C	-4.694790	3.040944	-0.259816
H	-2.768215	2.277227	0.258377
H	-5.589788	0.828067	-1.770556
H	0.859641	4.490079	2.454446
H	0.097289	5.397404	1.132967
H	1.847099	5.132053	1.131892
H	1.649337	3.981478	-1.270673
H	0.437939	2.697333	-1.464904
H	-0.077039	4.377107	-1.195629

H	5.058530	1.797891	0.977922
H	2.268256	-2.600860	4.108930
H	2.746067	-0.923745	4.438426
H	3.710805	-1.930974	3.344719
H	2.777761	-2.452008	0.938983
H	1.436657	-3.268961	1.774739
H	1.111218	-2.003358	0.566284
H	-2.411819	-4.055162	-0.384695
H	-1.184061	-4.139267	0.895717
H	-1.175193	-2.787488	-0.258149
H	-4.267574	-2.995395	2.779110
H	-2.965032	-4.200212	2.755574
H	-4.223582	-4.239071	1.517720
H	-6.234349	-1.555573	-1.670256
H	-3.554771	2.173158	-2.697403
H	-1.984388	1.705892	-2.004595
H	-2.521422	3.401190	-1.937393
H	-5.166136	2.846670	0.710607
H	-4.344081	4.079674	-0.265434
H	-5.467595	2.951270	-1.031908
C	-0.055589	-0.910131	-3.002384
C	0.594995	-2.099721	-2.555999
C	0.662331	0.320158	-2.936401
H	-1.008050	-0.974890	-3.523224
C	1.914749	-2.076535	-2.125925
H	0.057423	-3.042954	-2.593672
C	1.991955	0.329012	-2.465255
H	0.244313	1.226132	-3.365720
C	2.634301	-0.857303	-2.093520
H	2.399544	-2.990211	-1.803394
H	2.543039	1.260731	-2.402060
C	4.030175	-0.771145	-1.628776
O	4.468352	-1.935130	-1.077507
O	4.743969	0.214858	-1.714736
C	5.823142	-1.919461	-0.617767
H	5.965052	-1.157069	0.153255
H	6.513737	-1.720658	-1.442809
H	6.005121	-2.913016	-0.204408

TS3

Ni	-0.480931	-0.231403	1.140829
C	-0.517143	0.258116	-0.659633
C	1.038236	0.133988	2.208567
N	0.348480	0.780146	-1.576409

N	-1.686392	0.129981	-1.358430
O	-0.337393	-0.674323	2.967884
C	2.081657	-0.925426	2.036521
O	1.279263	1.271610	2.591375
C	-0.271712	0.967284	-2.813352
C	1.728199	1.105854	-1.325441
C	-1.556790	0.555926	-2.675632
C	-2.883645	-0.354642	-0.729383
C	-1.014820	0.161653	3.891813
C	3.359761	-0.499212	1.651437
C	1.831041	-2.297427	2.188212
H	0.262441	1.374169	-3.657082
C	2.714394	0.236243	-1.826216
C	2.027330	2.292068	-0.629714
H	-2.380475	0.527672	-3.371323
C	-3.117162	-1.740765	-0.702663
C	-3.736703	0.578071	-0.110180
H	-0.611579	1.182364	3.861989
H	-0.872641	-0.242384	4.903742
H	-2.089753	0.191681	3.671272
C	4.374785	-1.428183	1.426304
H	3.542070	0.561211	1.523974
C	2.851490	-3.221592	1.974384
H	0.844427	-2.623373	2.492949
C	2.331826	-1.069401	-2.513147
C	4.051137	0.598020	-1.627353
C	0.922875	3.208341	-0.116733
C	3.380673	2.604397	-0.455541
C	-2.143332	-2.732233	-1.321642
C	-4.262270	-2.188523	-0.033773
C	-3.416838	2.065887	-0.094558
C	-4.866947	0.080002	0.546591
C	4.126336	-2.792104	1.591334
H	5.359694	-1.084565	1.121573
H	2.652681	-4.281613	2.109073
C	3.401383	-1.590415	-3.480548
C	1.986344	-2.144413	-1.465742
H	1.426926	-0.884850	-3.102475
C	4.380115	1.771941	-0.954243
H	4.842167	-0.045184	-1.996034
C	1.359804	4.097525	1.052187
C	0.352008	4.073047	-1.256667
H	0.123089	2.563874	0.258266
H	3.656351	3.506353	0.078728

C	-2.839932	-3.702546	-2.287055
C	-1.380759	-3.487941	-0.219447
H	-1.405230	-2.167872	-1.898248
C	-5.128200	-1.288609	0.583068
H	-4.471711	-3.252800	0.012447
C	-4.624266	2.930426	-0.484685
C	-2.856845	2.474971	1.279281
H	-2.631025	2.250943	-0.832352
H	-5.544389	0.768790	1.041521
H	4.918144	-3.517044	1.421962
H	4.300343	-1.925325	-2.950611
H	3.699634	-0.828667	-4.210141
H	3.011497	-2.453586	-4.031660
H	2.865871	-2.392856	-0.864770
H	1.639810	-3.059343	-1.962392
H	1.205051	-1.804940	-0.782308
H	5.424152	2.035955	-0.808357
H	0.485726	4.623018	1.454177
H	2.082421	4.862225	0.741393
H	1.791487	3.497823	1.856117
H	-0.469232	4.697739	-0.884497
H	1.124084	4.739092	-1.661835
H	-0.036246	3.468272	-2.081508
H	-3.378059	-3.163941	-3.075618
H	-2.099997	-4.355369	-2.764640
H	-3.559844	-4.345753	-1.768394
H	-0.847759	-2.786140	0.432737
H	-2.066088	-4.075974	0.402781
H	-0.645490	-4.172431	-0.658012
H	-6.010258	-1.656090	1.100275
H	-5.028871	2.635779	-1.459786
H	-4.327655	3.983966	-0.543469
H	-5.433446	2.858865	0.250839
H	-2.572414	3.534121	1.279786
H	-3.602180	2.322614	2.069275
H	-1.969408	1.881700	1.529132

4

Ni	0.406054	-0.760925	-0.956524
C	-1.065138	0.030014	-1.731373
C	0.590114	0.400440	0.533122
C	-2.331082	-0.771356	-1.817601
O	-0.996842	1.162073	-2.179185
O	0.701774	-2.101992	-2.105963

N	-0.246527	1.013943	1.416568
N	1.835520	0.683481	1.012998
C	-3.549006	-0.091042	-1.967167
C	-2.323006	-2.170595	-1.744165
C	0.805030	-1.956349	-3.491550
C	0.464398	1.649750	2.431787
C	-1.681437	0.913590	1.381855
C	1.780847	1.440467	2.177010
C	3.037029	0.201111	0.383943
C	-4.746447	-0.799739	-2.010991
H	-3.542197	0.990595	-2.032151
C	-3.523019	-2.877689	-1.807802
H	-1.364860	-2.677527	-1.676921
H	-0.185578	-1.880956	-3.977192
H	1.387242	-1.069409	-3.798220
H	1.305654	-2.839899	-3.923253
H	-0.035015	2.171903	3.231262
C	-2.273619	-0.247325	1.907589
C	-2.424637	1.977480	0.840022
H	2.671900	1.752148	2.698502
C	3.458664	-1.111449	0.657567
C	3.717451	1.057476	-0.501184
C	-4.735848	-2.194888	-1.927586
H	-5.688465	-0.266675	-2.109231
H	-3.511916	-3.963504	-1.763696
C	-1.449195	-1.361295	2.538557
C	-3.668081	-0.344204	1.843240
C	-1.750991	3.241980	0.325757
C	-3.816696	1.843837	0.828547
C	2.697430	-2.038316	1.594923
C	4.627469	-1.555453	0.027509
C	3.174378	2.436512	-0.845611
C	4.875781	0.563348	-1.108584
H	-5.670738	-2.748272	-1.962979
C	-1.979396	-1.745573	3.929217
C	-1.366547	-2.587275	1.616866
H	-0.429316	-0.989582	2.676590
C	-4.431452	0.690921	1.312414
H	-4.159172	-1.236942	2.217018
C	-2.394993	3.792987	-0.953804
C	-1.734944	4.322274	1.423252
H	-0.716523	2.986461	0.079207
H	-4.426143	2.642642	0.419314
C	3.490931	-2.284806	2.888654

C	2.330827	-3.360971	0.901360
H	1.760682	-1.548516	1.874416
C	5.329257	-0.727896	-0.843380
H	4.984693	-2.562662	0.217293
C	4.276465	3.494652	-0.996448
C	2.306449	2.357894	-2.115850
H	2.525323	2.759745	-0.025470
H	5.425934	1.190148	-1.802422
H	-2.972101	-2.206304	3.875113
H	-2.049835	-0.871277	4.586459
H	-1.306220	-2.471749	4.399764
H	-2.363181	-2.983026	1.391797
H	-0.780532	-3.383563	2.090893
H	-0.888397	-2.325221	0.668449
H	-5.512911	0.597411	1.270261
H	-1.813329	4.648084	-1.317563
H	-3.417933	4.147349	-0.780781
H	-2.406310	3.035393	-1.740553
H	-1.211931	5.219475	1.071317
H	-2.757008	4.611769	1.696315
H	-1.234137	3.975158	2.333113
H	3.710804	-1.344448	3.407435
H	2.919755	-2.924590	3.571915
H	4.445076	-2.783483	2.680820
H	3.225294	-3.948276	0.662597
H	1.786074	-3.184789	-0.032023
H	1.703372	-3.970418	1.562028
H	6.232215	-1.092206	-1.325516
H	4.930283	3.526292	-0.117134
H	3.825243	4.485579	-1.120970
H	4.902765	3.312069	-1.876908
H	2.901499	2.006075	-2.967465
H	1.907469	3.348622	-2.366369
H	1.458626	1.678573	-1.988722

5

C	0.116687	-0.993275	0.465901
N	1.241929	-1.673863	0.826596
N	-0.893532	-1.672842	1.090396
C	0.938737	-2.763118	1.644819
C	2.534971	-1.449052	0.235445
Ni	-0.268239	0.532158	-0.592413
C	-0.404620	-2.761282	1.809658
C	-2.297260	-1.394462	0.905524

H	1.702779	-3.433461	2.003184
C	2.854607	-2.186782	-0.918262
C	3.413463	-0.516746	0.820173
C	0.155129	1.693894	0.821333
H	-1.057736	-3.424116	2.352085
C	-2.995220	-0.698327	1.908649
C	-2.916064	-1.865774	-0.271885
C	1.973851	-3.317134	-1.439752
C	4.070324	-1.896851	-1.550834
C	3.089459	0.153076	2.150524
C	4.619130	-0.273405	0.154061
C	1.320527	2.595062	0.449888
O	-0.422174	1.892882	1.885517
C	-2.328594	-0.268574	3.206782
C	-4.356863	-0.450137	1.692846
C	-2.161276	-2.706883	-1.292392
C	-4.276932	-1.583948	-0.437680
C	1.392294	-3.029885	-2.831030
C	2.748816	-4.646792	-1.415202
H	1.123864	-3.430579	-0.762743
C	4.934975	-0.939534	-1.029724
H	4.340606	-2.433382	-2.455382
C	3.895324	1.431838	2.410697
C	3.311982	-0.828633	3.318335
H	2.028831	0.421346	2.141460
H	5.316042	0.451552	0.558094
C	2.166141	2.275069	-0.620096
C	1.530350	3.788367	1.155124
C	-2.812624	1.100996	3.701694
C	-2.550160	-1.331941	4.299097
H	-1.257659	-0.177714	3.014625
C	-4.990011	-0.880068	0.530816
H	-4.924322	0.089253	2.443635
C	-2.649607	-2.507922	-2.731067
C	-2.221447	-4.194022	-0.896923
H	-1.121030	-2.380931	-1.287034
H	-4.785458	-1.923054	-1.333251
H	2.178000	-2.789751	-3.557509
H	0.860130	-3.915760	-3.200224
H	0.684952	-2.198040	-2.777368
H	3.156932	-4.858084	-0.419720
H	3.585622	-4.639004	-2.123164
H	2.085601	-5.474106	-1.694174
H	5.871334	-0.722470	-1.536746

H	3.487428	1.948253	3.285986
H	4.946945	1.205197	2.626351
H	3.860506	2.122792	1.566738
H	3.087065	-0.336524	4.271941
H	4.355856	-1.163884	3.347099
H	2.673583	-1.712360	3.243635
C	3.207861	3.130188	-0.979401
H	2.013458	1.345096	-1.160322
C	2.562106	4.650565	0.791625
H	0.870293	4.017986	1.986116
H	-2.202000	1.420102	4.553841
H	-3.855200	1.071388	4.040642
H	-2.712652	1.856187	2.919705
H	-2.052297	-1.033287	5.229466
H	-3.619252	-1.454659	4.512079
H	-2.156041	-2.310799	4.007677
H	-6.046147	-0.672105	0.380611
H	-2.002720	-3.072255	-3.412094
H	-3.674622	-2.868202	-2.883342
H	-2.576608	-1.454470	-3.009482
H	-1.642186	-4.799598	-1.604425
H	-3.255493	-4.561317	-0.905599
H	-1.813265	-4.367107	0.104231
C	3.404058	4.321926	-0.276763
H	3.868148	2.863022	-1.799879
H	2.716759	5.576850	1.338941
H	4.211716	4.992572	-0.558010
N	-1.414881	1.984108	-1.462337
C	-0.985953	3.359245	-1.787107
C	-2.728045	1.997953	-0.774745
H	-1.500557	1.416082	-2.313709
C	-2.075046	4.102511	-2.554443
H	-0.059922	3.315487	-2.365698
H	-0.774778	3.877504	-0.847255
C	-3.754721	2.797886	-1.568546
H	-3.062126	0.966393	-0.646432
H	-2.581313	2.438483	0.215403
O	-3.299167	4.120679	-1.830630
H	-1.789945	5.146700	-2.715579
H	-2.227387	3.628758	-3.540597
H	-4.687860	2.891516	-1.004801
H	-3.975432	2.282038	-2.519824
C	0.417949	0.172953	-3.285375
H	0.222344	-0.241995	-4.293287

H	1.479763	-0.056110	-3.060010
H	0.371325	1.280099	-3.403333
O	-0.483103	-0.339020	-2.357155

TS6

C	-0.304724	-1.071160	0.053948
N	0.641931	-2.053692	0.045866
N	-1.467227	-1.742877	0.313358
C	0.080397	-3.308709	0.271875
C	2.058905	-1.841613	-0.087570
Ni	-0.298832	0.801555	-0.263481
C	-1.247677	-3.110965	0.447913
C	-2.755306	-1.126868	0.514706
H	0.679939	-4.203523	0.288851
C	2.622564	-1.828219	-1.374138
C	2.819242	-1.681753	1.085657
C	0.968519	1.267856	1.004193
H	-2.050114	-3.796398	0.664812
C	-3.721906	-1.243289	-0.497488
C	-2.999354	-0.463961	1.735634
C	1.764227	-2.021312	-2.615459
C	4.006714	-1.654109	-1.469758
C	2.183053	-1.757780	2.468023
C	4.197873	-1.496360	0.934177
C	2.315103	1.679223	0.460262
O	0.752050	1.360347	2.207649
C	-3.433906	-1.942555	-1.817145
C	-4.986017	-0.694111	-0.251860
C	-1.934385	-0.366497	2.821318
C	-4.274769	0.080687	1.921662
C	2.197162	-1.134266	-3.790982
C	1.744863	-3.502241	-3.034383
H	0.740943	-1.737489	-2.355627
C	4.785196	-1.483946	-0.327843
H	4.478504	-1.633643	-2.446584
C	2.809526	-0.791941	3.481816
C	2.245693	-3.198755	3.009135
H	1.130644	-1.479501	2.366571
H	4.817694	-1.353471	1.812403
C	2.643896	1.462231	-0.880928
C	3.239882	2.323565	1.294398
C	-3.962859	-1.147015	-3.020643
C	-4.010542	-3.369633	-1.824406
H	-2.347557	-2.012938	-1.922751

C	-5.260407	-0.038230	0.943343
H	-5.757315	-0.770704	-1.011298
C	-2.132563	0.822073	3.768119
C	-1.844769	-1.675736	3.627985
H	-0.971889	-0.198708	2.335128
H	-4.501701	0.602812	2.844102
H	1.469293	-1.213350	-4.606319
H	3.170510	-1.434747	-4.194965
H	2.262849	-0.081507	-3.498313
H	1.100702	-3.647675	-3.909963
H	2.753088	-3.847438	-3.293350
H	1.368127	-4.140765	-2.228469
H	5.856653	-1.331757	-0.422508
H	2.226434	-0.810774	4.409619
H	3.837245	-1.075793	3.737197
H	2.807682	0.232846	3.107753
H	1.750397	-3.259376	3.985453
H	3.286871	-3.519382	3.136046
H	1.755136	-3.911111	2.339313
C	3.878820	1.866258	-1.384314
H	1.920211	0.967525	-1.522328
C	4.473923	2.732853	0.795444
H	2.965545	2.502256	2.329219
H	-5.057601	-1.169534	-3.074159
H	-3.584682	-1.582707	-3.953260
H	-3.637118	-0.106644	-2.960828
H	-5.101223	-3.348305	-1.711172
H	-3.780382	-3.873006	-2.771296
H	-3.604946	-3.980729	-1.011313
H	-6.245375	0.387855	1.114285
H	-1.250973	0.922431	4.408582
H	-3.004784	0.693768	4.421254
H	-2.247220	1.759535	3.215909
H	-1.055823	-1.597864	4.385880
H	-2.790613	-1.881768	4.144724
H	-1.613324	-2.536736	2.993681
C	4.796474	2.503025	-0.545916
H	4.128752	1.680224	-2.425264
H	5.184588	3.235178	1.446584
H	5.759605	2.821718	-0.935852
N	-0.769180	2.662247	-0.568159
C	0.151629	3.720336	-0.967674
C	-1.663625	3.151329	0.482887
C	-0.625083	4.958775	-1.412172

H	0.786862	3.369735	-1.788478
H	0.818667	4.006997	-0.137223
C	-2.398678	4.408306	0.019615
H	-2.395921	2.373319	0.729112
H	-1.100378	3.382393	1.402308
O	-1.487459	5.426477	-0.381280
H	0.052577	5.786829	-1.647548
H	-1.215920	4.719386	-2.314394
H	-3.001527	4.834450	0.828994
H	-3.068422	4.154742	-0.821032
C	-0.986277	0.638564	-3.154881
H	-0.875345	-0.435513	-3.339238
H	-1.574722	1.065158	-3.980468
H	0.015737	1.094568	-3.181544
O	-1.634554	0.840421	-1.912555
H	-1.446915	1.873560	-1.560035

7

Ni	-0.304071	0.797693	-0.277919
C	0.947837	1.288581	0.993577
C	-0.296559	-1.075343	0.060777
C	2.294790	1.707085	0.455378
O	0.724229	1.378692	2.195227
N	-0.783549	2.645159	-0.585663
N	0.654997	-2.053156	0.069540
N	-1.454255	-1.747766	0.341430
C	2.637997	1.473052	-0.879054
C	3.206177	2.370456	1.289281
C	0.132453	3.706691	-0.980746
C	-1.675890	3.135234	0.464791
C	0.101843	-3.305244	0.329673
C	2.068490	-1.836355	-0.087936
C	-1.226413	-3.110675	0.508469
C	-2.741259	-1.127412	0.537853
C	3.876726	1.874444	-1.374820
H	1.921516	0.967331	-1.519720
C	4.443383	2.778860	0.797269
H	2.919157	2.563388	2.318083
C	-0.650029	4.937004	-1.438912
H	0.777061	3.359523	-1.796157
H	0.792734	4.007889	-0.148529
C	-2.423981	4.381397	-0.008683
H	-2.402379	2.355248	0.722635
H	-1.115113	3.383815	1.382650

H	0.706287	-4.196357	0.363648
C	2.611338	-1.836759	-1.383759
C	2.846438	-1.653628	1.070057
H	-2.023115	-3.795129	0.747672
C	-3.702835	-1.229069	-0.481679
C	-2.985913	-0.467547	1.759812
C	4.782218	2.528244	-0.536280
H	4.138983	1.674541	-2.410120
H	5.143888	3.295990	1.447794
O	-1.523236	5.405844	-0.417640
H	0.022603	5.769065	-1.675285
H	-1.232930	4.686660	-2.343498
H	-3.031651	4.808603	0.796736
H	-3.090253	4.113491	-0.847845
C	1.732187	-2.048490	-2.607559
C	3.992307	-1.653683	-1.504247
C	2.233827	-1.713894	2.463444
C	4.220640	-1.457137	0.893615
C	-3.416982	-1.940422	-1.795596
C	-4.959257	-0.659316	-0.244005
C	-1.928103	-0.391963	2.854043
C	-4.254857	0.094089	1.939679
H	5.748102	2.845312	-0.920701
C	2.157602	-1.192708	-3.808546
C	1.690599	-3.537855	-2.993830
H	0.716392	-1.747844	-2.336735
C	4.787643	-1.459543	-0.377603
H	4.448797	-1.643065	-2.488396
C	2.868838	-0.726868	3.450973
C	2.319295	-3.145188	3.027105
H	1.177511	-1.446261	2.375224
H	4.852326	-1.292518	1.759624
C	-3.986571	-1.185642	-3.006275
C	-3.955266	-3.382638	-1.770163
H	-2.330810	-1.982578	-1.916756
C	-5.232822	-0.003443	0.951796
H	-5.725852	-0.719597	-1.009437
C	-2.098615	0.811184	3.787890
C	-1.887276	-1.697013	3.670983
H	-0.957619	-0.257458	2.374150
H	-4.481835	0.614497	2.863129
H	1.414624	-1.275876	-4.609714
H	3.118519	-1.518271	-4.222799
H	2.246641	-0.135481	-3.538543

H	1.033568	-3.694180	-3.857878
H	2.691741	-3.899110	-3.258021
H	1.316783	-4.154914	-2.170063
H	5.855906	-1.298813	-0.492066
H	2.297684	-0.729236	4.386198
H	3.900675	-1.002740	3.698331
H	2.860020	0.290232	3.057256
H	1.838521	-3.194779	4.011342
H	3.365585	-3.452563	3.144686
H	1.827643	-3.874727	2.376716
H	-5.080523	-1.245344	-3.045106
H	-3.606926	-1.628448	-3.934854
H	-3.694229	-0.134125	-2.974184
H	-5.043801	-3.387753	-1.636551
H	-3.729442	-3.894321	-2.713681
H	-3.518632	-3.970421	-0.956728
H	-6.212137	0.438169	1.115639
H	-1.221380	0.889282	4.437409
H	-2.981654	0.716767	4.432099
H	-2.175627	1.746208	3.225582
H	-1.100637	-1.640770	4.433144
H	-2.842961	-1.867777	4.182314
H	-1.680319	-2.568822	3.042926
C	-0.988818	0.586312	-3.207202
H	-1.561283	1.038932	-4.029035
H	0.022523	1.017504	-3.212539
H	-0.906322	-0.487435	-3.399373
O	-1.645282	0.780395	-1.964020
H	-1.507516	1.780963	-1.652025

8

Ni	0.519386	-0.229749	-0.763026
N	1.714742	-1.004071	-1.876561
C	-0.143028	0.807743	0.680475
C	1.765242	-0.743380	-3.304507
C	2.226174	-2.338101	-1.608938
C	-1.004277	-0.930631	-1.527659
N	-1.289102	1.479817	0.987572
N	0.700888	1.128473	1.710009
C	3.206064	-0.875006	-3.805171
H	1.392885	0.265125	-3.515225
H	1.138512	-1.456305	-3.873498
C	3.663281	-2.453285	-2.125065
H	2.217134	-2.535967	-0.531005

H	1.617420	-3.120696	-2.103090
C	-1.529014	-2.225289	-0.967110
O	-1.523371	-0.423663	-2.509130
C	-1.150699	2.222247	2.157432
C	-2.466838	1.495565	0.162322
C	0.106075	1.996928	2.617034
C	2.066882	0.678902	1.748537
O	3.735952	-2.165814	-3.517314
H	3.262370	-0.756011	-4.893013
H	3.830869	-0.099079	-3.330579
H	4.051016	-3.470776	-2.000220
H	4.305263	-1.755578	-1.560636
C	-0.973112	-2.787514	0.186247
C	-2.575003	-2.890046	-1.622950
H	-1.948587	2.840852	2.533709
C	-2.479408	2.336634	-0.961431
C	-3.550124	0.670285	0.515711
H	0.630915	2.371220	3.481303
C	3.039954	1.450176	1.091278
C	2.362126	-0.532863	2.401258
C	-1.454068	-3.997195	0.685531
H	-0.158683	-2.264355	0.677485
C	-3.053832	-4.101551	-1.129863
H	-2.996717	-2.436418	-2.514218
C	-1.295772	3.215384	-1.337383
C	-3.635068	2.340236	-1.749760
C	-3.512980	-0.216517	1.753136
C	-4.682856	0.713104	-0.303622
C	2.698342	2.739223	0.359031
C	4.357462	0.977298	1.108315
C	1.277595	-1.329520	3.112040
C	3.693162	-0.960664	2.391730
C	-2.494694	-4.656257	0.026549
H	-1.016724	-4.427771	1.582014
H	-3.863265	-4.615424	-1.641573
C	-1.671027	4.705115	-1.288273
C	-0.726215	2.821621	-2.709797
H	-0.503974	3.055565	-0.601620
C	-4.724914	1.538245	-1.425656
H	-3.675064	2.971393	-2.632393
C	-4.178404	-1.582048	1.531490
C	-4.162733	0.494672	2.954796
H	-2.461782	-0.401323	1.997397
H	-5.537850	0.088415	-0.067214

C	3.478477	3.933518	0.930956
C	2.928530	2.589776	-1.153783
H	1.634487	2.943592	0.505615
C	4.681104	-0.213631	1.750847
H	5.132839	1.544229	0.602298
C	1.474192	-2.847635	3.001349
C	1.175151	-0.904224	4.588067
H	0.323510	-1.088623	2.633168
H	3.961615	-1.890302	2.882156
H	-2.870038	-5.600530	0.411801
H	-2.035254	4.992125	-0.294668
H	-0.798227	5.326121	-1.523279
H	-2.456079	4.945214	-2.014741
H	0.191299	3.385720	-2.915415
H	-1.440551	3.040518	-3.512863
H	-0.508151	1.751371	-2.744111
H	-5.611820	1.551043	-2.053285
H	-3.968838	-2.239612	2.382575
H	-5.268224	-1.492788	1.451680
H	-3.809255	-2.067388	0.626503
H	-5.217965	0.711043	2.749002
H	-4.115800	-0.140455	3.847658
H	-3.668434	1.442416	3.189900
H	3.291959	4.054425	2.004468
H	3.178365	4.859728	0.426909
H	4.558884	3.813660	0.789850
H	3.985188	2.402096	-1.378392
H	2.348898	1.751679	-1.551852
H	2.629503	3.506260	-1.676342
H	5.708581	-0.566746	1.750073
H	0.597435	-3.364837	3.407179
H	2.344504	-3.191017	3.572048
H	1.607336	-3.163326	1.961248
H	0.372941	-1.455417	5.093110
H	2.113543	-1.107029	5.118042
H	0.960337	0.165324	4.682205

TS9

Ni	-0.487149	0.275129	-0.716752
C	-0.018740	-0.909097	0.653237
N	-0.883099	1.556849	-2.013184
N	1.076673	-1.654373	0.980710
N	-0.940296	-1.259657	1.603408
C	0.986003	1.123265	-1.532941

C	-1.092166	1.229520	-3.410603
C	-1.474810	2.843963	-1.693578
C	0.831640	-2.463928	2.088230
C	2.302334	-1.678598	0.227152
C	-0.441985	-2.212594	2.484755
C	-2.278266	-0.734394	1.575318
C	1.559066	2.304587	-0.802933
O	1.591151	0.572691	-2.447981
C	-2.591549	1.210429	-3.709276
H	-0.644801	0.258182	-3.637490
H	-0.612397	1.980686	-4.064590
C	-2.971877	2.801624	-2.011380
H	-1.331788	3.079600	-0.635640
H	-1.010072	3.652039	-2.287588
H	1.577750	-3.140459	2.471537
C	2.350269	-2.447689	-0.946634
C	3.403718	-0.944105	0.703162
H	-1.035407	-2.619097	3.288051
C	-3.238176	-1.400540	0.793686
C	-2.554948	0.453065	2.279139
C	1.055331	2.728543	0.432325
C	2.641562	2.991947	-1.369850
O	-3.201838	2.454036	-3.372378
H	-2.784869	1.057379	-4.777027
H	-3.065634	0.393021	-3.140417
H	-3.438995	3.782395	-1.864458
H	-3.459136	2.069947	-1.344011
C	1.139565	-3.191963	-1.487725
C	3.566548	-2.498923	-1.636515
C	3.317833	-0.096182	1.964332
C	4.596106	-1.027602	-0.023059
C	-2.898157	-2.650440	-0.005035
C	-4.521385	-0.843538	0.732858
C	-1.484650	1.137831	3.116024
C	-3.853772	0.963978	2.193930
C	1.606109	3.831411	1.083504
H	0.233800	2.174056	0.875126
C	3.193090	4.095945	-0.721615
H	3.035568	2.641998	-2.318655
C	1.356719	-4.713007	-1.463254
C	0.782120	-2.691816	-2.897092
H	0.286469	-2.971074	-0.842251
C	4.679554	-1.801566	-1.178363
H	3.635211	-3.082783	-2.549373

C	4.059838	1.241270	1.827033
C	3.841567	-0.870528	3.187488
H	2.261261	0.133395	2.135994
H	5.466446	-0.473871	0.313033
C	-3.911322	-3.781840	0.225555
C	-2.772038	-2.314353	-1.500857
H	-1.923024	-3.013890	0.331911
C	-4.826775	0.323665	1.427176
H	-5.285171	-1.324369	0.129825
C	-1.585580	2.668843	3.087066
C	-1.519025	0.620036	4.565356
H	-0.513310	0.867619	2.690904
H	-4.107415	1.876558	2.722846
C	2.675082	4.520493	0.505827
H	1.205250	4.150483	2.041854
H	4.029662	4.625364	-1.170243
H	1.564979	-5.070314	-0.447716
H	0.463058	-5.232798	-1.829726
H	2.198787	-5.007611	-2.100663
H	-0.165773	-3.131447	-3.229472
H	1.552584	-2.972912	-3.625400
H	0.700135	-1.601439	-2.904465
H	5.614550	-1.850580	-1.729784
H	3.820688	1.888899	2.677787
H	5.147309	1.101905	1.821134
H	3.777616	1.764019	0.910884
H	4.897128	-1.136173	3.053046
H	3.760140	-0.258289	4.093841
H	3.283277	-1.796958	3.356239
H	-4.013938	-4.015562	1.291396
H	-3.583921	-4.690690	-0.292296
H	-4.904456	-3.524751	-0.159806
H	-3.718747	-1.929389	-1.897815
H	-2.000016	-1.552976	-1.668177
H	-2.500688	-3.208507	-2.074340
H	-5.827854	0.741598	1.367726
H	-0.712964	3.106600	3.584786
H	-2.474549	3.031933	3.615235
H	-1.622132	3.051049	2.061615
H	-0.726347	1.088678	5.160641
H	-2.481226	0.850361	5.038402
H	-1.375014	-0.464668	4.606476
H	3.107679	5.379425	1.011835

10

Ni	0.341895	-0.406329	-0.683059
C	0.153814	0.893180	0.620898
N	0.413120	-1.776384	-2.026434
N	-0.840593	1.773133	0.961703
N	1.157157	1.199614	1.508542
C	-1.021526	-1.480233	-1.845671
C	0.970651	-1.300743	-3.311414
C	0.967686	-3.112200	-1.714897
C	-0.457418	2.603720	2.014081
C	-2.091186	1.902277	0.264965
C	0.801975	2.240218	2.362019
C	2.444085	0.565352	1.446499
C	-1.798271	-2.367990	-0.903260
O	-1.622196	-0.837622	-2.733768
C	2.491075	-1.286675	-3.227987
H	0.583176	-0.304573	-3.516910
H	0.639896	-1.970557	-4.118393
C	2.488937	-3.035636	-1.679940
H	0.595426	-3.462012	-0.755329
H	0.647550	-3.822882	-2.491127
H	-1.111616	3.367686	2.401648
C	-2.114178	2.600751	-0.954416
C	-3.248131	1.348685	0.842049
H	1.474276	2.619826	3.115085
C	3.464000	1.195187	0.711641
C	2.619424	-0.678116	2.082723
C	-1.369128	-2.734972	0.382069
C	-3.046468	-2.827422	-1.346738
O	3.019149	-2.570644	-2.913118
H	2.918428	-1.002598	-4.194543
H	2.810392	-0.555692	-2.470188
H	2.911004	-4.031785	-1.513559
H	2.802749	-2.374752	-0.856089
C	-0.853797	3.143799	-1.608025
C	-3.353512	2.769847	-1.581626
C	-3.194865	0.556665	2.139752
C	-4.463821	1.551103	0.180292
C	3.222169	2.505510	-0.025038
C	4.698089	0.539531	0.622564
C	1.491016	-1.315735	2.880037
C	3.871718	-1.290527	1.968723
C	-2.139216	-3.579457	1.178656
H	-0.438863	-2.322924	0.761516

C	-3.823120	-3.666777	-0.547440
H	-3.390162	-2.514171	-2.326672
C	-0.856119	4.679963	-1.646288
C	-0.668517	2.540332	-3.009781
H	0.001547	2.828797	-1.007836
C	-4.518625	2.260149	-1.017032
H	-3.401889	3.302368	-2.526623
C	-4.059421	-0.710904	2.078188
C	-3.601997	1.428988	3.340155
H	-2.158382	0.238968	2.289620
H	-5.375698	1.139198	0.600192
C	2.921643	2.231903	-1.509205
C	4.380229	3.501370	0.131437
H	2.332066	2.975263	0.403003
C	4.900650	-0.689317	1.244643
H	5.504909	0.990231	0.053623
C	1.509885	-2.848988	2.834566
C	1.507489	-0.816086	4.335955
H	0.550536	-0.989327	2.427661
H	4.045219	-2.249983	2.444127
C	-3.367726	-4.056486	0.713774
H	-1.787306	-3.852120	2.170139
H	-4.785382	-4.017663	-0.911031
H	-1.694238	5.064827	-2.239556
H	0.071450	5.054033	-2.096907
H	-0.938012	5.103437	-0.638184
H	0.316313	2.806975	-3.412669
H	-1.425462	2.915267	-3.709571
H	-0.759788	1.450708	-2.973396
H	-5.471950	2.403322	-1.518497
H	-3.856192	-1.346226	2.947859
H	-5.129716	-0.473214	2.092674
H	-3.851461	-1.290427	1.176392
H	-4.632648	1.786988	3.227791
H	-3.544150	0.853600	4.272174
H	-2.953666	2.305243	3.444101
H	2.694422	3.167110	-2.035019
H	2.062581	1.559120	-1.610356
H	3.783354	1.766018	-2.002519
H	4.110877	4.461683	-0.323221
H	4.616781	3.679275	1.186767
H	5.293038	3.152066	-0.364266
H	5.864325	-1.184527	1.162937
H	1.556214	-3.220562	1.805157

H	0.599776	-3.245607	3.298576
H	2.360970	-3.267729	3.383952
H	0.676533	-1.254021	4.902037
H	2.443552	-1.095286	4.834918
H	1.410391	0.273430	4.386024
H	-3.972595	-4.709585	1.336970

11

N	1.095974	0.453408	0.121287
C	1.218855	-0.757192	0.934932
C	2.370987	1.149313	-0.047947
C	2.282933	-1.666314	0.327348
H	0.261582	-1.271574	1.002943
H	1.529543	-0.474862	1.952104
C	-0.068984	1.141688	-0.116714
C	3.395063	0.171057	-0.616078
H	2.217877	1.995388	-0.718310
H	2.719654	1.534102	0.921670
O	3.525602	-0.988256	0.200197
H	2.462424	-2.530181	0.974081
H	1.943899	-2.026713	-0.658352
C	-1.359400	0.375503	-0.066425
O	-0.070950	2.336275	-0.415490
H	4.385105	0.634259	-0.657911
H	3.097178	-0.118393	-1.637175
C	-1.511340	-0.869374	-0.692143
C	-2.469043	0.983492	0.533052
C	-2.752567	-1.505597	-0.699139
H	-0.662912	-1.326200	-1.193374
C	-3.704296	0.338612	0.541969
H	-2.351703	1.963187	0.985090
C	-3.848256	-0.907390	-0.073577
H	-2.864955	-2.464740	-1.196711
H	-4.557035	0.811072	1.021236
H	-4.813402	-1.406143	-0.073614

12

Ni	0.516853	0.195100	-1.231030
C	-0.100102	-1.439972	-2.131319
C	0.909401	0.572028	0.551976
C	-1.499770	-1.930630	-1.801308
O	0.101350	-0.443863	-2.934943
O	0.764575	-2.524815	-2.133255
N	2.092012	0.361125	1.211637

N	0.166137	1.272429	1.468935
C	-2.493173	-0.781972	-1.652448
H	-1.440473	-2.528688	-0.887555
H	-1.821453	-2.612652	-2.603588
C	2.092436	-2.250924	-2.569932
C	2.087023	0.917363	2.486389
C	3.190118	-0.365183	0.639411
C	0.870477	1.497257	2.647430
C	-1.185041	1.691882	1.220864
C	-3.911225	-1.161039	-1.285884
H	-2.501491	-0.201812	-2.581970
H	-2.105773	-0.093748	-0.888006
H	2.115533	-2.002684	-3.636745
H	2.523031	-1.417913	-2.003113
H	2.666982	-3.161521	-2.388776
H	2.939765	0.849904	3.143140
C	4.089620	0.320528	-0.195300
C	3.303816	-1.737250	0.918630
H	0.444045	2.046075	3.472250
C	-1.405646	2.790503	0.376191
C	-2.234284	0.955462	1.808303
C	-4.917349	-0.187096	-1.389138
C	-4.270207	-2.424365	-0.799522
C	3.916247	1.796944	-0.518633
C	5.135639	-0.416338	-0.761743
C	2.300266	-2.467997	1.797640
C	4.375307	-2.427655	0.340557
C	-0.265798	3.585769	-0.242922
C	-2.734606	3.140543	0.103223
C	-1.947323	-0.256448	2.687793
C	-3.541547	1.345541	1.505680
C	-6.228993	-0.456553	-1.005538
H	-4.655706	0.797917	-1.766902
C	-5.585400	-2.700870	-0.412862
H	-3.521092	-3.205388	-0.718224
C	5.208612	2.596966	-0.298549
C	3.387415	1.974426	-1.951692
H	3.161038	2.202822	0.160742
C	5.280928	-1.776089	-0.492953
H	5.841852	0.078419	-1.421466
C	1.635779	-3.630326	1.043184
C	2.955770	-2.947672	3.103111
H	1.508365	-1.763705	2.064223
H	4.491758	-3.489491	0.535583

C	-0.341540	5.072452	0.139297
C	-0.233532	3.402785	-1.768453
H	0.675401	3.193765	0.151334
C	-3.789844	2.425618	0.658997
H	-2.939867	3.980537	-0.553480
C	-3.050399	-0.539428	3.715393
C	-1.691665	-1.502793	1.820306
H	-1.027621	-0.053944	3.246610
H	-4.376555	0.792074	1.918828
C	-6.569766	-1.718633	-0.509915
H	-6.987785	0.316931	-1.094780
H	-5.837089	-3.689572	-0.037658
H	5.584765	2.474269	0.723610
H	5.024810	3.664469	-0.467563
H	6.002463	2.287558	-0.987784
H	3.216428	3.034723	-2.172422
H	4.101285	1.583835	-2.686875
H	2.441059	1.434432	-2.088246
H	6.101282	-2.330884	-0.940048
H	0.858862	-4.088939	1.666750
H	2.361745	-4.413673	0.793361
H	1.176085	-3.284382	0.112845
H	2.215412	-3.439180	3.745497
H	3.757944	-3.668609	2.904955
H	3.390315	-2.111888	3.664014
H	-0.344944	5.203604	1.227518
H	0.522031	5.613075	-0.266018
H	-1.245827	5.547622	-0.258137
H	-1.148632	3.785579	-2.235634
H	-0.147598	2.340217	-2.033549
H	0.618757	3.936716	-2.204307
H	-4.814572	2.703954	0.430119
H	-2.724799	-1.332888	4.397666
H	-3.287315	0.347210	4.314774
H	-3.973647	-0.884222	3.236153
H	-2.605066	-1.783761	1.286262
H	-1.390140	-2.349688	2.448932
H	-0.907891	-1.322560	1.080297
H	-7.591867	-1.933185	-0.209782

TS13

Ni	-0.703577	0.321052	-1.117375
C	-1.150713	0.125641	0.678810
C	0.668276	-0.770972	-1.841378

N	-0.410323	0.135473	1.828512
N	-2.437452	0.021466	1.131180
O	-0.068046	0.350782	-2.905427
C	2.028949	-0.181538	-1.523226
O	0.481750	-1.960816	-2.084030
C	-1.215955	0.042673	2.960166
C	1.022989	0.230279	1.848013
C	-2.498158	-0.028744	2.519685
C	-3.560330	-0.053017	0.236984
C	-0.928284	-0.251423	-3.864503
C	3.153468	-0.713371	-2.435651
H	1.991205	0.908632	-1.553493
H	2.253187	-0.467778	-0.493747
H	-0.800006	0.036510	3.955037
C	1.617341	1.490727	1.664985
C	1.773146	-0.947760	2.012418
H	-3.434251	-0.112097	3.048782
C	-4.020218	-1.321989	-0.156182
C	-4.118077	1.144250	-0.244492
H	-1.927212	0.201188	-3.821835
H	-0.507706	-0.092811	-4.866637
H	-1.011198	-1.329719	-3.680842
C	4.500845	-0.359825	-1.856932
H	3.047613	-1.799394	-2.518754
H	3.033694	-0.292461	-3.441166
C	0.798211	2.761879	1.495127
C	3.015240	1.546817	1.628699
C	1.121012	-2.312805	2.176815
C	3.168209	-0.834474	1.987190
C	-3.357798	-2.604015	0.324697
C	-5.086715	-1.368831	-1.060886
C	-3.559310	2.504435	0.145210
C	-5.181594	1.044495	-1.148560
C	5.024561	0.933952	-1.986699
C	5.216709	-1.293092	-1.095782
C	0.971631	3.349633	0.085858
C	1.141734	3.796843	2.578699
H	-0.257800	2.503447	1.611466
C	3.785086	0.397776	1.786240
H	3.506954	2.500411	1.462857
C	1.521674	-3.268805	1.040874
C	1.443607	-2.916238	3.554129
H	0.036965	-2.182293	2.120704
H	3.777052	-1.726782	2.094607

C	-4.378150	-3.599770	0.897632
C	-2.529921	-3.239645	-0.806270
H	-2.665016	-2.346722	1.130715
C	-5.662534	-0.199205	-1.551941
H	-5.461814	-2.332039	-1.392477
C	-4.651180	3.466577	0.635673
C	-2.770498	3.109775	-1.028511
H	-2.856981	2.358339	0.970854
H	-5.632945	1.948734	-1.545432
C	6.226307	1.286190	-1.372025
H	4.480064	1.671128	-2.572703
C	6.422643	-0.948527	-0.483199
H	4.818043	-2.298276	-0.981109
H	2.018354	3.607950	-0.113700
H	0.372262	4.261084	-0.025614
H	0.646226	2.629727	-0.672739
H	1.000101	3.382659	3.583637
H	0.497807	4.678804	2.479701
H	2.181441	4.134648	2.499616
H	4.867058	0.458970	1.728543
H	2.601427	-3.460660	1.040997
H	1.014946	-4.233501	1.167184
H	1.243231	-2.867114	0.062011
H	1.130344	-2.250918	4.367351
H	2.518839	-3.097856	3.668723
H	0.927902	-3.875359	3.681565
H	-3.860753	-4.476866	1.303353
H	-4.969332	-3.149675	1.703788
H	-5.073991	-3.955847	0.129452
H	-2.035301	-4.150536	-0.447037
H	-3.171512	-3.517347	-1.651849
H	-1.754510	-2.561136	-1.174177
H	-6.488341	-0.257154	-2.255776
H	-5.214247	3.036801	1.471920
H	-4.201061	4.406622	0.975425
H	-5.364651	3.712251	-0.158787
H	-3.420691	3.279541	-1.894980
H	-1.959709	2.440080	-1.346158
H	-2.323825	4.069236	-0.742608
C	6.931293	0.345269	-0.616826
H	6.616285	2.294413	-1.486230
H	6.965191	-1.690343	0.097473
H	7.870643	0.616755	-0.142656

14

Ni	0.579257	-0.964161	-0.632467
C	-0.819066	-0.118709	-1.466327
C	1.169411	0.453708	0.464713
C	-2.177238	-0.702610	-1.104495
O	-0.665879	0.749181	-2.301306
O	0.400386	-2.565794	-1.421407
N	0.563387	1.353583	1.289625
N	2.500323	0.656812	0.681107
C	-3.338603	-0.145769	-1.943130
H	-2.082431	-1.789106	-1.214646
H	-2.349185	-0.522279	-0.037834
C	0.594670	-2.745221	-2.792900
C	1.497947	2.085571	2.018157
C	-0.861110	1.443687	1.464954
C	2.722974	1.644015	1.633742
C	3.515921	-0.114652	0.014196
C	-4.658824	-0.744113	-1.521997
H	-3.142526	-0.355565	-3.001457
H	-3.369650	0.942449	-1.836887
H	1.468398	-2.199031	-3.193181
H	-0.283033	-2.428337	-3.388858
H	0.753862	-3.815366	-3.012430
H	1.200230	2.831603	2.736759
C	-1.477820	0.534852	2.341813
C	-1.574024	2.415654	0.739219
H	3.717140	1.933337	1.935378
C	4.143214	0.440811	-1.115443
C	3.807860	-1.401800	0.498830
C	-5.596155	0.009492	-0.804836
C	-4.960160	-2.084062	-1.806446
C	-0.690421	-0.530681	3.090551
C	-2.868806	0.613710	2.478583
C	-0.864381	3.405506	-0.174225
C	-2.960958	2.459104	0.920004
C	3.721579	1.791319	-1.675067
C	5.117049	-0.332886	-1.754882
C	3.103558	-1.998312	1.709450
C	4.793470	-2.132897	-0.174824
C	-6.799565	-0.555906	-0.378720
H	-5.376081	1.049587	-0.581154
C	-6.160363	-2.654085	-1.383073
H	-4.245371	-2.683073	-2.366352
C	-1.009453	-0.534637	4.593419

C	-0.928208	-1.916758	2.467396
H	0.374535	-0.302974	2.986414
C	-3.602075	1.566852	1.777485
H	-3.382152	-0.082831	3.133726
C	-1.658326	3.736360	-1.444796
C	-0.527420	4.694888	0.597775
H	0.074108	2.943591	-0.494434
H	-3.547332	3.193356	0.378188
C	4.907726	2.633041	-2.166418
C	2.681159	1.591704	-2.793139
H	3.235061	2.355482	-0.873074
C	5.443000	-1.604274	-1.286492
H	5.619086	0.058102	-2.633598
C	2.537360	-3.395689	1.409124
C	4.041273	-2.020486	2.928292
H	2.255090	-1.356068	1.961089
H	5.048531	-3.129174	0.172285
C	-7.085358	-1.891465	-0.665163
H	-7.514683	0.048105	0.174118
H	-6.375711	-3.693736	-1.615524
H	-0.850543	0.454148	5.038840
H	-0.361633	-1.250329	5.112706
H	-2.046633	-0.829264	4.788863
H	-0.346215	-2.680631	2.996114
H	-1.986512	-2.198116	2.522721
H	-0.630352	-1.929583	1.412993
H	-4.680795	1.608977	1.894404
H	-1.050859	4.371504	-2.099733
H	-2.579821	4.287508	-1.223620
H	-1.908627	2.828263	-1.997946
H	0.018949	5.394742	-0.045476
H	-1.443195	5.192629	0.939067
H	0.090734	4.493935	1.478756
H	4.561751	3.633398	-2.450475
H	5.672870	2.745390	-1.389462
H	5.385461	2.193401	-3.049388
H	3.109235	1.010352	-3.619018
H	2.359879	2.561703	-3.192523
H	1.791619	1.066051	-2.434094
H	6.202957	-2.189316	-1.797127
H	3.336758	-4.124585	1.232017
H	1.952161	-3.753303	2.264402
H	1.887958	-3.379558	0.527042
H	4.918586	-2.650788	2.740516

H	3.520239	-2.420878	3.806218
H	4.399156	-1.014123	3.175150
H	-8.021281	-2.334533	-0.335800

15

Ni	0.456348	0.830194	-0.086369
C	-0.947994	0.485157	1.135438
C	1.153092	-0.922495	0.125243
C	-2.351996	0.473924	0.507739
O	-0.836109	0.344020	2.349329
N	0.575711	-2.147119	-0.036317
N	2.431435	-1.211177	0.503534
C	-3.509530	0.784494	1.472332
H	-2.376372	1.153009	-0.350277
H	-2.498937	-0.522414	0.082869
C	1.472452	-3.178616	0.245297
C	-0.769716	-2.367935	-0.483568
C	2.636627	-2.587031	0.598330
C	3.503176	-0.258062	0.617588
C	-4.833324	0.690367	0.756468
H	-3.377219	1.786463	1.898589
H	-3.475074	0.081962	2.309421
H	1.187783	-4.215133	0.168043
C	-1.679607	-2.972835	0.400367
C	-1.119175	-1.970333	-1.792077
H	3.590637	-2.996873	0.888170
C	3.522015	0.626839	1.710492
C	4.500524	-0.271353	-0.372956
C	-5.542997	-0.517650	0.719031
C	-5.349862	1.786730	0.052102
C	-1.304483	-3.382501	1.818760
C	-2.984141	-3.189862	-0.062392
C	-0.095151	-1.362874	-2.744389
C	-2.440327	-2.193143	-2.193162
C	2.436509	0.589137	2.775598
C	4.600956	1.513554	1.800751
C	4.458817	-1.201236	-1.580660
C	5.556789	0.637153	-0.234959
C	-6.731685	-0.630191	-0.002714
H	-5.152381	-1.376424	1.258582
C	-6.538194	1.679728	-0.671665
H	-4.814898	2.734092	0.074253
C	-2.227072	-2.743024	2.868380
C	-1.290373	-4.914902	1.954267

H	-0.295488	-3.019202	2.026957
C	-3.364398	-2.796807	-1.340857
H	-3.710929	-3.655231	0.596141
C	-0.716454	-0.484218	-3.835216
C	0.774155	-2.471066	-3.366981
H	0.566513	-0.704902	-2.178257
H	-2.754733	-1.890392	-3.185460
C	2.181993	1.949662	3.434488
C	2.758264	-0.480636	3.833844
H	1.494873	0.305822	2.303822
C	5.611218	1.517843	0.840597
H	4.652794	2.207562	2.632879
C	4.221906	-0.420728	-2.883047
C	5.722047	-2.073069	-1.661357
H	3.607983	-1.875680	-1.469523
H	6.338503	0.659842	-0.988193
C	-7.233716	0.469106	-0.702665
H	-7.268810	-1.575279	-0.015542
H	-6.923316	2.543043	-1.208166
H	-3.274297	-3.034262	2.726124
H	-1.928635	-3.067632	3.872529
H	-2.152552	-1.653230	2.832518
H	-2.289743	-5.336008	1.792030
H	-0.615099	-5.379212	1.226139
H	-0.961947	-5.208005	2.958287
H	-4.386626	-2.950268	-1.674132
H	-1.383677	0.271399	-3.405563
H	-1.285056	-1.063454	-4.573144
H	0.082117	0.040642	-4.370345
H	1.540920	-2.031850	-4.015382
H	0.165602	-3.155512	-3.971770
H	1.283168	-3.063374	-2.599019
H	2.020959	2.730547	2.681639
H	3.009507	2.267942	4.080087
H	1.282143	1.888576	4.055240
H	1.953291	-0.535501	4.576226
H	3.694272	-0.249817	4.358246
H	2.862687	-1.471900	3.378576
H	6.439578	2.215676	0.929210
H	5.032962	0.291325	-3.078628
H	4.177833	-1.114741	-3.732012
H	3.275002	0.122478	-2.811513
H	5.640137	-2.782970	-2.492966
H	5.876126	-2.646374	-0.739234

H	6.621363	-1.469308	-1.829824
H	-8.160458	0.384689	-1.263635
N	-0.069493	2.783416	-0.183617
C	-0.813972	3.483102	0.890048
C	-0.651972	3.101980	-1.513801
H	0.878806	3.163628	-0.181813
C	-0.919058	4.976341	0.595749
H	-0.309315	3.300636	1.842198
H	-1.815263	3.052267	0.944742
C	-0.767320	4.607839	-1.713367
H	-0.017149	2.626779	-2.264721
H	-1.647554	2.650380	-1.557464
O	-1.524590	5.212905	-0.668760
H	-1.544925	5.472195	1.343906
H	0.083958	5.438542	0.624902
H	-1.285864	4.834919	-2.649629
H	0.237934	5.061826	-1.756882
C	2.591096	2.287060	-1.444455
H	3.496670	2.139784	-2.059375
H	2.964332	2.508312	-0.423731
H	2.144610	3.236742	-1.828716
O	1.741602	1.197565	-1.514318

TS16

Ni	-0.445807	0.849448	0.057641
C	0.949274	0.561777	-1.128086
C	-1.094485	-0.955199	-0.011326
C	2.364490	0.486035	-0.559874
O	0.770901	0.493626	-2.336308
N	0.077619	2.721877	0.275165
N	-2.352024	-1.376240	-0.334883
N	-0.431690	-2.117880	0.269629
C	3.432264	1.171179	-1.431238
H	2.388997	0.867163	0.461205
H	2.608678	-0.575951	-0.489493
C	1.271262	3.159346	0.990891
C	-0.175071	3.597618	-0.869322
C	-2.464295	-2.764118	-0.270778
C	-3.494348	-0.535539	-0.574808
C	-1.257270	-3.231368	0.120137
C	0.939642	-2.224765	0.685052
C	4.804221	0.904732	-0.864234
H	3.350470	0.785985	-2.452184
H	3.238103	2.248921	-1.478065

C	1.128970	4.622088	1.411623
H	1.417750	2.531255	1.877626
H	2.172710	3.074967	0.363955
C	-0.267115	5.056119	-0.422958
H	-1.111905	3.301378	-1.354561
H	0.626774	3.504860	-1.620326
H	-3.390919	-3.267405	-0.494556
C	-4.493410	-0.497374	0.412794
C	-3.590930	0.162561	-1.794052
H	-0.900071	-4.231693	0.302824
C	1.844456	-2.879564	-0.168652
C	1.319700	-1.682342	1.931446
C	5.506542	-0.257608	-1.212477
C	5.369537	1.763679	0.087198
O	0.902916	5.458600	0.282135
H	2.045009	4.987627	1.888592
H	0.294705	4.722785	2.127635
H	-0.356690	5.728993	-1.282650
H	-1.153791	5.189710	0.221198
C	-4.357911	-1.213262	1.752318
C	-5.642536	0.255121	0.138190
C	-2.483119	0.084708	-2.834853
C	-4.755825	0.904982	-2.012771
C	1.456009	-3.402285	-1.546418
C	3.171645	-3.001867	0.264361
C	0.306121	-1.002457	2.846455
C	2.661448	-1.813345	2.301922
C	6.736302	-0.555714	-0.625459
H	5.078927	-0.933537	-1.949262
C	6.599565	1.470160	0.677907
H	4.838303	2.671307	0.365135
C	-5.508442	-2.207662	1.975941
C	-4.246028	-0.216529	2.918215
H	-3.429599	-1.787735	1.745377
C	-5.775102	0.945833	-1.061249
H	-6.433143	0.306824	0.880704
C	-2.447648	1.286365	-3.785033
C	-2.576531	-1.228672	-3.633038
H	-1.523256	0.083960	-2.314057
H	-4.870259	1.457379	-2.938676
C	1.601699	-4.931305	-1.617187
C	2.265483	-2.715668	-2.659973
H	0.406340	-3.159866	-1.727335
C	3.579186	-2.467849	1.480996

H	3.897138	-3.493190	-0.376304
C	0.936477	-0.055561	3.872739
C	-0.579841	-2.046806	3.550382
H	-0.346247	-0.386433	2.220545
H	2.999252	-1.395542	3.243059
C	7.287187	0.307358	0.324917
H	7.267411	-1.459760	-0.912555
H	7.022387	2.151506	1.411699
H	-5.353846	-2.763497	2.908148
H	-5.579347	-2.931994	1.156011
H	-6.475114	-1.696444	2.051735
H	-5.138465	0.416521	2.992898
H	-4.145992	-0.760326	3.865622
H	-3.370458	0.427550	2.793929
H	-6.672850	1.525960	-1.256706
H	-2.434955	2.234016	-3.234762
H	-3.301746	1.301832	-4.473270
H	-1.536035	1.241041	-4.389338
H	-1.745194	-1.297022	-4.344245
H	-3.515849	-1.278580	-4.198153
H	-2.529823	-2.105499	-2.978582
H	1.009486	-5.429045	-0.840342
H	2.645562	-5.238693	-1.483311
H	1.266048	-5.303618	-2.592038
H	3.340604	-2.900879	-2.553169
H	1.958179	-3.105077	-3.637930
H	2.093419	-1.635545	-2.662124
H	4.618608	-2.545062	1.785992
H	1.597124	0.675322	3.393259
H	1.514933	-0.591005	4.635080
H	0.144315	0.497487	4.388986
H	-1.340112	-1.547257	4.161715
H	0.021057	-2.687100	4.208276
H	-1.096645	-2.692809	2.833582
H	8.245865	0.078133	0.782222
C	-3.036140	2.489398	0.938463
H	-3.916807	2.112539	1.464182
H	-3.205002	2.352821	-0.136835
H	-2.970425	3.570285	1.140608
O	-1.867088	1.825994	1.398528
H	-1.004784	2.494642	1.071925

17

Ni	-0.438600	0.857190	0.056279

C	0.966038	0.621159	-1.123149
C	-1.074633	-0.957103	-0.019017
C	2.375051	0.533253	-0.545035
O	0.791089	0.574650	-2.331584
N	0.043651	2.708090	0.269406
N	-2.329007	-1.390154	-0.344786
N	-0.397927	-2.116351	0.244753
C	3.452641	1.219613	-1.403563
H	2.393328	0.907518	0.478433
H	2.612676	-0.530464	-0.481242
C	1.221106	3.163025	0.994882
C	-0.200257	3.591191	-0.867581
C	-2.423332	-2.780325	-0.299607
C	-3.486434	-0.566987	-0.575432
C	-1.209674	-3.237763	0.081596
C	0.975590	-2.214157	0.655617
C	4.818783	0.935675	-0.831151
H	3.374795	0.846223	-2.429136
H	3.265755	2.298919	-1.438786
C	1.031054	4.611238	1.449301
H	1.388535	2.524868	1.871299
H	2.133815	3.127995	0.374116
C	-0.356414	5.036904	-0.396408
H	-1.110419	3.277958	-1.391770
H	0.627121	3.550932	-1.599249
H	-3.343696	-3.292428	-0.529111
C	-4.503584	-0.588477	0.394767
C	-3.585220	0.166238	-1.774165
H	-0.839782	-4.236169	0.248972
C	1.885566	-2.846178	-0.209800
C	1.352962	-1.687307	1.909467
C	5.525772	-0.214694	-1.207979
C	5.372611	1.764609	0.153257
O	0.784173	5.468113	0.339767
H	1.932290	4.995577	1.940242
H	0.189732	4.666851	2.162311
H	-0.452934	5.724541	-1.243999
H	-1.260222	5.123831	0.232116
C	-4.360744	-1.316189	1.727046
C	-5.675180	0.128699	0.118595
C	-2.462721	0.149186	-2.802025
C	-4.768562	0.880041	-1.989784
C	1.500446	-3.349547	-1.595638
C	3.214721	-2.961953	0.218996

C	0.334810	-1.029948	2.835625
C	2.696509	-1.810826	2.275769
C	6.749387	-0.530597	-0.617115
H	5.106954	-0.866950	-1.970686
C	6.595942	1.452880	0.748257
H	4.837262	2.662604	0.453587
C	-5.479558	-2.349804	1.930478
C	-4.296509	-0.322983	2.900152
H	-3.413340	-1.858601	1.726547
C	-5.808427	0.853227	-1.060720
H	-6.480947	0.130002	0.846521
C	-2.460547	1.366317	-3.732698
C	-2.496150	-1.152734	-3.623505
H	-1.509895	0.174114	-2.268939
H	-4.883492	1.458461	-2.899545
C	1.665846	-4.874961	-1.694703
C	2.298347	-2.632460	-2.698317
H	0.447283	-3.117721	-1.769805
C	3.619148	-2.443465	1.443332
H	3.944041	-3.435373	-0.430728
C	0.957843	-0.090390	3.873043
C	-0.540737	-2.091551	3.526449
H	-0.323558	-0.412554	2.217112
H	3.032058	-1.403895	3.222438
C	7.288575	0.302103	0.366372
H	7.284505	-1.424762	-0.926834
H	7.009978	2.110726	1.508046
H	-5.316336	-2.907709	2.859933
H	-5.517477	-3.069487	1.104347
H	-6.463564	-1.871968	2.000006
H	-5.212219	0.276569	2.969242
H	-4.185117	-0.867637	3.845762
H	-3.445036	0.355090	2.789084
H	-6.722239	1.407431	-1.256935
H	-2.492572	2.305635	-3.169208
H	-3.304545	1.359224	-4.433493
H	-1.539081	1.365214	-4.323524
H	-1.653050	-1.176619	-4.323501
H	-3.425179	-1.227254	-4.202689
H	-2.425449	-2.038783	-2.984034
H	1.082624	-5.394520	-0.925390
H	2.714019	-5.171148	-1.569628
H	1.331987	-5.233746	-2.675219
H	3.375928	-2.805996	-2.597065

H	1.993680	-3.007864	-3.682556
H	2.113252	-1.554635	-2.680653
H	4.659866	-2.514701	1.745181
H	1.609334	0.653920	3.401806
H	1.543964	-0.630187	4.626353
H	0.161491	0.446879	4.399610
H	-1.307449	-1.607394	4.142244
H	0.066309	-2.732495	4.177984
H	-1.049183	-2.735205	2.801623
H	8.242283	0.059114	0.826976
C	-3.154448	2.508902	0.960884
H	-4.045245	1.935980	1.214244
H	-3.248560	3.505877	1.415385
H	-3.121057	2.624057	-0.129881
O	-2.006290	1.832062	1.465214
H	-1.204895	2.435973	1.204526

18

Ni	-0.737558	0.721337	-0.168187
N	-1.293624	2.432695	-0.377006
C	0.585960	0.747335	-1.437313
C	-0.510474	3.630989	-0.138407
C	-2.237983	2.672017	-1.461748
C	-0.756373	-1.093952	0.403235
C	1.965303	1.108889	-0.887364
O	0.405528	0.522032	-2.621184
C	-1.431133	4.813396	0.177569
H	0.172185	3.472400	0.705314
H	0.102286	3.904050	-1.020911
C	-3.137479	3.861879	-1.123753
H	-2.859448	1.783448	-1.617074
H	-1.718039	2.891728	-2.413849
N	-1.952091	-1.755210	0.442181
N	0.116845	-1.983211	0.960288
C	2.991839	1.538025	-1.950442
H	1.852937	1.873643	-0.112773
H	2.326339	0.215946	-0.370821
O	-2.368481	5.034363	-0.869472
H	-0.860850	5.743222	0.282814
H	-1.964185	4.617533	1.124116
H	-3.803254	4.105821	-1.959001
H	-3.752318	3.617608	-0.240928
C	-1.832364	-3.014402	1.017428
C	-3.173953	-1.137003	0.001054

C	-0.523023	-3.159579	1.345324
C	1.514056	-1.712404	1.154775
C	4.389151	1.512851	-1.380492
H	2.919325	0.860223	-2.805700
H	2.736508	2.540249	-2.316517
H	-2.677270	-3.675052	1.129840
C	-3.600265	-1.350057	-1.320444
C	-3.862164	-0.303571	0.902703
H	0.013970	-3.972372	1.807650
C	1.891906	-0.857685	2.205726
C	2.441712	-2.274427	0.259645
C	4.803052	2.473065	-0.446160
C	5.279671	0.484096	-1.712561
C	-2.838092	-2.244444	-2.285696
C	-4.767018	-0.696392	-1.734471
C	-3.388898	-0.095508	2.334343
C	-5.025118	0.322384	0.441726
C	0.881859	-0.270764	3.181401
C	3.249310	-0.535191	2.318443
C	2.025257	-3.183770	-0.887527
C	3.788886	-1.934849	0.428173
C	6.063946	2.401817	0.145634
H	4.127260	3.282983	-0.180385
C	6.545046	0.409676	-1.127322
H	4.973273	-0.266480	-2.436893
C	-2.305157	-1.446487	-3.486760
C	-3.706291	-3.430204	-2.738991
H	-1.972704	-2.654075	-1.757620
C	-5.472357	0.130888	-0.863949
H	-5.121035	-0.834059	-2.751591
C	-4.292639	-0.858743	3.317588
C	-3.292711	1.392947	2.701769
H	-2.382100	-0.512839	2.421464
H	-5.581774	0.971734	1.110015
C	0.696321	1.236121	2.940767
C	1.266501	-0.559533	4.641043
H	-0.083963	-0.750493	2.999219
C	4.188847	-1.061623	1.436272
H	3.572548	0.144104	3.100994
C	2.683900	-4.567719	-0.765327
C	2.333886	-2.540967	-2.250233
H	0.943059	-3.329005	-0.836153
H	4.528478	-2.333801	-0.258393
C	6.940763	1.366927	-0.191315

H	6.365396	3.156941	0.867003
H	7.221700	-0.395096	-1.403459
H	-1.760250	-2.112424	-4.167305
H	-3.123539	-0.988309	-4.055112
H	-1.618145	-0.656977	-3.167384
H	-4.581782	-3.092467	-3.305812
H	-4.067082	-4.012802	-1.883014
H	-3.128250	-4.099352	-3.387100
H	-6.374393	0.631469	-1.204979
H	-3.928505	-0.740462	4.345184
H	-4.318920	-1.930188	3.087582
H	-5.322244	-0.483438	3.279590
H	-4.278498	1.872623	2.714485
H	-2.861470	1.504046	3.703954
H	-2.661338	1.927697	1.985371
H	1.638959	1.779097	3.079987
H	0.339846	1.418855	1.920195
H	-0.041799	1.650286	3.637527
H	1.399163	-1.633674	4.813916
H	2.198001	-0.056407	4.924080
H	0.480215	-0.200496	5.315409
H	5.232783	-0.776661	1.520933
H	2.330696	-5.229794	-1.564478
H	2.450533	-5.039827	0.196081
H	3.775013	-4.500316	-0.846622
H	2.023477	-3.209874	-3.061904
H	3.407218	-2.351137	-2.367326
H	1.802061	-1.593662	-2.375064
H	7.925193	1.313173	0.265613

TS19

Ni	-0.706232	0.717010	-0.076578
N	-0.504440	2.457763	-0.704414
C	0.633894	0.886267	-1.365655
C	0.197589	3.555503	-0.069763
C	-1.314798	2.929134	-1.812431
C	-0.964211	-1.063649	0.458465
C	2.032019	0.994298	-0.778370
O	0.443672	0.633209	-2.548536
C	-0.815102	4.603451	0.404060
H	0.770939	3.197083	0.791252
H	0.899401	4.048170	-0.769593
C	-2.305061	3.979397	-1.311480
H	-1.853976	2.090050	-2.262017

H	-0.686680	3.385163	-2.600315
N	-0.161024	-2.062311	0.933500
N	-2.217048	-1.608444	0.522711
C	3.056553	1.663363	-1.714312
H	2.012395	1.493436	0.191990
H	2.350892	-0.034500	-0.586552
O	-1.628347	5.059353	-0.670142
H	-0.312220	5.490955	0.805417
H	-1.445633	4.163372	1.195565
H	-2.870401	4.424497	-2.137993
H	-3.012616	3.506532	-0.609650
C	-0.896546	-3.192229	1.283833
C	1.269048	-1.956261	1.020206
C	-2.197983	-2.904864	1.023511
C	-3.374468	-0.877198	0.084171
C	4.450786	1.474704	-1.171029
H	2.964835	1.214314	-2.707703
H	2.817581	2.728745	-1.815452
H	-0.424148	-4.077483	1.678838
C	1.829781	-1.239662	2.091622
C	2.047887	-2.537487	0.003623
H	-3.098003	-3.487238	1.142085
C	-3.783239	-1.010394	-1.253983
C	-4.005595	-0.009350	0.992820
C	4.933604	2.286597	-0.135382
C	5.259755	0.424312	-1.623994
C	0.975924	-0.614753	3.185404
C	3.220846	-1.088146	2.110555
C	1.431416	-3.288748	-1.167907
C	3.436457	-2.378060	0.082371
C	-3.074739	-1.937104	-2.230055
C	-4.869338	-0.235191	-1.675951
C	-3.517202	0.154959	2.424223
C	-5.086277	0.744738	0.522219
C	6.184993	2.053578	0.435550
H	4.318346	3.107841	0.226127
C	6.514759	0.189205	-1.060245
H	4.895717	-0.216224	-2.423715
C	1.456224	-1.017351	4.588434
C	0.927107	0.913985	3.029830
H	-0.046340	-0.986867	3.069668
C	4.018126	-1.649347	1.116814
H	3.684089	-0.516643	2.908568
C	1.786655	-2.630950	-2.511619

C	1.841914	-4.770540	-1.147146
H	0.343663	-3.245337	-1.067016
H	4.066009	-2.804382	-0.692379
C	-2.398649	-1.144924	-3.361214
C	-4.037861	-2.998851	-2.786249
H	-2.285912	-2.464666	-1.686871
C	-5.513648	0.634651	-0.799232
H	-5.207234	-0.308445	-2.705236
C	-4.642439	-0.069967	3.445947
C	-2.859517	1.532208	2.614127
H	-2.750166	-0.602399	2.609399
H	-5.591149	1.431390	1.194715
C	6.981180	1.001781	-0.024488
H	6.541531	2.695853	1.236753
H	7.129320	-0.627306	-1.430961
H	1.495303	-2.106989	4.700004
H	0.772584	-0.621420	5.348608
H	2.454705	-0.621582	4.805846
H	1.930798	1.351281	3.093751
H	0.495383	1.180328	2.057457
H	0.309101	1.363632	3.816102
H	5.093127	-1.501102	1.137279
H	2.868832	-2.638277	-2.688419
H	1.313791	-3.181533	-3.333999
H	1.436509	-1.595447	-2.554315
H	1.354165	-5.314211	-1.964796
H	2.925465	-4.884850	-1.269646
H	1.561147	-5.252359	-0.203233
H	-3.135531	-0.578185	-3.943645
H	-1.890205	-1.832633	-4.048525
H	-1.650045	-0.447026	-2.973873
H	-4.837610	-2.544547	-3.382779
H	-4.507774	-3.574844	-1.980217
H	-3.497589	-3.698653	-3.434484
H	-6.352082	1.231365	-1.147824
H	-5.107386	-1.054003	3.316571
H	-4.244691	-0.012514	4.465969
H	-5.429224	0.687589	3.355783
H	-2.460467	1.632681	3.630513
H	-2.035481	1.675492	1.904798
H	-3.581033	2.341332	2.450018
H	7.958385	0.822100	0.415496

20

C	-0.829263	-1.282888	0.366791
N	-0.083012	-2.297537	0.914547
N	-2.035477	-1.410434	1.003456
C	-0.808434	-3.032927	1.846529
C	1.286856	-2.530927	0.549586
Ni	-0.354328	-0.189989	-1.067013
C	-2.042019	-2.469345	1.905800
C	-3.138495	-0.520902	0.776193
H	-0.383713	-3.877373	2.366159
C	1.559419	-3.145504	-0.683522
C	2.300768	-2.097921	1.427726
H	-2.913357	-2.715963	2.491536
C	-3.329063	0.547227	1.669242
C	-3.966520	-0.739090	-0.338693
C	0.457760	-3.648336	-1.605048
C	2.902872	-3.293617	-1.050908
C	1.963715	-1.420080	2.751061
C	3.625788	-2.272176	1.016474
C	-2.386103	0.804425	2.835724
C	-4.408341	1.405583	1.430400
C	-3.720971	-1.880342	-1.314467
C	-5.019659	0.158862	-0.546787
C	0.421655	-2.846989	-2.915476
C	0.599188	-5.154849	-1.876316
H	-0.501195	-3.496208	-1.102426
C	3.924794	-2.857270	-0.213739
H	3.146948	-3.757096	-2.001951
C	3.023663	-1.645672	3.837046
C	1.717654	0.085083	2.541843
H	1.027866	-1.852726	3.119289
H	4.434118	-1.938628	1.657249
C	-3.121545	0.719288	4.182648
C	-1.666831	2.154384	2.675858
H	-1.617719	0.027405	2.828193
C	-5.244284	1.216389	0.332285
H	-4.584210	2.239743	2.103103
C	-4.978462	-2.736395	-1.528972
C	-3.176588	-1.343258	-2.648463
H	-2.952475	-2.529900	-0.886077
H	-5.671156	0.028223	-1.405596
H	1.352728	-2.967095	-3.482083
H	-0.407310	-3.182523	-3.549484
H	0.292783	-1.774594	-2.716471
H	-0.234320	-5.508943	-2.494503

H	1.528593	-5.384512	-2.409875
H	0.598515	-5.728969	-0.942537
H	4.961393	-2.979026	-0.516144
H	3.958567	-1.120956	3.610136
H	3.253063	-2.709444	3.967885
H	2.659711	-1.256216	4.794572
H	2.631320	0.577087	2.190212
H	1.417630	0.556431	3.485837
H	0.933849	0.261199	1.801216
H	-3.903366	1.483995	4.260622
H	-2.420511	0.874064	5.011395
H	-3.597620	-0.258834	4.318591
H	-0.930904	2.287623	3.478141
H	-2.374677	2.990187	2.734135
H	-1.148570	2.214976	1.714008
H	-6.070253	1.900045	0.156550
H	-5.363947	-3.124500	-0.579132
H	-4.748019	-3.590172	-2.177041
H	-5.781956	-2.166700	-2.009517
H	-2.961592	-2.168227	-3.337921
H	-3.900966	-0.676570	-3.131673
H	-2.250630	-0.774495	-2.490201
C	0.372426	1.637068	-1.210912
C	1.764218	1.695707	-0.591020
N	-0.517994	2.695893	-0.861753
O	0.229194	1.008193	-2.352466
C	2.854143	2.247998	-1.535103
H	1.747021	2.259250	0.347313
H	2.048015	0.673748	-0.330678
C	-1.831207	2.620414	-1.502776
C	0.022923	4.060463	-0.915022
C	4.222152	2.076404	-0.920645
H	2.671652	3.303775	-1.764392
H	2.785242	1.695475	-2.478891
C	-2.766755	3.656982	-0.896223
H	-2.231805	1.615185	-1.338200
H	-1.755081	2.780271	-2.590116
C	-0.983499	5.043917	-0.328522
H	0.949415	4.115168	-0.338370
H	0.248687	4.355516	-1.955445
C	4.804308	0.801869	-0.846173
C	4.910213	3.156021	-0.354168
O	-2.233667	4.975394	-0.996934
H	-3.721036	3.664857	-1.431427

H	-2.960612	3.414004	0.158110
H	-0.620709	6.070405	-0.443404
H	-1.116056	4.835934	0.746503
C	6.038757	0.614309	-0.226126
H	4.278270	-0.048388	-1.272804
C	6.147807	2.973279	0.267189
H	4.471066	4.150191	-0.401710
C	6.716521	1.700720	0.334058
H	6.475493	-0.380211	-0.182658
H	6.667030	3.825660	0.697758
H	7.679769	1.555976	0.815794

21

C	-0.229492	0.290009	-0.112767
C	-1.407259	1.198359	0.268269
C	1.111508	1.011816	0.002565
C	-2.724732	0.467076	0.155979
H	-1.402230	2.079473	-0.381019
H	-1.258292	1.564559	1.288669
H	-0.364496	-0.081276	-1.136594
H	-0.227089	-0.597950	0.532677
N	2.232310	0.307645	-0.353733
O	1.177545	2.175048	0.399588
C	-3.442987	0.462226	-1.047027
C	-3.235421	-0.260359	1.239275
C	2.275766	-1.072387	-0.825575
C	3.549472	0.933969	-0.264737
C	-4.638075	-0.248476	-1.166196
H	-3.062460	1.026604	-1.895595
C	-4.429836	-0.972954	1.125921
H	-2.692458	-0.261500	2.181966
C	3.255551	-1.872783	0.034613
H	1.287235	-1.528256	-0.783342
H	2.616331	-1.087637	-1.870918
C	4.474953	0.057359	0.573297
H	3.425443	1.921507	0.179859
H	3.966017	1.046273	-1.275662
C	-5.135522	-0.969707	-0.078991
H	-5.183280	-0.235152	-2.106310
H	-4.812229	-1.526282	1.979663
O	4.540474	-1.268576	0.055835
H	3.388793	-2.879289	-0.373386
H	2.856240	-1.956011	1.059392
H	5.496837	0.447480	0.557465

H	4.119478	0.037932	1.616489
H	-6.067718	-1.520661	-0.168354

22

C	2.72127400	0.86342500	0.34374400
C	3.11662800	-0.45963100	0.12842800
C	2.17088400	-1.42142300	-0.23474900
C	0.83043600	-1.06587900	-0.37445300
C	0.42827000	0.25891600	-0.15331400
C	1.38376100	1.22195900	0.19904500
H	3.45614500	1.61320500	0.62245800
H	4.16059300	-0.73965800	0.23891300
H	2.47891000	-2.44768500	-0.41344000
H	0.09838700	-1.80824400	-0.67284200
H	1.05665900	2.24433800	0.35571100
C	-0.99408100	0.69464900	-0.28278900
O	-1.32309500	1.87260500	-0.32438500
C	-2.30201400	-1.22117000	0.68815500
C	-3.25977800	-0.21149000	0.13475100
H	-2.40707000	-2.27398800	0.43644300
H	-4.06849300	-0.53632400	-0.51531200
H	-3.46212600	0.68145900	0.72210900
N	-1.92715100	-0.33894600	-0.41339800
H	-1.85971700	-1.01896900	1.66178500

23

C	-3.35622800	0.50798300	0.72656500
C	-3.68598900	-0.70106100	0.10810900
C	-2.72425500	-1.38520300	-0.63710800
C	-1.43064000	-0.87416600	-0.74766500
C	-1.09125300	0.33208700	-0.12146000
C	-2.07033200	1.02803200	0.60020100
H	-4.10439200	1.04821800	1.29988700
H	-4.69111100	-1.10331300	0.19969200
H	-2.98133600	-2.31491400	-1.13704100
H	-0.69336800	-1.40200400	-1.34361300
H	-1.80720300	1.97885700	1.05184800
C	0.25994100	0.97352200	-0.25828400
O	0.36077400	2.19698300	-0.38991900
C	2.68876600	0.81096900	-0.34016100
C	1.46350300	-1.20350800	0.27476900
C	2.86778800	-1.24690200	0.88846400
H	2.75913900	1.61721700	0.39972800
H	2.82626800	1.26166500	-1.32757000

H	0.66776500	-1.40844100	0.99347200
H	1.38147100	-1.93414400	-0.54090200
H	2.84381200	-0.82768100	1.90142200
H	3.26383200	-2.26480000	0.94828600
N	1.36428300	0.17257900	-0.24328500
C	3.67030300	-0.32947900	-0.04526600
H	3.92243400	-0.86341300	-0.96940600
H	4.60174200	0.03294000	0.39851700

24

C	3.71275700	-0.22657100	-0.72751700
C	3.94660200	-0.87801100	0.48625800
C	2.94039200	-0.92892700	1.45260900
C	1.69966500	-0.34308300	1.20175200
C	1.45483700	0.30117300	-0.01890800
C	2.47695500	0.36633400	-0.97426000
H	4.49551000	-0.17841200	-1.47931000
H	4.91180500	-1.33686100	0.68169900
H	3.12333800	-1.41753500	2.40547300
H	0.93920700	-0.36275600	1.97762700
H	2.28012400	0.88808600	-1.90463300
C	0.14794100	0.96234200	-0.35652500
O	0.07702900	1.85393300	-1.20405500
N	-0.94674600	0.50472100	0.31266000
C	-2.27040900	1.08252700	0.12117400
C	-2.54695400	2.24895000	1.07019400
H	-2.48016000	1.93107300	2.11733500
H	-1.81997200	3.05096200	0.90676900
H	-3.55123400	2.65437200	0.89858200
H	-2.33123900	1.41993400	-0.91665400
H	-3.00744600	0.28505700	0.26210500
H	-0.84864300	-0.27686200	0.94314800

25

C	4.16310700	-1.17330200	0.78875500
C	4.69202800	-0.99685200	-0.49239800
C	4.01689400	-0.20288900	-1.42162700
C	2.80462400	0.39656800	-1.07950900
C	2.26480400	0.21316400	0.20048900
C	2.96231400	-0.55883000	1.13827100
H	4.69095400	-1.78221300	1.51718000
H	5.63192300	-1.46986000	-0.76273700
H	4.43561900	-0.04673400	-2.41174800
H	2.28829600	1.02768300	-1.79659900

H	2.55256300	-0.66694600	2.13747100
C	0.99810500	0.88294500	0.64458600
O	0.91925700	1.41000100	1.75217800
N	-0.05135500	0.93434800	-0.24365000
C	-0.38884800	-0.07709500	-1.25245000
H	-0.88809400	0.42725500	-2.08629200
H	0.54022100	-0.49817400	-1.63732800
C	-1.27459000	-1.17816100	-0.70414100
C	-2.61523700	-1.27759000	-1.08712000
C	-0.76108900	-2.10181700	0.21703800
C	-3.43317900	-2.28194400	-0.56341700
H	-3.02140400	-0.56548800	-1.80191100
C	-1.57422500	-3.10426300	0.74157600
H	0.28043600	-2.03309100	0.52114100
C	-2.91393700	-3.19666500	0.35245700
H	-4.47325200	-2.34767300	-0.87089300
H	-1.16385500	-3.81612200	1.45256200
H	-3.54737700	-3.97859000	0.76195700
H	-0.85904400	1.38169000	0.18087400

26

C	4.24951400	-1.22431800	0.66970300
C	4.73439800	-0.97759700	-0.61745700
C	4.01117000	-0.16092700	-1.48868900
C	2.79643400	0.39174100	-1.08244900
C	2.29989100	0.13772700	0.20333700
C	3.04579100	-0.65759600	1.08246000
H	4.81457400	-1.85147700	1.35348800
H	5.67715700	-1.41312700	-0.93669600
H	4.39453700	0.05076500	-2.48290900
H	2.24587900	1.04238500	-1.75454400
H	2.66949200	-0.82106600	2.08701100
C	1.03836600	0.76473200	0.72047400
O	0.97698800	1.19574200	1.87231200
N	-0.02262400	0.88392400	-0.13775800
H	-0.81307100	1.34380500	0.30424100
C	-0.35263300	0.06613200	-1.29896500
C	-1.61386800	-0.79284600	-1.10892800
C	-0.66954700	0.88515700	-2.56669800
H	0.49404400	-0.59601100	-1.49102300
C	-1.94244900	-1.24442400	-2.53876300
H	-2.42040900	-0.16237000	-0.70781400
H	-1.45815400	-1.61978100	-0.40861200
C	-1.54635700	-0.04297900	-3.44622800

H	0.23882600	1.22811300	-3.07230500
H	-1.22805300	1.78161900	-2.27046400
H	-1.33589400	-2.12213700	-2.79234200
H	-2.99074400	-1.53470700	-2.65870700
H	-1.00563100	-0.38658900	-4.33374600
H	-2.43247600	0.49033500	-3.80477400

27

C	4.02435100	-1.33941200	0.76019200
C	4.47447400	-1.31285900	-0.56236800
C	3.85824300	-0.47114700	-1.48983700
C	2.78542200	0.33116700	-1.10172100
C	2.32102100	0.29906900	0.22115400
C	2.95998300	-0.53157100	1.15133300
H	4.50567400	-1.98806000	1.48644500
H	5.30816900	-1.93927100	-0.86674300
H	4.21794100	-0.43094200	-2.51390300
H	2.34275800	1.00799300	-1.82752500
H	2.60602800	-0.52988900	2.17655000
C	1.18390800	1.14286300	0.72002800
O	1.09407500	1.46186000	1.90318600
N	0.26787000	1.50079500	-0.24068600
H	0.38294400	1.07211500	-1.14903100
C	-0.88721100	2.29489200	-0.11001000
C	-1.70802200	2.41572200	-1.24333300
C	-1.23865200	2.96525200	1.07169000
C	-2.86195400	3.19175100	-1.19924800
H	-1.43501300	1.89603000	-2.15944500
C	-2.39834900	3.74020300	1.09855000
H	-0.61138100	2.86717400	1.94533700
C	-3.21574900	3.86127800	-0.02571400
H	-3.48462900	3.27229200	-2.08589200
H	-2.66199500	4.25508400	2.01843900
H	-4.11598500	4.46751500	0.01063300

28

C	-4.43318500	-0.98204300	0.61448300
C	-4.64700400	-1.73243000	-0.54475600
C	-3.56177100	-2.27062100	-1.24078900
C	-2.26241800	-2.04032300	-0.79128200
C	-2.04568300	-1.28385200	0.36771400
C	-3.13690600	-0.76916200	1.07752500
H	-5.27667200	-0.56626700	1.15786000
H	-5.65864600	-1.90229400	-0.90247000

H	-3.72789200	-2.86921600	-2.13176300
H	-1.41430500	-2.46132700	-1.32357000
H	-2.95438100	-0.19490700	1.97976200
C	-0.67283500	-1.06227300	0.91416700
O	-0.44000800	-1.13284900	2.10461400
N	0.32602700	-0.74035800	-0.04397400
C	1.66703800	-1.00976500	0.28082200
O	2.01030000	-1.92989800	0.99083800
O	2.48448500	-0.13200500	-0.33149300
C	3.95207300	-0.27962400	-0.25951300
C	4.37030000	-1.61983600	-0.86393400
H	5.46254500	-1.65945400	-0.93770400
H	4.03210100	-2.45664800	-0.25115600
H	3.95807700	-1.72926000	-1.87328400
C	4.41432500	-0.11891200	1.18795100
H	5.50940500	-0.11226700	1.22228400
H	4.05513800	0.83039100	1.60022300
H	4.04781300	-0.93490100	1.81187200
C	4.43968700	0.88298200	-1.12156600
H	4.08951400	1.83880000	-0.71825500
H	5.53410300	0.89736000	-1.14447700
H	4.07379600	0.78637600	-2.14924800
C	-0.03355000	0.19950000	-1.13384900
H	-0.77629100	-0.27311200	-1.77728200
H	0.86804500	0.34774100	-1.72521800
C	-0.56761800	1.51792300	-0.61684300
C	-1.83612700	1.96328800	-1.00029100
C	0.18958800	2.29676000	0.26815000
C	-2.33825800	3.17491200	-0.52099400
H	-2.43839200	1.35168000	-1.66709100
C	-0.31171900	3.50397700	0.75118300
H	1.16999700	1.94893900	0.57914500
C	-1.57679900	3.94776400	0.35581200
H	-3.32691000	3.50769100	-0.82499400
H	0.28386600	4.09939200	1.43776200
H	-1.96739200	4.88823500	0.73452500

29

C	-3.35817500	-0.77091200	0.43828300
C	-3.72576500	0.36470300	-0.28844700
C	-2.74404600	1.18213700	-0.85288700
C	-1.39564800	0.87738200	-0.67562100
C	-1.02218900	-0.25775700	0.05770400
C	-2.01215900	-1.08904100	0.59900100

H	-4.12098700	-1.41034700	0.87274700
H	-4.77613600	0.60876800	-0.41982100
H	-3.02817900	2.05544800	-1.43266000
H	-0.63397100	1.50720400	-1.12408200
H	-1.71124200	-1.97887900	1.14197300
C	0.40448600	-0.68612300	0.18861800
O	0.70367700	-1.87109700	0.17813500
N	1.37488300	0.32996800	0.28668100
C	1.09569800	1.58197600	1.00592500
H	1.83195800	1.70216900	1.80318000
H	1.16883100	2.44583800	0.33950300
H	0.09876600	1.53688000	1.43863500
C	2.71248700	0.16287300	-0.11910600
O	3.51930300	1.04204600	0.14287600
C	3.09971800	-1.06025100	-0.91643700
H	3.18340500	-1.93233900	-0.26324500
H	2.36469800	-1.30647900	-1.68693300
H	4.06880900	-0.85182800	-1.37436200

30

C	3.63049200	0.35831800	-0.33969700
C	3.72418000	-0.41899000	0.81894200
C	2.56695000	-0.86713800	1.45998700
C	1.31465000	-0.54484500	0.94321900
C	1.21574600	0.23392600	-0.21936100
C	2.38218300	0.68781700	-0.85546800
H	4.53100700	0.70504200	-0.83758600
H	4.69983000	-0.67478700	1.22222500
H	2.64057100	-1.46794400	2.36137600
H	0.41711300	-0.89004100	1.44452000
H	2.28855000	1.29097700	-1.75191600
C	-0.08677600	0.63553700	-0.79036800
O	-0.22656800	1.45078600	-1.67374300
N	-1.27988700	0.03034300	-0.17281200
C	-1.51337400	-1.29738100	-0.60116800
O	-0.73658300	-1.77077300	-1.41465000
C	-2.67486500	-2.06797400	-0.03683600
H	-2.66003800	-2.07444000	1.05616000
H	-3.62379300	-1.61388800	-0.33377700
H	-2.60754500	-3.08592400	-0.42590000
C	-2.13266600	0.82418900	0.62152300
O	-3.18979400	0.40549400	1.05592300
C	-1.63499600	2.22341700	0.90924300
H	-0.58864100	2.22743800	1.23273100

H	-1.70714800	2.84321300	0.01006800
H	-2.26329500	2.64947200	1.69248900

31

C	3.47713300	-0.78532200	0.59081100
C	3.19934400	-1.81139000	-0.31533200
C	2.06867300	-1.73154800	-1.13118500
C	1.20568200	-0.64174900	-1.02886200
C	1.47473300	0.38574900	-0.11476600
C	2.62599800	0.31399200	0.68013100
H	4.35995500	-0.83862000	1.22166100
H	3.86505700	-2.66678100	-0.39044700
H	1.85711800	-2.51951700	-1.84840400
H	0.32920700	-0.58749700	-1.66441700
H	2.84183600	1.13035200	1.36146800
C	0.63598700	1.62502400	-0.00116900
O	1.16089400	2.71748700	0.21062100
N	-0.72802500	1.52108300	-0.19563800
C	-1.46653300	0.31976700	0.03534300
C	-1.26892700	-0.42730600	1.20153800
C	-2.41356700	-0.10140900	-0.90578800
C	-1.99175200	-1.60181800	1.40813000
H	-0.54501400	-0.08971700	1.93579200
C	-3.14672900	-1.26630900	-0.68548000
H	-2.55742300	0.47789400	-1.81312900
C	-2.93358900	-2.02416600	0.46851400
H	-1.82473300	-2.18070600	2.31195600
H	-3.87647100	-1.58860600	-1.42282300
H	-3.49963900	-2.93605100	0.63463900
C	-1.47745500	2.78014600	-0.21151800
H	-2.46665600	2.59897300	-0.63421600
H	-1.59211600	3.18575800	0.80168300
H	-0.94505600	3.51366600	-0.81831700

32

C	-5.50556700	0.06444200	-1.22296800
C	-5.92252500	0.12370000	0.10978000
C	-4.99733800	0.38688200	1.12202100
C	-3.65336500	0.57468300	0.80821900
C	-3.23122100	0.50882900	-0.52629100
C	-4.16658900	0.26969200	-1.54192100
H	-6.22519900	-0.13692400	-2.01111800
H	-6.96890600	-0.03049200	0.35806400
H	-5.32177600	0.44526600	2.15669000

H	-2.93724700	0.78561700	1.59511600
H	-3.82858700	0.24323800	-2.57249000
C	-1.82951000	0.80097200	-0.92409100
O	-1.55615500	1.36657100	-1.96969100
N	-0.80212100	0.50180000	0.01099500
S	-0.74986900	-0.95922600	0.95201300
O	-0.65833800	-0.57863400	2.36459600
O	-1.83067600	-1.82450400	0.47930800
C	0.81053500	-1.64566000	0.44396000
C	0.86931900	-2.35333400	-0.75737200
C	1.94916800	-1.42436800	1.21805100
C	2.09958200	-2.84104300	-1.18741500
H	-0.03004100	-2.51667700	-1.34144100
C	3.16971600	-1.91949700	0.76948700
H	1.87670500	-0.86405500	2.14253400
C	3.26499800	-2.63053600	-0.43547100
H	2.15822600	-3.39348800	-2.12107000
H	4.06462400	-1.74686400	1.36092300
C	4.58894000	-3.18177400	-0.89712300
H	4.78772700	-4.15609100	-0.43103400
H	4.60630400	-3.32798400	-1.98169000
H	5.41577600	-2.51730900	-0.62521200
C	0.43803600	1.23293800	-0.04098300
C	1.28490400	1.13651500	-1.14856900
C	0.80022600	1.99803800	1.06899400
C	2.50012200	1.81757100	-1.14134500
H	0.98964400	0.53071400	-1.99570900
C	2.02548800	2.66601100	1.07452500
H	0.12874500	2.05048600	1.91878600
C	2.87455800	2.57725800	-0.02970300
H	3.15979800	1.74736400	-2.00121500
H	2.31165200	3.25781000	1.93904400
H	3.82681800	3.09999500	-0.02578200

33

C	3.83328700	0.00509300	0.55571300
C	3.89331700	-0.75141200	-0.61770400
C	2.79265600	-0.79850900	-1.47625100
C	1.62578200	-0.10870300	-1.15455000
C	1.55877300	0.64533700	0.02454300
C	2.67507900	0.71122700	0.86915600
H	4.69052200	0.04788900	1.22130500
H	4.79841800	-1.29888900	-0.86542200
H	2.84299700	-1.37281800	-2.39669600

H	0.77449400	-0.14078500	-1.82445500
H	2.61804400	1.31815700	1.76676500
C	0.36179400	1.46256700	0.38261800
O	0.45571100	2.50803400	0.99413200
N	-0.90825000	0.93717600	-0.01747300
C	-1.14542600	-0.46945300	0.13614400
C	-0.89741100	-1.08275800	1.36763800
C	-1.61076100	-1.22847500	-0.94216000
C	-1.10613400	-2.45381400	1.51501000
H	-0.54354200	-0.48442600	2.20161900
C	-1.83687200	-2.59437800	-0.78408100
H	-1.77970600	-0.74686900	-1.90063700
C	-1.58043400	-3.21131200	0.44282900
H	-0.90542900	-2.92707400	2.47165700
H	-2.20036200	-3.17928600	-1.62390800
H	-1.74793800	-4.27772200	0.56132000
C	-1.96674000	1.83127400	-0.32284500
O	-1.74324000	2.96138400	-0.70528900
C	-3.37387600	1.29335000	-0.15484000
H	-3.65652100	0.64921000	-0.99364200
H	-3.48132500	0.70657000	0.76191100
H	-4.05165800	2.14874100	-0.13486900

34

C	-0.58154700	-1.32819200	1.09632300
O	-0.23647900	-1.68431100	2.20543100
N	0.35093900	-0.88733100	0.12704400
C	1.72791400	-1.11391500	0.34947300
O	2.17408900	-2.05694500	0.96170700
O	2.44486300	-0.13530200	-0.23494900
C	3.92100400	-0.17055800	-0.24562200
C	4.40910500	-1.42802000	-0.96412000
H	5.49579200	-1.37383900	-1.09140900
H	4.16963900	-2.32945500	-0.39838000
H	3.95503800	-1.50076900	-1.95872600
C	4.44471700	-0.07133500	1.18646600
H	5.53583200	0.02557000	1.16940300
H	4.03199800	0.81420300	1.68219000
H	4.18069400	-0.95594200	1.76769000
C	4.26937600	1.08417600	-1.04352800
H	3.85923600	1.97669000	-0.55997200
H	5.35644200	1.19434000	-1.11149000
H	3.86340900	1.02430200	-2.05893900
C	-0.07783300	-0.05832400	-1.01444900

H	-0.85957600	-0.58707700	-1.56483700
H	0.77804200	0.01667300	-1.68439400
C	-0.56106300	1.32594300	-0.63085200
C	-1.61657000	1.91116200	-1.33911500
C	0.03865900	2.04293300	0.41074400
C	-2.06254700	3.19463200	-1.02007600
H	-2.09498700	1.35588300	-2.14311700
C	-0.40974500	3.32341800	0.73438800
H	0.85558200	1.59624000	0.96754900
C	-1.45988100	3.90409500	0.01984300
H	-2.88540800	3.63459300	-1.57667500
H	0.06194000	3.86830000	1.54749700
H	-1.80913700	4.90050400	0.27539200
C	-2.03995500	-1.37029100	0.65431000
C	-2.28561500	-2.61500700	-0.22930600
C	-2.98945700	-1.41556900	1.85784200
H	-2.28058200	-0.47731900	0.06852600
C	-3.77016400	-2.71092300	-0.58296400
H	-1.98597900	-3.51232300	0.32517600
H	-1.68741300	-2.58220300	-1.14769200
C	-4.42899800	-1.58322100	1.37554500
H	-2.71710300	-2.25281800	2.50712500
H	-2.90009900	-0.49753200	2.45037300
H	-3.97989200	-3.63073000	-1.13793100
H	-4.05530600	-1.85546800	-1.22171400
H	-5.11394500	-1.70090700	2.22057800
H	-4.74428200	-0.69122900	0.80549100
O	-4.59161300	-2.74824700	0.57290700

35

C	-0.88324100	-1.41583400	0.98798100
O	-0.49240600	-2.02616700	1.96357100
N	0.00570100	-0.93710000	-0.00190000
C	1.35656100	-1.35580800	0.02846300
O	1.72635700	-2.44274700	0.40822200
O	2.14074500	-0.36689000	-0.43946500
C	3.59462700	-0.55810100	-0.61399800
C	3.85606700	-1.69497600	-1.60125900
H	4.92514100	-1.73210700	-1.83718600
H	3.55718800	-2.65931400	-1.18792200
H	3.30875700	-1.52336100	-2.53489500
C	4.24534000	-0.80091200	0.74701900
H	5.33417600	-0.82983700	0.62991500
H	3.99879200	0.01222200	1.43854000

H	3.91398200	-1.74556200	1.18038300
C	4.02994700	0.78442000	-1.19748900
H	3.77313600	1.60273700	-0.51704800
H	5.11320000	0.79011900	-1.35558000
H	3.53942900	0.96896900	-2.15919900
C	-0.40489300	0.11644400	-0.94695300
H	-1.29690900	-0.21729200	-1.48288300
H	0.38904000	0.19848700	-1.68893400
C	-0.65740900	1.46632000	-0.30508800
C	-1.63264500	2.31482800	-0.84138800
C	0.07374700	1.89073400	0.80976800
C	-1.87021400	3.57008800	-0.27984800
H	-2.21285500	1.98859700	-1.70186700
C	-0.16717500	3.14279800	1.37599600
H	0.83037300	1.23960000	1.23441300
C	-1.13808100	3.98681600	0.83319500
H	-2.63297000	4.21648100	-0.70512300
H	0.40438300	3.45830500	2.24448600
H	-1.32631500	4.95995400	1.27776400
C	-2.36762600	-1.16199600	0.74455600
C	-2.91613700	-2.24041800	-0.21886100
C	-3.15260600	-1.17332400	2.06396800
H	-2.51558200	-0.18312600	0.27940300
C	-4.42558800	-2.05979600	-0.41600800
H	-2.72355200	-3.23406600	0.20392300
H	-2.40734700	-2.19360200	-1.18924200
C	-4.65411600	-1.02536900	1.79658200
H	-2.96571200	-2.11126200	2.59572500
H	-2.80797300	-0.35777200	2.71020300
H	-4.83729500	-2.84722000	-1.04344900
H	-4.62052500	-1.09593200	-0.90689300
H	-5.22920700	-1.11621000	2.71696900
H	-4.86637700	-0.04406000	1.35059100
N	-5.11928200	-2.05542300	0.86911700
C	-5.76684100	-3.15062200	1.38280800
O	-6.17670300	-3.21998200	2.53468700
O	-5.90990900	-4.11460200	0.44355200
C	-6.59300300	-5.37668200	0.75368900
C	-8.05118800	-5.10759400	1.12829700
H	-8.58645100	-6.05831200	1.22997400
H	-8.12019200	-4.56230200	2.07044400
H	-8.54324600	-4.52221500	0.34339700
C	-5.83766400	-6.12654900	1.85212000
H	-6.27626500	-7.12176500	1.98597000

H	-4.78591300	-6.25319200	1.57175900
H	-5.88711500	-5.58983100	2.80026100
C	-6.50625300	-6.13223000	-0.57204200
H	-6.98646600	-7.11193700	-0.47960800
H	-7.00868100	-5.57442500	-1.36948100
H	-5.46135600	-6.28523400	-0.86224600

36

C	-0.54268600	-1.64356200	0.83707600
O	-0.15896700	-2.29775000	1.78292500
N	0.34675200	-1.01309500	-0.06135900
C	1.73435300	-1.26910200	0.04005100
O	2.21530100	-2.30515600	0.43682800
O	2.41358900	-0.18707600	-0.38432900
C	3.88819800	-0.18479500	-0.45504900
C	4.36618000	-1.26974400	-1.41921700
H	5.44662200	-1.17058700	-1.57054100
H	4.15921700	-2.26784200	-1.03035800
H	3.87599700	-1.15841900	-2.39286400
C	4.46648500	-0.35046900	0.94958000
H	5.55463800	-0.22944800	0.91230000
H	4.06162300	0.41472500	1.62097500
H	4.23668400	-1.33551500	1.35790300
C	4.18278300	1.20829000	-1.00745700
H	3.77407300	1.97971300	-0.34680500
H	5.26411800	1.35767200	-1.09005500
H	3.73953300	1.33519900	-2.00087800
C	-0.13082500	0.00893100	-1.00853200
H	-0.98722900	-0.39524900	-1.55380600
H	0.66216200	0.15513800	-1.74197800
C	-0.50084200	1.33644900	-0.37346700
C	-1.47625600	2.13700400	-0.97959400
C	0.11575300	1.78776900	0.79816100
C	-1.82381200	3.37224100	-0.43170000
H	-1.97021800	1.78831500	-1.88395500
C	-0.23583900	3.01977100	1.35089800
H	0.86946600	1.17377800	1.27859900
C	-1.20446300	3.81670800	0.73785200
H	-2.58499800	3.98086700	-0.91196900
H	0.24810700	3.35604700	2.26370700
H	-1.47869400	4.77433600	1.17126600
C	-2.03062900	-1.44498500	0.52371300
H	-2.23391400	-0.38792100	0.33485900
H	-2.23813500	-1.97444000	-0.41691400

C	-2.92849400	-1.94291100	1.62699300
C	-3.15437300	-3.31191500	1.81098100
C	-3.54706400	-1.03391200	2.49141000
C	-3.98556900	-3.76282700	2.83511400
H	-2.67139400	-4.02689700	1.15030400
C	-4.37913800	-1.48125500	3.51920100
H	-3.37296600	0.03134400	2.35845500
C	-4.60132700	-2.84775900	3.69272000
H	-4.15192400	-4.82867700	2.96572300
H	-4.85336500	-0.76236600	4.18188600
H	-5.24988900	-3.19887600	4.49066200

37

C	4.86496900	-0.18679100	0.43194500
C	4.84029400	-1.25265500	-0.47106000
C	3.65468200	-1.58882000	-1.12940900
C	2.48719300	-0.87622400	-0.87114600
C	2.50878000	0.19024000	0.03756700
C	3.70579700	0.54402200	0.67551800
H	5.78756900	0.07740900	0.93996200
H	5.74693500	-1.81818100	-0.66661300
H	3.64029500	-2.40700700	-1.84310900
H	1.57031000	-1.12927800	-1.39195600
H	3.71129400	1.38550500	1.36017600
C	1.32524100	1.04939300	0.27518400
O	1.39356300	2.24768400	0.47016200
N	0.03722700	0.44128500	0.19586300
C	-0.34728300	-0.86704300	0.57777900
O	-1.12278200	1.99618700	-1.55878800
O	0.39399700	-1.70426100	1.04577800
C	-2.51668100	-2.21771100	0.47639900
C	-1.79761000	-1.03649900	0.31032600
C	-2.43869800	0.10853700	-0.15505500
C	-3.78922300	0.13563300	-0.47063500
C	-4.50385200	-1.05568000	-0.30941400
C	-3.87613400	-2.21650800	0.15998300
H	-2.01541800	-3.10841100	0.83974000
H	-4.27159900	1.03952700	-0.82677200
H	-5.56224800	-1.07655900	-0.54920200
H	-4.45534000	-3.12665600	0.27857000
S	-1.32304300	1.49332500	-0.20278900
O	-1.62671600	2.40815100	0.89233800

38

C	4.68149600	-0.13314700	0.26429000
C	4.57348700	-1.15899100	-0.67937100
C	3.33607700	-1.45683800	-1.25478100
C	2.20234300	-0.74092100	-0.87952700
C	2.30554100	0.28718500	0.06815200
C	3.55388000	0.59363200	0.63100200
H	5.64419600	0.09868400	0.70998300
H	5.45501000	-1.72428800	-0.96842400
H	3.25503600	-2.24695100	-1.99522600
H	1.24505200	-0.96550700	-1.33624900
H	3.61822300	1.39904800	1.35467000
C	1.14792700	1.12070900	0.46190100
O	1.24318300	2.22127300	0.96299600
N	-0.15780000	0.57920700	0.19050800
C	-0.62392300	-0.68445200	0.64923900
C	-1.22296800	1.36538100	-0.34417500
O	-1.11600100	2.47864700	-0.80303000
O	0.04641700	-1.52705500	1.20518300
C	-3.00688800	-1.71937200	0.53857700
C	-2.07482300	-0.71275100	0.32837500
C	-2.43114600	0.50256400	-0.25817100
C	-3.73351200	0.75857900	-0.66356000
C	-4.68152600	-0.25069600	-0.45644600
C	-4.32399100	-1.47012500	0.13484700
H	-2.72064200	-2.66031200	0.99696500
H	-4.00147300	1.70629400	-1.11901000
H	-5.71204400	-0.08703700	-0.75672700
H	-5.08239700	-2.23326400	0.28104600

39

C	3.70954500	0.43249000	0.28086900
C	3.80211400	-0.67217000	-0.57139600
C	2.64592600	-1.25396900	-1.09655700
C	1.39523700	-0.74092900	-0.76375400
C	1.29716000	0.36574800	0.09181500
C	2.46293000	0.95433100	0.60707600
H	4.60925200	0.88406000	0.68780400
H	4.77647300	-1.07855600	-0.82733000
H	2.71950200	-2.10704700	-1.76419800
H	0.49913600	-1.18712700	-1.18001500
H	2.36989900	1.81343000	1.26252000
C	0.00053400	0.97570400	0.44806700
O	-0.13195700	2.06770300	0.95428600
N	-1.18518800	0.19693900	0.13850700

C	-1.47320400	-1.04471800	0.73333000
C	-2.25060800	0.71107500	-0.63063300
C	-2.86037700	-1.46731600	0.27074100
C	-3.38660100	-0.30143400	-0.57359000
H	-2.76584300	-2.39961000	-0.29529400
H	-4.25768800	0.18954900	-0.12827600
O	-2.22249300	1.75526000	-1.23781700
O	-0.73069200	-1.63898300	1.48281700
H	-3.66055400	-0.58418300	-1.59386400
H	-3.47149900	-1.68435100	1.15117600

40

C	0.27241900	0.01299000	1.11008500
O	0.21135900	0.02655200	2.31507100
N	-0.98822400	0.00362500	0.33297700
C	-1.53174200	-1.25619000	0.04362500
C	-1.53393600	1.25573600	0.01542500
C	-2.86469400	-1.26034700	-0.67344200
C	-2.86477300	1.24044400	-0.70539200
H	-2.65360400	-1.33691000	-1.75003000
H	-3.38282200	2.16637500	-0.44264400
O	-0.92394700	2.27080800	0.29961000
O	-0.91853300	-2.26339800	0.34824200
H	-2.65077800	1.28777400	-1.78309900
H	-3.38159600	-2.17910300	-0.38447400
C	1.51639100	0.00428600	0.31804900
C	1.50919300	-0.01060400	-1.08372500
C	2.73879400	0.01114000	1.00874800
C	2.71143100	-0.01856100	-1.78635200
H	0.56786500	-0.01592300	-1.62170700
C	3.93693900	0.00310700	0.30405600
H	2.72681600	0.02262600	2.09314600
C	3.92444000	-0.01181500	-1.09430200
H	2.70309400	-0.03009300	-2.87209500
H	4.88098000	0.00833600	0.84052300
H	4.86119100	-0.01814700	-1.64429400
C	-3.69091600	-0.00614100	-0.38763300
H	-3.99014500	0.00742600	0.66727000
H	-4.60807500	-0.01377200	-0.98402500

a

C	-1.38510200	0.14835400	0.01135500
H	-1.01247100	-0.88033800	0.02644500
H	-1.02730800	0.62740200	-0.91363200

H	-2.48548700	0.11233000	-0.02044600
O	-0.89231300	0.78301800	1.18178500
H	-1.21816200	1.69642600	1.17556900

b

C	-1.36600300	0.10801500	0.02317200
H	-0.96445000	-0.91881200	0.05302100
H	-2.46567200	0.02625000	0.00321100
O	-0.93119900	0.85855100	1.15471000
H	-1.24913200	0.40213400	1.94924100
C	-0.87992500	0.81396700	-1.23326000
H	-1.27814300	1.83746600	-1.23676500
H	0.21373300	0.90188300	-1.18464400
C	-1.28973000	0.08829200	-2.51844700
H	-0.89616900	-0.93779100	-2.49949300
H	-2.38449500	-0.00360700	-2.55285800
C	-0.79839500	0.79891200	-3.78108900
H	0.29588700	0.87715400	-3.79033200
H	-1.10220000	0.26149800	-4.68712300
H	-1.20285300	1.81674800	-3.84520800

c

C	-1.14038800	0.03631500	0.00291400
O	-1.62873900	1.01548500	0.91885300
H	-1.81797300	0.56231800	1.75555700
H	-0.29552400	-0.52047400	0.44074600
H	-1.92348600	-0.70050000	-0.24372200
C	-0.69644100	0.73116400	-1.25468500
C	-0.72683000	-0.02082700	-2.56247600
C	-1.71459000	1.08999900	-2.30631500
H	0.12370200	1.43134900	-1.11043600
H	-1.09503000	-1.04410700	-2.55261700
H	0.07762900	0.15372700	-3.27150600
H	-1.59078100	2.02602200	-2.84368900
H	-2.74979600	0.81844200	-2.11490600

d

O	-0.94562200	1.07780100	0.91896300
H	-1.49724300	1.00603000	1.71385000
C	-1.31310200	0.00787500	0.04722900
H	-1.29843500	-0.95217500	0.59171300
H	-2.33102200	0.14172000	-0.35196700
C	-0.32413000	-0.04891400	-1.07736100
H	0.72188900	-0.08933000	-0.77403500

C	-0.65369800	-0.07305200	-2.36807300
H	0.09768600	-0.14773400	-3.15020900
H	-1.69121800	-0.02353500	-2.69413800

e

O	-0.91158300	0.78654000	1.14779000
H	-0.72615000	0.08887200	1.79489400
C	0.33437200	1.20431300	0.60243400
H	1.05823000	1.41239100	1.40848600
H	0.77800900	0.41575700	-0.02622100
C	0.13938200	2.45377500	-0.22344300
C	1.06619600	2.78143400	-1.22005200
C	-0.93422100	3.31733700	0.01617600
C	0.92950700	3.95634300	-1.95911300
H	1.89875600	2.11020500	-1.42014600
C	-1.07676600	4.48946100	-0.72857500
H	-1.65772500	3.05771600	0.78111000
C	-0.14504600	4.81484800	-1.71576700
H	1.65616400	4.19686400	-2.73054400
H	-1.91861000	5.14957800	-0.53670100
H	-0.25712800	5.72741500	-2.29480600

f

O	-0.89909600	0.94044200	1.28799400
H	-0.74709800	0.13177700	1.80380400
C	0.31580900	1.21828200	0.59501000
H	1.15276900	1.36399000	1.29706400
H	0.59707100	0.38537200	-0.06963000
C	0.15208800	2.43003900	-0.20624400
C	0.03459200	3.42783100	-0.87504300
H	-0.07311100	4.31185500	-1.46429000

g

O	-0.31795200	1.19929400	1.71036900
H	0.01077200	0.43109900	2.20187400
C	0.42842800	1.28232000	0.50491100
H	1.51258900	1.22287600	0.70052800
H	0.18490000	0.45880600	-0.18674100
C	0.09964700	2.58624800	-0.14101900
C	-0.54301100	3.70781900	0.28735000
C	-0.51329300	4.63262200	-0.81328300
H	-0.98449800	3.85275400	1.26202900
C	0.14636600	4.00323300	-1.82195600
H	-0.93100400	5.62963600	-0.84106200

H	0.41755700	4.28031400	-2.82956100
O	0.52657200	2.74907200	-1.42873500

h

O	-0.97473600	0.62162500	0.90070400
H	-0.88399500	-0.03778400	1.60654500
C	0.31301000	1.20261700	0.69485800
H	0.76234700	1.47882100	1.66356400
H	1.00068600	0.48815500	0.21550400
C	0.17441100	2.42854700	-0.17062000
C	1.07317700	2.68817300	-1.20428600
C	-0.84849600	3.35928700	0.06957300
C	0.98026900	3.84645400	-1.98248700
H	1.86675200	1.97540900	-1.41661500
C	-0.96182800	4.50977600	-0.69776900
H	-1.56683300	3.16414800	0.85969800
C	-0.04427700	4.76411000	-1.72953300
H	1.69620100	4.01293500	-2.77855200
H	-1.75446900	5.22998300	-0.51953500
O	-0.23764300	5.92070300	-2.42489400
C	0.66170100	6.21779500	-3.48270200
H	0.62655300	5.45311200	-4.26981400
H	0.33379000	7.17358000	-3.89677400
H	1.69355000	6.31703800	-3.12056600

i

C	-1.40164700	0.12361600	-0.01809900
H	-1.03952000	-0.91826600	-0.03750600
O	-0.86746800	0.79512300	1.12848400
H	-1.23572000	0.36164300	1.91507900
C	-0.85364500	0.85272700	-1.23674200
H	-1.17711100	0.36060200	-2.16045600
H	-1.20900200	1.88976100	-1.25156000
H	0.24115000	0.86607100	-1.21518700
C	-2.92888400	0.11214400	0.01483600
H	-3.34023800	-0.39914900	-0.86362800
H	-3.29956600	-0.41080200	0.90635000
H	-3.31644200	1.13742700	0.03445500

j

O	-1.83248800	0.04831500	-0.06328400
C	-0.53706100	0.01008600	0.47132500
C	0.49301900	-0.99972800	-0.07731100

C	0.50495700	1.06498600	0.04299700
H	-0.65854400	-0.05238300	1.55522700
H	0.20904100	-1.40960200	-1.05982400
H	0.81310800	-1.81985400	0.57583200
H	0.22715900	1.58975200	-0.88510500
H	0.83387400	1.79987400	0.78684500
O	1.49528800	0.03673300	-0.18766500
H	-1.75540100	0.10384900	-1.03084900

k

O	0.03916400	1.66986100	2.17665300
H	0.33202700	0.91690200	2.71423300
C	0.37238100	1.36854900	0.82257700
H	-0.31644300	0.60373800	0.42979600
C	0.19519300	2.63170700	-0.00520000
C	0.26541900	2.55086500	-1.40153000
C	-0.01551500	3.87556500	0.59474000
C	0.12791700	3.69386500	-2.18595800
H	0.43733900	1.58725300	-1.87500200
C	-0.15303300	5.02235500	-0.19191200
H	-0.07762500	3.93793400	1.67475700
C	-0.08159700	4.93683500	-1.58200200
H	0.18439800	3.61559900	-3.26834800
H	-0.31818500	5.98421000	0.28648900
H	-0.18945400	5.82943300	-2.19209600
C	1.78826200	0.82381000	0.71005800
C	2.04431700	-0.37197800	0.03428600
C	2.85534600	1.52488900	1.28764600
C	3.34889900	-0.86073200	-0.07422900
H	1.21845000	-0.92554900	-0.40649300
C	4.15567700	1.03611100	1.18635200
H	2.65628700	2.45395600	1.81369300
C	4.40656800	-0.15774900	0.50217700
H	3.53540900	-1.79165000	-0.60272700
H	4.97677400	1.58697800	1.63707000
H	5.42168500	-0.53652600	0.42172600

l

O	-1.10918900	0.82594500	1.21576400
H	-0.53603000	1.56773800	0.96252500
C	-1.48164100	0.14831500	0.01454200
C	-0.25836500	-0.27643600	-0.80433200
C	-2.31804600	-1.06387200	0.41684500
H	-2.10714000	0.81099300	-0.60997900

C	-0.66556300	-1.09289600	-2.03916900
H	0.39411600	-0.87693000	-0.15543100
H	0.31478700	0.61252600	-1.10442600
C	-2.73439300	-1.88511500	-0.81115800
H	-1.71583200	-1.68394200	1.09524800
H	-3.19584300	-0.72844100	0.98188000
C	-1.51483700	-2.30789600	-1.64188000
H	0.22704900	-1.41199500	-2.59097900
H	-1.24526900	-0.45468700	-2.72182200
H	-3.30553900	-2.76626100	-0.49446000
H	-3.40773500	-1.28432900	-1.43970500
H	-1.83616500	-2.85527100	-2.53685500
H	-0.89818100	-3.00033200	-1.05083800

m

C	-1.39839500	0.10147400	-0.02122400
O	-0.89137400	0.81591000	1.12072600
H	-1.21576300	0.35411100	1.91143800
C	-0.86275700	0.86021800	-1.23466800
H	-1.19281100	0.38757400	-2.16588300
H	-1.21909600	1.89596800	-1.22533800
H	0.23242200	0.87576400	-1.22219500
C	-2.93156800	0.12002300	0.00041300
H	-3.35109700	-0.37900100	-0.88088900
H	-3.31400900	-0.39876000	0.88945500
H	-3.29917400	1.15152300	0.02330800
C	-0.87078300	-1.33834900	-0.00202900
H	-1.20395200	-1.89973300	-0.88275700
H	0.22435100	-1.34238500	0.01811800
H	-1.23041400	-1.87231700	0.88755100

PhCOOMe

C	0.229319	0.121209	-0.000422
C	0.749508	-1.180352	0.001036
C	1.099283	1.219745	-0.001639
C	2.129254	-1.376427	0.001571
H	0.074308	-2.028165	0.002058
C	-1.233804	0.391837	0.000360
C	2.477010	1.019610	-0.001525
H	0.677727	2.219145	-0.002580
C	2.993542	-0.278895	0.000246
H	2.530832	-2.385626	0.002958
O	-1.968565	-0.740001	-0.003102
O	-1.728682	1.504257	0.003672

H	3.148374	1.873394	-0.002770
H	4.068703	-0.435196	0.000451
C	-3.391706	-0.550414	-0.000504
H	-3.820409	-1.553389	-0.009116
H	-3.708752	0.007101	-0.886180
H	-3.707235	-0.009188	0.895871

PhMe

C	-0.913743	0.003409	-0.010143
C	-2.422926	0.001478	0.007858
C	-0.192841	1.203998	-0.007566
H	-2.831472	-0.773210	-0.651084
H	-2.807870	-0.198196	1.017027
H	-2.829043	0.966728	-0.312079
C	-0.197005	-1.201642	-0.007688
C	1.203426	1.204139	0.001820
H	-0.732108	2.148415	-0.014264
C	1.197685	-1.207262	0.001877
H	-0.740241	-2.144067	-0.014380
C	1.904342	-0.002398	0.007144
H	1.742689	2.147793	0.001536
H	1.733569	-2.152875	0.001705
H	2.990854	-0.004919	0.011725

Morpholine

O	-0.000032	-1.394306	-0.275714
C	1.176421	-0.747461	0.199981
C	-1.176459	-0.747407	0.199974
C	1.206028	0.722217	-0.221553
H	2.024018	-1.302695	-0.216575
H	1.225005	-0.819836	1.301967
C	-1.205991	0.722275	-0.221547
H	-2.024075	-1.302595	-0.216603
H	-1.225072	-0.819800	1.301959
N	0.000038	1.453096	0.189598
H	2.088403	1.222130	0.195371
H	1.275551	0.777392	-1.315993
H	-2.088330	1.222230	0.195398
H	-1.275532	0.777471	-1.315986
H	0.000042	1.530735	1.207854

MeOH

C	-0.662541	-0.019276	0.000000
O	0.749980	0.122959	-0.000002

H	-1.035953	-0.545528	-0.892602
H	1.131385	-0.768701	0.000029
H	-1.084002	0.990541	-0.000686
H	-1.036025	-0.544329	0.893276

Ester product of rxn between 22 and a

C	2.73928200	0.83683800	0.57102700
C	3.15132700	-0.38767700	0.03842500
C	2.22563200	-1.22710300	-0.58617500
C	0.88840800	-0.84636000	-0.68022000
C	0.47294900	0.38133200	-0.14646300
C	1.40420400	1.22085400	0.47927700
H	3.45868700	1.48992700	1.05641500
H	4.19315900	-0.68749900	0.11005800
H	2.54625600	-2.17876200	-1.00006300
H	0.16557400	-1.49313600	-1.16385800
H	1.06320100	2.16688200	0.88581900
C	-0.94081200	0.84003200	-0.21713900
O	-1.34554600	1.89735800	0.23062600
O	-1.74218000	-0.05055400	-0.83829300
C	-3.12289300	0.32952300	-0.94041000
H	-3.22700900	1.25717800	-1.51005900
H	-3.61486500	-0.49313800	-1.46100800
H	-3.55988100	0.46733600	0.05254500

Ester product of rxn between 22 and b

C	2.64839600	0.42901800	1.04516500
C	3.18503200	-0.49052200	0.13996100
C	2.38771000	-1.01638300	-0.87956100
C	1.05505200	-0.62620400	-0.99705400
C	0.51476400	0.29594200	-0.09023400
C	1.31752600	0.82161100	0.93077100
H	3.26769500	0.83846800	1.83797200
H	4.22360900	-0.79696400	0.22894000
H	2.80483300	-1.73102900	-1.58322800
H	0.43139500	-1.03061100	-1.78588700
H	0.88132300	1.53448900	1.62219900
C	-0.90139000	0.74918200	-0.16820700
O	-1.40806900	1.55228000	0.59472600
O	-1.57147200	0.17048500	-1.18379300
C	-2.95550400	0.55651500	-1.33393600
C	-3.52235100	-0.20717600	-2.51714400
H	-3.48975100	0.32553100	-0.40626100
C	-4.99529900	0.13619300	-2.76589600

H	-2.92893800	0.02369700	-3.41150500
H	-3.41559900	-1.28431100	-2.33360500
C	-5.58315600	-0.62639700	-3.95455300
H	-5.09331100	1.21691200	-2.93799900
H	-5.57887400	-0.08582700	-1.86182000
H	-5.03788200	-0.39973500	-4.87914000
H	-5.52977900	-1.71048700	-3.79519100
H	-3.00542700	1.63991300	-1.48642600
H	-6.63523800	-0.36402100	-4.11462500

Ester product of rxn between 22 and c

C	2.44611800	0.11070900	1.20226800
C	2.95809700	-0.79637500	0.27060800
C	2.21989400	-1.12011900	-0.87046700
C	0.97106400	-0.53951500	-1.08358100
C	0.45578900	0.37053000	-0.15039600
C	1.19863300	0.69256900	0.99311000
H	3.01949700	0.36266400	2.08970200
H	3.93146900	-1.25074200	0.43376700
H	2.61774400	-1.82556400	-1.59428900
H	0.39263600	-0.78689000	-1.96619100
H	0.78233400	1.39843000	1.70377000
C	-0.87302600	1.01920500	-0.32568300
O	-1.36248700	1.80549800	0.46613200
O	-1.48470100	0.63841200	-1.46322400
C	-2.78705900	1.22219300	-1.70986500
H	-3.44557300	0.98356600	-0.86809100
H	-2.67802000	2.31092600	-1.75184200
C	-3.31070900	0.65551400	-2.99520800
C	-3.95732600	-0.70703400	-2.99360000
C	-4.80486100	0.52607700	-3.17048100
H	-2.73513200	0.92501100	-3.87786900
H	-4.01414700	-1.23454100	-2.04479200
H	-3.79446100	-1.34998900	-3.85375700
H	-5.22028800	0.73383200	-4.15228100
H	-5.44181900	0.83161900	-2.34408800

Ester product of rxn between 22 and d

C	2.57538900	0.28562000	1.12186600
C	3.19189800	-0.35603200	0.04429300
C	2.45118500	-0.68088500	-1.09483100
C	1.09518900	-0.36653500	-1.16047400
C	0.47505800	0.27698800	-0.08068200
C	1.22093700	0.60174700	1.06021100

H	3.15089200	0.53867300	2.00756300
H	4.24898000	-0.60247800	0.09215200
H	2.93081700	-1.17934100	-1.93223300
H	0.51488100	-0.61506600	-2.04148300
H	0.72281200	1.10002500	1.88490100
C	-0.96877700	0.63727100	-0.09565200
O	-1.54703400	1.20088400	0.81599100
O	-1.57868900	0.27100500	-1.24175200
C	-2.99034400	0.58391000	-1.33035600
H	-3.11983800	1.67069100	-1.33864700
H	-3.48533700	0.19203100	-0.43445100
C	-3.51723300	-0.05077300	-2.57651700
H	-3.36489100	-1.12595000	-2.66312400
C	-4.16752900	0.61931100	-3.52682200
H	-4.32703500	1.69388700	-3.46087900
H	-4.57244500	0.11976500	-4.40304700

Ester product of rxn between 22 and e

C	2.76197900	0.48156600	0.83967200
C	3.21242500	-0.32324100	-0.21026300
C	2.31309400	-0.78758700	-1.17334100
C	0.96367400	-0.44981300	-1.09090800
C	0.51030200	0.35743900	-0.03835300
C	1.41463400	0.82135800	0.92608800
H	3.46101500	0.84285700	1.58831900
H	4.26390500	-0.58833200	-0.27799500
H	2.66388300	-1.41290000	-1.98923600
H	0.26024500	-0.80564100	-1.83485300
H	1.04356300	1.44444400	1.73272300
C	-0.91881100	0.74774700	0.10038100
O	-1.36219100	1.43610500	1.00173200
O	-1.68277700	0.25036100	-0.89427400
C	-3.09499500	0.55580800	-0.82139300
H	-3.44915900	0.30552200	0.18395000
H	-3.23671300	1.63082500	-0.96632700
C	-3.79915000	-0.24470200	-1.88113600
C	-3.66567500	-1.63924100	-1.91747100
C	-4.61108800	0.38655700	-2.82727500
C	-4.33218500	-2.38663500	-2.88605200
H	-3.03059200	-2.13397600	-1.18764100
C	-5.28798100	-0.36168000	-3.79318800
H	-4.71381400	1.46866000	-2.80827600
C	-5.14803200	-1.74901100	-3.82543400
H	-4.21922500	-3.46705200	-2.90764100

H	-5.91744800	0.14076800	-4.52233200
H	-5.66983500	-2.33256800	-4.57879300

Ester product of rxn between 22 and f

C	2.58901100	0.20344900	1.19259700
C	3.18919300	-0.48431100	0.13436000
C	2.46028600	-0.76274100	-1.02451000
C	1.13183900	-0.35596200	-1.12983600
C	0.52850300	0.33393200	-0.06905100
C	1.26213300	0.61207600	1.09221000
H	3.15571300	0.42009700	2.09340200
H	4.22480800	-0.80321600	0.21279500
H	2.92750400	-1.29719800	-1.84644900
H	0.56086500	-0.56948600	-2.02599600
H	0.77704800	1.14677200	1.90170100
C	-0.88423000	0.79121700	-0.12381400
O	-1.45550100	1.39215400	0.76730900
O	-1.48599000	0.46454400	-1.29063300
C	-2.86444900	0.88257100	-1.39834500
H	-3.44155000	0.44198400	-0.57805800
H	-2.92195000	1.97122000	-1.29059800
C	-3.37367900	0.45199300	-2.69293600
C	-3.82338500	0.10777300	-3.75811700
H	-4.21759500	-0.19973900	-4.70197600

Ester product of rxn between 22 and g

C	2.60907700	0.35355800	1.15539800
C	3.29945800	-0.03285300	0.00338500
C	2.60698100	-0.24237600	-1.19174500
C	1.22539800	-0.06715500	-1.23961300
C	0.53146500	0.32052300	-0.08504500
C	1.22884200	0.53029200	1.11206200
H	3.14722600	0.51669400	2.08462000
H	4.37664800	-0.17059600	0.03700000
H	3.14415700	-0.54205100	-2.08693700
H	0.68181300	-0.22788000	-2.16345600
H	0.67352900	0.83029700	1.99422300
C	-0.94243700	0.52322200	-0.07796800
O	-1.58723400	0.86818700	0.89613700
O	-1.50038500	0.27891300	-1.28191000
C	-2.93736500	0.47589900	-1.35160300
H	-3.42685600	-0.18787100	-0.63479000
H	-3.15934100	1.50935800	-1.06981500
C	-3.39017100	0.17004000	-2.72534800

C	-4.04421800	-0.89364800	-3.27645800
C	-4.19769800	-0.60201300	-4.67165100
H	-4.37511400	-1.77869000	-2.75063200
C	-3.62305700	0.61788800	-4.86457500
H	-4.67260900	-1.21725900	-5.42296500
H	-3.49118800	1.25130400	-5.72919400
O	-3.12911600	1.10378500	-3.69303900

Ester product of rxn between 22 and h

C	2.50323500	-0.21220000	1.13301800
C	2.83263300	-1.18590500	0.18615600
C	1.98041500	-1.43016000	-0.89362200
C	0.79902700	-0.70385400	-1.03056400
C	0.46727200	0.27329600	-0.08185500
C	1.32392200	0.51585900	0.99994700
H	3.16564000	-0.02201800	1.97252200
H	3.75303600	-1.75390900	0.28969500
H	2.23689000	-2.18698800	-1.62928000
H	0.13290500	-0.88709700	-1.86581000
H	1.04915400	1.27663800	1.72268000
C	-0.77939100	1.08104300	-0.18198800
O	-1.10210800	1.94996300	0.60862700
O	-1.52156900	0.74418400	-1.25507100
C	-2.74118500	1.50918800	-1.44647100
H	-3.40483000	1.33472300	-0.59478500
H	-2.47615000	2.57103400	-1.45295100
C	-3.36655100	1.07167500	-2.73684600
C	-4.57515600	0.37650400	-2.75376200
C	-2.74114700	1.35200200	-3.96271300
C	-5.16682800	-0.03065300	-3.95299100
H	-5.07305300	0.14525000	-1.81539500
C	-3.30815300	0.94877900	-5.16183700
H	-1.79625800	1.88919600	-3.97102800
C	-4.52996900	0.25497200	-5.16535700
H	-6.10870500	-0.56549000	-3.92813700
H	-2.82996500	1.16245600	-6.11253600
O	-5.00875100	-0.09174000	-6.39081300
C	-6.24567300	-0.78909000	-6.45077500
H	-6.44112600	-0.96215000	-7.51093500
H	-6.18994000	-1.75465400	-5.93162400
H	-7.06435300	-0.19466400	-6.02489100

Ester product of rxn between 22 and i

C	2.63710400	0.20123400	1.22875900

C	3.24541200	-0.44924400	0.15188800
C	2.51036400	-0.72800300	-1.00291800
C	1.16907400	-0.35850600	-1.08452600
C	0.55651900	0.29435900	-0.00608300
C	1.29736100	0.57208300	1.15041700
H	3.20761300	0.41863000	2.12714600
H	4.29095200	-0.73853300	0.21256700
H	2.98288400	-1.23368600	-1.84013600
H	0.59327000	-0.57226900	-1.97736600
H	0.80625900	1.07811400	1.97454800
C	-0.87467000	0.71538600	-0.03572800
O	-1.42443500	1.28847300	0.88924100
O	-1.46871600	0.38615600	-1.19653100
C	-2.87428500	0.71725200	-1.42739300
C	-3.09344500	2.22526800	-1.46306800
H	-4.10350700	2.43414000	-1.83397700
H	-2.98646700	2.66695300	-0.47054100
H	-2.37742700	2.70079500	-2.14208000
C	-3.79133800	-0.02429900	-0.46157900
H	-3.54669200	-1.09188200	-0.44384200
H	-3.70975100	0.37400300	0.55138500
H	-4.82911700	0.08035800	-0.79848800
H	-3.02280500	0.31492200	-2.43384300

Ester product of rxn between 22 and j

C	2.40636500	-0.19615100	1.31263800
C	3.21478200	-0.38142400	0.18777500
C	2.67354300	-0.25447900	-1.09392600
C	1.32539500	0.05796400	-1.25556400
C	0.51303900	0.24459100	-0.12833000
C	1.05887400	0.11573700	1.15613300
H	2.82725200	-0.29553500	2.30884400
H	4.26648100	-0.62494400	0.31007100
H	3.30246700	-0.39921200	-1.96743100
H	0.89964700	0.15785600	-2.24725900
H	0.41354600	0.26303900	2.01538300
C	-0.92946500	0.57871800	-0.23956400
O	-1.67900500	0.74718000	0.70528300
O	-1.34472800	0.67895200	-1.52455800
C	-2.72451200	1.01714900	-1.68649200
C	-3.20768600	0.83317900	-3.12627300
C	-3.74998200	-0.06953200	-1.34160000
H	-2.91832800	1.99150600	-1.23499000
H	-2.47414500	0.32847700	-3.76825600

H	-3.59295400	1.72395900	-3.63514300
H	-3.29834200	-1.03483900	-1.08133500
H	-4.51206100	0.19430900	-0.60144100
O	-4.26705100	-0.05341300	-2.69622000

Ester product of rxn between 22 and k

C	2.48931100	0.18183100	1.30301000
C	2.99100400	-0.72546900	0.36585500
C	2.21092600	-1.10586600	-0.72893600
C	0.93022100	-0.58121100	-0.89125900
C	0.42569900	0.32941100	0.04776900
C	1.21015600	0.70801100	1.14542600
H	3.09554700	0.47750400	2.15429500
H	3.98938900	-1.13598000	0.48884700
H	2.60064400	-1.81234400	-1.45606200
H	0.31847000	-0.87392500	-1.73670700
H	0.80156300	1.41334000	1.86101100
C	-0.93218800	0.92347800	-0.07662400
O	-1.42039500	1.70047500	0.72150600
O	-1.56994500	0.49384300	-1.19098500
C	-2.88564700	1.04680500	-1.44888300
H	-3.35853200	1.23654100	-0.48264100
C	-2.77422800	2.35498600	-2.21034900
C	-3.91672800	3.15394900	-2.33910600
C	-1.58149400	2.76765900	-2.80858400
C	-3.86643600	4.34941800	-3.05238400
H	-4.84844600	2.83376300	-1.87932400
C	-1.53083300	3.96695200	-3.52381200
H	-0.69111000	2.15597000	-2.71069200
C	-2.67075600	4.76022500	-3.64833200
H	-4.75898000	4.96260800	-3.14111500
H	-0.59632300	4.27925600	-3.98188100
H	-2.62998600	5.69331700	-4.20322900
C	-3.66045300	-0.02010100	-2.19339100
C	-4.64743300	-0.75376700	-1.52954500
C	-3.38667700	-0.29894400	-3.53783200
C	-5.35545200	-1.75460900	-2.19772300
H	-4.86077200	-0.54085700	-0.48500600
C	-4.08671900	-1.30266900	-4.20429300
H	-2.63125800	0.27955600	-4.06134400
C	-5.07455000	-2.03158000	-3.53585000
H	-6.12390700	-2.31567000	-1.67326300
H	-3.86742600	-1.51293300	-5.24743300
H	-5.62435500	-2.80963000	-4.05816900

Ester product of rxn between 22 and l

C	2.58102700	0.24402500	1.20207500
C	3.21049100	-0.44507400	0.16195200
C	2.49943700	-0.76511800	-0.99725300
C	1.16039100	-0.39909400	-1.11978200
C	0.52718100	0.29255200	-0.07808900
C	1.24378300	0.61271600	1.08257700
H	3.13328500	0.49328300	2.10356600
H	4.25434400	-0.73198800	0.25448600
H	2.98904900	-1.30005900	-1.80594800
H	0.60251800	-0.64412100	-2.01607100
H	0.73606800	1.14879500	1.87714600
C	-0.90006700	0.71154800	-0.15572200
O	-1.47696300	1.32889700	0.72257100
O	-1.48334500	0.32616900	-1.30719900
C	-2.87678700	0.68914600	-1.49530800
C	-2.98539400	2.14550000	-1.98177600
C	-3.46740300	-0.28403900	-2.50513400
H	-3.37304000	0.57475400	-0.52793900
C	-4.24158100	2.36562900	-2.85455200
H	-2.08200700	2.37938000	-2.55713100
H	-2.99423500	2.80971900	-1.11217200
C	-4.99045200	-0.04906300	-2.63777800
H	-2.96900400	-0.13100700	-3.47066800
H	-3.25806000	-1.31424500	-2.19851400
C	-5.37853900	1.43272400	-2.42134100
H	-4.00367700	2.16990500	-3.90840400
H	-4.55484100	3.41401400	-2.79707100
H	-5.52599300	-0.67599600	-1.91512500
H	-5.31150900	-0.37761500	-3.63309800
H	-5.59608000	1.61042000	-1.35958400
H	-6.29989200	1.66379200	-2.96803400

Ester product of rxn between 22 and m

C	2.60810700	0.18126000	1.19903300
C	3.20639200	-0.45619800	0.10887600
C	2.46239000	-0.71479800	-1.04491100
C	1.12225600	-0.33825700	-1.11210100
C	0.51981300	0.30183800	-0.02042000
C	1.26960800	0.55950900	1.13486700
H	3.18539800	0.38286100	2.09678700
H	4.25096000	-0.75107400	0.15844300
H	2.92710000	-1.21029500	-1.89255300

H	0.53898000	-0.53620000	-2.00370700
H	0.78642900	1.05595100	1.96944700
C	-0.91040900	0.73027300	-0.03545200
O	-1.44661300	1.29834300	0.90087800
O	-1.51030000	0.41181900	-1.19472000
C	-2.92423400	0.73719700	-1.45252100
C	-3.12033600	2.25351500	-1.42645600
H	-4.13893100	2.49491700	-1.74987300
H	-2.96716300	2.65568400	-0.42401900
H	-2.42039800	2.74034900	-2.11442200
C	-3.14107400	0.18558700	-2.86059600
H	-4.17146600	0.37021000	-3.18151400
H	-2.46471900	0.66806100	-3.57401500
H	-2.95855800	-0.89388900	-2.88665200
C	-3.81812500	0.01543500	-0.44330900
H	-3.59454600	-1.05705500	-0.43508700
H	-3.68132700	0.41181900	0.56369000
H	-4.86784100	0.14173300	-0.73093400

Ester product of rxn between 34 and a

C	-1.03108000	0.59762200	0.05743500
O	-1.61700400	1.16958900	0.95376400
O	-1.62338600	0.23149300	-1.09928400
C	-3.01076800	0.58526100	-1.22351700
H	-3.14027500	1.66923600	-1.15956200
H	-3.31960000	0.22343400	-2.20516400
H	-3.60327600	0.10689400	-0.43867000
C	0.43843000	0.22773000	0.05482500
C	1.22155300	1.14548500	-0.91119800
C	1.05356100	0.31275500	1.45618600
H	0.51887400	-0.80060000	-0.32320900
C	2.71448000	0.82809700	-0.83026400
H	1.06149500	2.19282200	-0.62582500
H	0.86456700	1.01729000	-1.93869600
C	2.55745100	0.05131500	1.38568300
H	0.87445100	1.31107800	1.86906900
H	0.58079500	-0.40922800	2.13235600
H	3.29530600	1.52761100	-1.43937600
H	2.89987400	-0.19130200	-1.21260000
H	3.02654300	0.19835700	2.36315400
H	2.74577500	-0.99101300	1.07267300
O	3.21295100	0.94659300	0.49470000

Ester product of rxn between 34 and b

C	-0.95440100	0.62874300	-0.07853700
O	-1.47968500	1.35946700	0.73849500
O	-1.59683300	0.12369100	-1.14877500
C	-2.98434100	0.50726100	-1.29814600
C	-3.52296800	-0.17855300	-2.54073000
H	-3.53198400	0.20878000	-0.39803800
C	-4.99529700	0.16563000	-2.79218900
H	-2.91699900	0.11835400	-3.40672400
H	-3.40718800	-1.26464000	-2.42970100
C	-5.55428300	-0.52259500	-4.03878400
H	-5.10270500	1.25433100	-2.89412700
H	-5.59175000	-0.12168500	-1.91530300
H	-4.99551500	-0.23060900	-4.93659500
H	-5.49226800	-1.61427400	-3.94995100
H	-3.04213400	1.59819800	-1.37646600
H	-6.60615000	-0.26022200	-4.20020500
C	0.49594600	0.19390200	-0.03685700
C	1.41496100	1.39293000	-0.35403200
C	0.86310200	-0.36942400	1.34758900
H	0.64766500	-0.57915700	-0.79858600
C	2.87882300	0.99134900	-0.17910500
H	1.17868200	2.21471100	0.33152400
H	1.24617900	1.74852200	-1.37800400
C	2.36170400	-0.65817100	1.41722000
H	0.59450100	0.36629400	2.11323000
H	0.29932000	-1.28769800	1.55419100
H	3.53859400	1.85360300	-0.31640200
H	3.15222000	0.23083800	-0.93181200
H	2.65150800	-0.97567800	2.42355000
H	2.62199600	-1.47133000	0.71670700
O	3.14054800	0.49557400	1.12689600

Ester product of rxn between 34 and c

C	-0.90251800	0.97750600	-0.30927600
O	-1.40107800	1.73968600	0.49637200
O	-1.49213100	0.62663500	-1.46692300
C	-2.78752800	1.22671800	-1.72673300
H	-3.45433700	1.00240700	-0.88786900
H	-2.66282400	2.31377200	-1.77356100
C	-3.31156800	0.66097700	-3.01230300
C	-3.98693100	-0.68739900	-3.00613500
C	-4.80668100	0.56191200	-3.19990300
H	-2.72316700	0.91103000	-3.89220900
H	-4.06388100	-1.20616400	-2.05385800

H	-3.83030400	-1.34065500	-3.85964900
H	-5.20914300	0.77049100	-4.18691900
H	-5.44422600	0.88732000	-2.38158200
C	0.45437400	0.32369600	-0.14750800
C	1.56971100	1.38264300	-0.27650900
C	0.57159600	-0.36685200	1.22314700
H	0.57738000	-0.41950300	-0.94343900
C	2.92861300	0.75145300	0.02328800
H	1.37849800	2.19131700	0.43807400
H	1.57505800	1.81692600	-1.28388900
C	1.99332200	-0.88625800	1.43069700
H	0.32632300	0.35707400	2.00762700
H	-0.14079200	-1.19772700	1.30012800
H	3.71900000	1.50839200	0.02085400
H	3.17344300	0.00176700	-0.74980500
H	2.11447200	-1.30054600	2.43630200
H	2.21082200	-1.68930700	0.70440300
O	2.96135900	0.14826800	1.30986300

Ester product of rxn between 34 and d

C	-1.08064600	0.18557300	0.01910700
O	-1.76691700	0.37712200	1.00280400
O	-1.56854800	0.17907000	-1.23877000
C	-2.99120300	0.43399400	-1.36345500
H	-3.20578900	1.45592500	-1.03525600
H	-3.52120400	-0.25330900	-0.69415800
C	-3.36913600	0.21527800	-2.79250500
H	-3.12322400	-0.76281700	-3.20429800
C	-3.99890200	1.12339900	-3.53670000
H	-4.25087700	2.10731400	-3.14551500
H	-4.29414900	0.91661300	-4.56205400
C	0.41886600	-0.02766400	0.02397300
C	1.13915500	1.32617400	-0.17243900
C	0.89875200	-0.67688400	1.32902400
H	0.67177700	-0.67195400	-0.82717400
C	2.65048900	1.13070600	-0.05530400
H	0.80570000	2.02828200	0.60150400
H	0.89003100	1.75575700	-1.14954000
C	2.42505000	-0.74148200	1.35293300
H	0.54218900	-0.08363100	2.17740200
H	0.48259300	-1.68604200	1.43576800
H	3.17202300	2.09160100	-0.10294600
H	3.01245400	0.50523600	-0.89027800
H	2.78530300	-1.11781100	2.31512900

H	2.78719400	-1.42498200	0.56460400
O	3.01515900	0.54192900	1.18523500

Ester product of rxn between 34 and e

C	-0.91794700	1.20224100	-0.12549500
O	-1.36754100	2.04727900	0.62206500
O	-1.68588700	0.42492900	-0.91681600
C	-3.11812500	0.64920200	-0.82117700
H	-3.42722400	0.51458800	0.21877700
H	-3.32220900	1.68680500	-1.10230300
C	-3.79872400	-0.32585400	-1.73808600
C	-4.39463300	-1.48463000	-1.22977000
C	-3.82737200	-0.09451600	-3.11915700
C	-5.01369300	-2.39755000	-2.08518500
H	-4.37444500	-1.67025900	-0.15874800
C	-4.44084500	-1.00614000	-3.97683000
H	-3.36489700	0.80414400	-3.51974700
C	-5.03684900	-2.15962400	-3.46016000
H	-5.47710600	-3.29201400	-1.67841800
H	-4.45874900	-0.81618100	-5.04638800
H	-5.51932700	-2.86832800	-4.12750600
C	0.54899700	0.85633200	-0.29059300
C	1.45987800	1.86098900	0.42338400
C	0.83716700	-0.56954800	0.22909700
H	0.76385800	0.86628400	-1.36852900
C	2.91695800	1.41226100	0.31949900
H	1.17182300	1.92520800	1.47816700
H	1.34544900	2.86289600	-0.00633600
C	2.33458700	-0.86068500	0.13623500
H	0.52169200	-0.64496200	1.27751700
H	0.27270800	-1.31030900	-0.34674700
H	3.57083700	2.06822200	0.90205000
H	3.24993000	1.44995900	-0.73294700
H	2.57188700	-1.83039700	0.58458800
H	2.64299300	-0.89129600	-0.92375900
O	3.10938200	0.10146300	0.83822600

Ester product of rxn between 34 and f

C	-0.91295100	0.73801900	-0.10937200
O	-1.48627400	1.30594900	0.79814600
O	-1.50272200	0.44695900	-1.28984500
C	-2.88419000	0.86800100	-1.39688000
H	-3.47031300	0.38255500	-0.60894400
H	-2.94560100	1.94889900	-1.23065900

C	-3.37431000	0.50721200	-2.71939200
C	-3.80753000	0.22118200	-3.80841200
H	-4.18704800	-0.03386800	-4.77370400
C	0.52846500	0.27981600	-0.07431300
C	1.47122200	1.49589700	0.03854800
C	0.76834200	-0.65895700	1.12414600
H	0.74503700	-0.25631600	-1.00486200
C	2.91292700	1.02367000	0.22052700
H	1.17325600	2.09952200	0.90320900
H	1.39668700	2.12709800	-0.85552300
C	2.25498400	-0.99331900	1.23732700
H	0.43389800	-0.15955200	2.03998900
H	0.18750500	-1.58294600	1.01339900
H	3.58140800	1.87338300	0.38936200
H	3.25443900	0.49799000	-0.68848600
H	2.45282300	-1.58807100	2.13424600
H	2.57602300	-1.58323600	0.36078800
O	3.05513400	0.17594700	1.35232000

Ester product of rxn between 34 and g

C	-0.52889600	-0.63959400	-0.59094500
O	-0.64659300	-1.48092000	-1.46010700
O	0.66173200	-0.22644600	-0.11240900
C	1.82695100	-0.85120500	-0.72087000
H	1.79034500	-1.92855300	-0.54207100
H	1.78626900	-0.68086500	-1.80048900
C	3.04590100	-0.27234200	-0.11741200
C	3.94051800	-0.73372000	0.80444800
C	4.91600000	0.30146900	0.98275000
H	3.90717400	-1.69561600	1.29733000
C	4.53990100	1.31667400	0.15609900
H	5.77738400	0.28686600	1.63572100
H	4.94949600	2.29218600	-0.06005700
O	3.40747000	0.98530900	-0.52263200
C	-1.68337600	0.06478500	0.09000300
C	-2.54712800	0.81208200	-0.94497000
C	-2.56404200	-0.95428900	0.84119300
H	-1.27433900	0.78486700	0.80736200
C	-3.78780000	1.39368600	-0.26897300
H	-2.85214200	0.10954600	-1.72826800
H	-1.97182300	1.61640400	-1.41983900
C	-3.80334900	-0.26036000	1.40438300
H	-2.87153800	-1.74158600	0.14402200
H	-2.00041800	-1.42635100	1.65543500

H	-4.45421000	1.85229800	-1.00589700
H	-3.49132900	2.17433200	0.45353700
H	-4.48051200	-0.98606800	1.86522700
H	-3.50700300	0.46807200	2.17960300
O	-4.55345200	0.39253900	0.38889400

Ester product of rxn between 34 and h

C	-0.74591000	1.17370600	-0.29916000
O	-0.97404000	2.13794500	0.40358400
O	-1.55795600	0.76855000	-1.29618700
C	-2.73118000	1.59345600	-1.53836600
H	-3.39951500	1.51525100	-0.67590200
H	-2.40329500	2.63431700	-1.61579100
C	-3.38791900	1.11149200	-2.79662100
C	-4.60326600	0.42848200	-2.76361700
C	-2.78046900	1.33088600	-4.04369800
C	-5.21791500	-0.02582900	-3.93392200
H	-5.08849600	0.24483100	-1.80815800
C	-3.37081500	0.88154700	-5.21482300
H	-1.83132100	1.85869800	-4.09108300
C	-4.59843500	0.19992400	-5.16791600
H	-6.16471800	-0.54891800	-3.87070800
H	-2.90700100	1.04919500	-6.18174400
O	-5.10079900	-0.19525500	-6.36888600
C	-6.34499200	-0.88192000	-6.37763400
H	-6.55994200	-1.09963500	-7.42570200
H	-6.28943900	-1.82405800	-5.81715500
H	-7.15079500	-0.26106000	-5.96509900
C	0.47704700	0.28160500	-0.20737300
C	1.35926600	0.43816600	-1.46613100
C	1.30893300	0.57093200	1.04681800
H	0.11941100	-0.75734700	-0.18001200
C	2.63091400	-0.39615800	-1.31435200
H	1.63482800	1.49357900	-1.58747200
H	0.80944400	0.12825100	-2.36098800
C	2.58300400	-0.27213400	1.03508700
H	1.57380600	1.63347500	1.06868600
H	0.73015900	0.36142100	1.95387900
H	3.31198300	-0.22621500	-2.15398000
H	2.37164000	-1.46959100	-1.29816800
H	3.23222900	-0.01197400	1.87655800
H	2.32839000	-1.34336700	1.12203300
O	3.35487300	-0.05539000	-0.14042500

Ester product of rxn between 34 and i

C	-0.90839600	0.64444800	-0.01649800
O	-1.47249900	1.14088700	0.94024500
O	-1.47287700	0.41440900	-1.21479200
C	-2.87269700	0.77462600	-1.45214300
C	-3.08540300	2.28108700	-1.35883300
H	-4.08416500	2.52902500	-1.73611700
H	-3.00304900	2.63138200	-0.32824200
H	-2.34990600	2.81125100	-1.97380300
C	-3.81533600	-0.04382200	-0.57745200
H	-3.57503500	-1.11028800	-0.64852600
H	-3.75603100	0.26278400	0.46853800
H	-4.84485800	0.09531100	-0.92695900
H	-2.99773700	0.46350600	-2.49353000
C	0.54801900	0.21954900	-0.00526300
C	1.45921800	1.46179900	0.07957800
C	0.83791600	-0.70136600	1.19378700
H	0.76243600	-0.31499100	-0.93732500
C	2.91721400	1.03282000	0.23803700
H	1.16087000	2.06460400	0.94486400
H	1.34899700	2.08288000	-0.81804500
C	2.33460900	-0.99350900	1.28374300
H	0.50433100	-0.20461600	2.11116300
H	0.28150500	-1.64255100	1.10189200
H	3.56525300	1.90193000	0.38780000
H	3.25637800	0.50928700	-0.67330500
H	2.56613400	-1.57365000	2.18233100
H	2.65708300	-1.58276300	0.40708300
O	3.10474200	0.19838300	1.37336300

Ester product of rxn between 34 and j

C	-1.01806800	0.26726300	-0.15777000
O	-1.82493200	0.14632200	0.74129100
O	-1.35924100	0.69702900	-1.39626200
C	-2.73941200	1.03995800	-1.55862800
C	-3.12565400	1.23778200	-3.02568100
C	-3.74194000	-0.11991400	-1.58681600
H	-2.99715600	1.85365900	-0.87867600
H	-2.33358300	0.94208900	-3.72565200
H	-3.51618000	2.22298600	-3.30452800
H	-3.27120300	-1.11052500	-1.55706200
H	-4.55957400	-0.07738300	-0.86007900
O	-4.17146800	0.24293000	-2.92307500
C	0.46852400	0.00501100	-0.06113700

C	1.23767700	1.34357300	0.03124300
C	0.81947700	-0.87565100	1.14415300
H	0.77671600	-0.49460000	-0.98914900
C	2.72805200	1.06956900	0.23164900
H	0.86019900	1.92049500	0.88465800
H	1.08321800	1.93948400	-0.87492800
C	2.33675700	-1.00317000	1.27461800
H	0.41004500	-0.42331900	2.05344800
H	0.36890800	-1.86999600	1.04194500
H	3.27740500	2.00273600	0.38944300
H	3.14054000	0.57883400	-0.66751500
H	2.60579700	-1.55154500	2.18229700
H	2.74570600	-1.55617300	0.41060000
O	2.97077800	0.26620800	1.37731000

Ester product of rxn between 34 and k

C	-0.92440800	0.93906800	-0.09968700
O	-1.41810100	1.70399600	0.70250000
O	-1.54842200	0.51585800	-1.22375600
C	-2.87735100	1.05100400	-1.47293100
H	-3.34847300	1.22400700	-0.50278300
C	-2.78794900	2.36890400	-2.21906200
C	-3.92652900	3.18067900	-2.28590900
C	-1.62006600	2.77752200	-2.86761500
C	-3.89681900	4.38401300	-2.98728200
H	-4.83891400	2.86524500	-1.78584100
C	-1.58954400	3.98443800	-3.57067600
H	-0.73349700	2.15423800	-2.81924300
C	-2.72593400	4.79023500	-3.63319900
H	-4.78591700	5.00724000	-3.02702000
H	-0.67436400	4.29297500	-4.06866700
H	-2.70116900	5.72957000	-4.17837400
C	-3.64150800	-0.01981300	-2.22262800
C	-4.59784800	-0.78688100	-1.55094800
C	-3.39163700	-0.27011400	-3.57720600
C	-5.29805700	-1.79204700	-2.22061100
H	-4.79421100	-0.59529300	-0.49890300
C	-4.08442800	-1.27755300	-4.24582200
H	-2.66059100	0.33277500	-4.10748900
C	-5.04097800	-2.03974300	-3.56931700
H	-6.04267000	-2.37855900	-1.68962700
H	-3.88348900	-1.46490300	-5.29699100
H	-5.58517000	-2.82055700	-4.09336900
C	0.44367300	0.30663700	0.03749500

C	1.50701100	1.36009800	0.39258600
C	0.41303700	-0.77625800	1.13800600
H	0.70091100	-0.16907600	-0.91558500
C	2.84523800	0.67887400	0.67408400
H	1.18024400	1.91122300	1.28070900
H	1.62298000	2.08350500	-0.42413800
C	1.82108500	-1.32241700	1.37007900
H	0.03767000	-0.33141600	2.06671000
H	-0.26274000	-1.59293400	0.85638200
H	3.58929700	1.40579400	1.01354400
H	3.22835200	0.20537800	-0.24715100
H	1.83195000	-2.02789800	2.20656200
H	2.16912100	-1.85750300	0.46903400
O	2.74076300	-0.29276600	1.70725700

Ester product of rxn between 34 and l

C	-0.86129100	1.26154300	-0.12321500
O	-1.41139500	2.14014900	0.51092200
O	-1.45196800	0.56438100	-1.11439200
C	-2.84148600	0.87394000	-1.40630000
C	-2.93160100	2.10612800	-2.32392700
C	-3.45489800	-0.35622400	-2.05923600
H	-3.33431100	1.07740500	-0.45159200
C	-4.19318700	2.06539100	-3.21586400
H	-2.03087100	2.13089200	-2.94852700
H	-2.92017900	3.00936500	-1.70601500
C	-4.97518000	-0.14947700	-2.25499600
H	-2.96183200	-0.52235800	-3.02531200
H	-3.25845900	-1.24194100	-1.44578700
C	-5.33938900	1.33236100	-2.50893700
H	-3.96809200	1.54782100	-4.15753500
H	-4.49080600	3.08473400	-3.48585600
H	-5.51534200	-0.51155700	-1.37228300
H	-5.30838100	-0.76547600	-3.09828700
H	-5.54448800	1.83497000	-1.55415700
H	-6.26228900	1.39732100	-3.09641900
C	0.55728400	0.77462900	0.10234800
C	1.34132500	1.69807700	1.04106000
C	0.55343100	-0.66236900	0.66963300
H	1.04998500	0.74326300	-0.87950700
C	2.72024000	1.10574400	1.32812800
H	0.78924800	1.81199800	1.98006600
H	1.44748800	2.69746500	0.60323300
C	1.98371000	-1.10175900	0.98102500

H	-0.03806100	-0.68577300	1.59373700
H	0.09269700	-1.35488100	-0.04259700
H	3.25828300	1.70606300	2.06797400
H	3.32366200	1.08695200	0.40314700
H	1.99341100	-2.08076100	1.47017800
H	2.56103400	-1.18463700	0.04302600
O	2.63409900	-0.20642200	1.87184900

Ester product of rxn between 34 and m

C	-0.93729900	0.67445900	-0.02329800
O	-1.47624100	1.20358500	0.93092600
O	-1.52209900	0.40740200	-1.20256500
C	-2.93424000	0.75076900	-1.46201300
C	-3.12653200	2.26467100	-1.36627200
H	-4.14053300	2.52516400	-1.68929400
H	-2.98364800	2.61876700	-0.34433500
H	-2.41770400	2.78139600	-2.02287100
C	-3.13693000	0.26710000	-2.89676500
H	-4.16365700	0.46870400	-3.21949300
H	-2.45209400	0.78109100	-3.57939700
H	-2.95530500	-0.81017700	-2.97232300
C	-3.84090100	-0.01487600	-0.49761200
H	-3.62187800	-1.08765400	-0.53925200
H	-3.71260200	0.33173000	0.52878500
H	-4.88752000	0.12927300	-0.78812400
C	0.51669700	0.23851200	-0.02444800
C	1.43948800	1.47009300	0.07739100
C	0.80096700	-0.70369500	1.15999500
H	0.72297200	-0.28546100	-0.96422600
C	2.89378900	1.02578600	0.22603700
H	1.14793900	2.06236200	0.95207800
H	1.33351800	2.10611200	-0.81032700
C	2.29514300	-1.01111200	1.24142800
H	0.47496000	-0.21771000	2.08590700
H	0.23542300	-1.63806900	1.05490600
H	3.54994300	1.88657600	0.38798400
H	3.22675200	0.51335700	-0.69386400
H	2.52353800	-1.60767800	2.13001600
H	2.60978300	-1.58924200	0.35455400
O	3.07632000	0.17208400	1.34788200

Ester product of rxn between 35 and a

C	-0.30438200	1.30101300	0.01837300
O	-0.14242300	2.46040500	0.34055700

O	-1.35769200	0.87416700	-0.70968600
C	-2.28592800	1.89437100	-1.11406000
H	-1.78965800	2.64309700	-1.73779100
H	-3.05965200	1.37825600	-1.68405300
H	-2.72197600	2.38744600	-0.24086300
C	0.63882900	0.15686700	0.33196300
C	1.45356200	-0.20778800	-0.93286400
C	1.57036600	0.49981700	1.50287300
H	0.02519700	-0.71517700	0.59485800
C	2.48017600	-1.30260700	-0.61883900
H	1.98513100	0.68254700	-1.29235400
H	0.78049900	-0.53914700	-1.73067000
C	2.58630600	-0.62365700	1.73218500
H	2.10351400	1.43120500	1.28292900
H	0.98658100	0.66892800	2.41513800
H	3.12044400	-1.49776400	-1.47823700
H	1.96665600	-2.23429000	-0.34729300
H	3.28854800	-0.36561200	2.52170100
H	2.06838400	-1.54604100	2.02803000
N	3.32748700	-0.90789800	0.50309500
C	4.55375600	-0.35879100	0.22172000
O	5.07781400	-0.39717500	-0.88487700
O	5.10197300	0.19540700	1.32709600
C	6.42269700	0.83541700	1.27554200
C	7.49111700	-0.19421800	0.90514700
H	7.35581700	-0.54968700	-0.11718800
H	8.48507200	0.25858000	0.99385400
H	7.44553000	-1.05097700	1.58679400
C	6.61177900	1.31839000	2.71306700
H	7.57604400	1.82691200	2.81532600
H	5.81889600	2.02034400	2.99238700
H	6.58905600	0.47525300	3.41165100
C	6.39373800	2.02038000	0.30877300
H	6.25432700	1.68574200	-0.71989300
H	5.57944200	2.70476400	0.57231100
H	7.33757000	2.57280800	0.37645600

Ester product of rxn between 35 and b

C	-0.49459700	0.14843600	0.17633800
O	-0.64042900	1.07642600	0.94650400
O	-1.34809500	-0.12560600	-0.83003900
C	-2.46942300	0.77476600	-0.98274900
C	-3.28420100	0.29811000	-2.17148500
H	-3.05613900	0.76886100	-0.05793600

C	-4.50526300	1.18923700	-2.42556400
H	-2.64232700	0.28118900	-3.06205200
H	-3.60717600	-0.73598100	-1.99346700
C	-5.33494400	0.71955700	-3.62176200
H	-4.17372100	2.22344200	-2.59193500
H	-5.13595900	1.20908100	-1.52601400
H	-4.73841700	0.72179200	-4.54241800
H	-5.70636200	-0.30142300	-3.46962800
H	-2.08975500	1.79139200	-1.13019900
H	-6.20222500	1.36972100	-3.78410800
C	0.66566100	-0.82783000	0.18216200
C	1.61965500	-0.51323300	-0.99530700
C	1.41879400	-0.80332700	1.51914400
H	0.25170100	-1.83141400	0.01435600
C	2.84394700	-1.43425700	-0.95996300
H	1.95833300	0.52812600	-0.92160900
H	1.09025100	-0.62505000	-1.94727600
C	2.64629000	-1.71910200	1.46523800
H	1.74011200	0.22101300	1.73776400
H	0.75508500	-1.11418500	2.33426200
H	3.56454200	-1.15787600	-1.72867200
H	2.53750900	-2.47523900	-1.12662800
H	3.22073400	-1.66406900	2.38727800
H	2.33262700	-2.76212300	1.32344600
N	3.50888500	-1.36017200	0.33860800
C	4.58948700	-0.52205100	0.45963300
O	5.16422800	-0.01156200	-0.49417600
O	4.94044600	-0.36819600	1.75677600
C	6.06490200	0.49545100	2.13825200
C	7.36887900	-0.04157100	1.54647800
H	7.37030200	0.04084300	0.45900300
H	8.21545800	0.52802400	1.94618300
H	7.50600800	-1.09328600	1.82171000
C	6.07252500	0.37326700	3.66166300
H	6.87746600	0.98342200	4.08421600
H	5.12114600	0.71580100	4.08238500
H	6.23011900	-0.66687200	3.96620400
C	5.78200600	1.93759400	1.71421800
H	5.76810700	2.03262900	0.62761600
H	4.81500000	2.26692300	2.11089400
H	6.55780000	2.59871000	2.11635800

Ester product of rxn between 35 and c

C	-0.72197400	-0.33623200	0.04067200

O	-1.28379100	-0.42439500	1.11423700
O	-1.17174300	0.42805700	-0.97400300
C	-2.37199700	1.19652800	-0.70757100
H	-3.18488900	0.50630900	-0.45858100
H	-2.19208500	1.83091500	0.16658800
C	-2.69113100	2.00059300	-1.93181500
C	-3.43221100	1.35329800	-3.07465500
C	-4.13273700	2.36111600	-2.20055100
H	-1.94335300	2.74648300	-2.19154000
H	-3.70635300	0.30672000	-2.96766400
H	-3.16426500	1.64388400	-4.08640100
H	-4.34229900	3.34565600	-2.60847700
H	-4.88502800	1.99848000	-1.50444300
C	0.57512500	-1.02713100	-0.33490400
C	1.71866400	0.01177400	-0.41375500
C	0.92407700	-2.15182800	0.64853500
H	0.44273500	-1.44724400	-1.34143800
C	3.05138500	-0.68033800	-0.71924900
H	1.80243100	0.53696400	0.54673400
H	1.49609800	0.75734600	-1.18412000
C	2.28595000	-2.76872900	0.31076100
H	0.95289500	-1.74857600	1.66659900
H	0.14805100	-2.92547000	0.63120000
H	3.87459000	0.03085500	-0.71421000
H	3.00828100	-1.14449300	-1.71377500
H	2.58085100	-3.50428000	1.05778000
H	2.24767400	-3.26438700	-0.66843500
N	3.32101300	-1.73739500	0.25400500
C	4.11341100	-1.55640600	1.35974200
O	4.16346200	-2.34315700	2.29700200
O	4.84024300	-0.41937700	1.25529100
C	5.76312800	-0.01322400	2.32251200
C	4.98714700	0.23151700	3.61762200
H	4.56717300	-0.69747700	4.00539300
H	5.65574200	0.65982700	4.37271900
H	4.17147500	0.94235000	3.44287500
C	6.33760100	1.29597200	1.78193600
H	7.05050900	1.71788300	2.49783700
H	6.85843800	1.12801700	0.83320100
H	5.54118000	2.02878600	1.61395800
C	6.86788800	-1.05693300	2.49331900
H	6.46696000	-1.99309500	2.88351800
H	7.35571400	-1.25402300	1.53218300
H	7.62565900	-0.67845400	3.18854400

Ester product of rxn between 35 and d

C	-0.12955600	1.23848200	-0.80934200
O	-0.16312200	2.43876400	-0.62994900
O	-1.22204700	0.44749700	-0.75370100
C	-2.46817900	1.11132700	-0.42291200
H	-2.74889600	1.78209700	-1.24104500
H	-2.30462400	1.72171500	0.47259000
C	-3.49846900	0.05405900	-0.19100800
H	-3.25325300	-0.68952100	0.56641400
C	-4.66835400	0.01143600	-0.82711500
H	-4.93078700	0.74252800	-1.58944600
H	-5.41180600	-0.74988100	-0.60600200
C	1.11548500	0.42005700	-1.09203100
C	2.30465300	1.31018900	-1.47518700
C	1.45879900	-0.45973900	0.13258500
H	0.87907200	-0.25504900	-1.92616400
C	3.57144000	0.46983700	-1.67073300
H	2.47806100	2.04708500	-0.68329200
H	2.08156600	1.86864200	-2.39130700
C	2.75154300	-1.24392800	-0.11845700
H	1.59497100	0.17886100	1.01507800
H	0.63548600	-1.15022000	0.34265900
H	4.43970800	1.10188300	-1.85147800
H	3.45027900	-0.20640400	-2.52802100
H	3.04108900	-1.81628600	0.76001300
H	2.60058100	-1.94760800	-0.94873400
N	3.84098200	-0.34312500	-0.48685500
C	4.80881100	0.07733600	0.38897400
O	5.60166000	0.97872000	0.14563300
O	4.78013300	-0.65022400	1.53021300
C	5.74514500	-0.40131100	2.60848700
C	7.16797700	-0.66555700	2.11372100
H	7.46448700	0.06608300	1.36117300
H	7.86675800	-0.60988400	2.95599700
H	7.23807700	-1.66898500	1.67910300
C	5.33883700	-1.43403600	3.65932000
H	5.98720600	-1.35292100	4.53785500
H	4.30312100	-1.27484700	3.97795600
H	5.42507600	-2.44954300	3.25834300
C	5.57064400	1.01789600	3.15133400
H	4.52845500	1.18682000	3.44511600
H	6.20018600	1.15216800	4.03811000
H	5.85155400	1.76185200	2.40480900

Ester product of rxn between 35 and e

C	-0.50698500	0.28215000	0.50456700
O	-0.65585300	0.85058200	1.56751200
O	-1.35140100	0.42015000	-0.53824600
C	-2.45236100	1.34631500	-0.34349200
H	-2.04577000	2.28674400	0.03987900
C	0.64340800	-0.63939800	0.14847800
C	1.56855200	0.04772800	-0.88452700
C	1.42922700	-1.06581900	1.39557900
H	0.21283500	-1.52812300	-0.33330900
C	2.78612500	-0.83228600	-1.18955200
H	1.91779800	1.00680300	-0.48048700
H	1.01306500	0.25622200	-1.80467200
C	2.64678700	-1.91014600	1.00506800
H	1.76689200	-0.17531400	1.93722200
H	0.78277900	-1.63126200	2.07647300
H	3.48909700	-0.31649700	-1.84250900
H	2.46646000	-1.75926100	-1.68320800
H	3.24424600	-2.16771400	1.87675000
H	2.32148100	-2.84503800	0.52916700
N	3.48397600	-1.19296200	0.04231500
C	4.56578600	-0.43255600	0.41216700
O	5.12447900	0.36250800	-0.33372800
O	4.93621100	-0.70292100	1.68454400
C	6.07122400	-0.00810000	2.30522800
C	7.36273900	-0.33338300	1.55381200
H	7.34552300	0.08285100	0.54582200
H	8.21857700	0.08528200	2.09516500
H	7.50018900	-1.41839800	1.48675100
C	6.09687700	-0.60889900	3.71026200
H	6.91255100	-0.16903500	4.29338600
H	5.15397100	-0.41378100	4.23212500
H	6.24883500	-1.69252100	3.66506300
C	5.79396800	1.49495500	2.36649100
H	4.83120700	1.68339800	2.85495100
H	6.57583700	1.98971200	2.95346200
H	5.77363100	1.93335400	1.36787200
H	-3.12747500	0.93788300	0.41401300
C	-3.14273100	1.52784200	-1.66489900
C	-2.50156600	2.21686600	-2.70298200
C	-4.42342300	1.01070400	-1.88016300
C	-3.13097800	2.38397800	-3.93476000
H	-1.50463900	2.61912400	-2.54107800

C	-5.05970000	1.18243700	-3.11133400
H	-4.92443600	0.47242200	-1.07945400
C	-4.41372800	1.86784700	-4.14048400
H	-2.62531800	2.92002300	-4.73308700
H	-6.05666900	0.77930400	-3.26576100
H	-4.90681600	2.00199200	-5.09926300

Ester product of rxn between 35 and f

C	-0.44207600	0.57945600	0.36840900
O	-0.59587200	1.22652300	1.38346200
O	-1.23441700	0.71264600	-0.72012400
C	-2.26903500	1.71694200	-0.60218200
H	-2.92466300	1.46343200	0.23798400
H	-1.80869800	2.68526600	-0.37736700
C	-3.00922900	1.76279100	-1.85521500
C	-3.63858300	1.82330400	-2.88265200
H	-4.19285700	1.87242500	-3.79433100
C	0.64837600	-0.44207800	0.11863000
C	1.66652000	0.11597200	-0.90443600
C	1.34620900	-0.85103200	1.42285300
H	0.17302400	-1.32327000	-0.33385400
C	2.82029100	-0.87217200	-1.10922700
H	2.07479100	1.06450600	-0.53269900
H	1.16839100	0.31782300	-1.85809100
C	2.50717200	-1.80908100	1.13338300
H	1.73155500	0.04219700	1.92692600
H	0.63058800	-1.32401900	2.10491000
H	3.58898800	-0.44846700	-1.75422900
H	2.44716300	-1.79575300	-1.57118500
H	3.04763100	-2.05888600	2.04356100
H	2.12500000	-2.74280400	0.69900100
N	3.43555400	-1.21940500	0.16883900
C	4.58037400	-0.55625300	0.53509600
O	5.23980400	0.13536300	-0.23125200
O	4.88468800	-0.79382800	1.83134700
C	6.08026700	-0.20416000	2.44737900
C	7.33996300	-0.71678900	1.74813000
H	7.39795900	-0.34897000	0.72288300
H	8.22716500	-0.37962800	2.29583100
H	7.34611600	-1.81242000	1.73210800
C	6.00550300	-0.72982100	3.88051000
H	6.85376400	-0.35638700	4.46344900
H	5.08041300	-0.40019300	4.36524800
H	6.03166100	-1.82461600	3.89340500

C	5.98114400	1.32200300	2.42747500
H	6.02481000	1.70572100	1.40744900
H	5.04185100	1.64695900	2.88907500
H	6.80898800	1.75164700	3.00253000

Ester product of rxn between 35 and g

C	-0.28191700	1.16800000	-0.15873000
O	-0.62168300	1.53303500	0.94677700
O	-1.15677400	0.73036000	-1.09352000
C	-2.53264100	0.68266300	-0.68674600
H	-2.62236100	0.12249300	0.25134500
H	-2.89803900	1.69875900	-0.49375400
C	-3.28349800	0.02995600	-1.79583900
C	-2.92263800	-0.53006900	-2.98358100
C	-4.13870000	-1.00038800	-3.58990600
H	-1.91962200	-0.59904300	-3.37748800
C	-5.14044400	-0.69001500	-2.72490700
H	-4.24242000	-1.50073400	-4.54269200
H	-6.21016600	-0.83465000	-2.73159500
O	-4.63444900	-0.05850500	-1.62167700
C	1.13839700	1.10710100	-0.68190000
C	2.11203400	1.84173000	0.24894800
C	1.56928100	-0.36518300	-0.88369600
H	1.14167900	1.59043000	-1.66895400
C	3.55344300	1.69185200	-0.25006700
H	2.03384900	1.42644400	1.25987200
H	1.84725500	2.90304900	0.31469700
C	3.02942300	-0.43941000	-1.34344700
H	1.46868300	-0.90807600	0.06480600
H	0.91753900	-0.85156800	-1.61646200
H	4.25771100	2.15218200	0.43919000
H	3.66593000	2.18210700	-1.22680400
H	3.37109700	-1.47219500	-1.39793000
H	3.13482700	0.01572100	-2.33733300
N	3.89939500	0.28060700	-0.41839200
C	4.59107000	-0.44597500	0.51827800
O	4.69029000	-1.66678000	0.49285000
O	5.15863800	0.36500900	1.43975600
C	5.96944300	-0.18588000	2.53418500
C	5.11202300	-1.09759800	3.41288600
H	4.80610000	-1.99233700	2.86934200
H	5.68373300	-1.39946700	4.29756100
H	4.21633300	-0.56505600	3.75193400
C	6.38307800	1.06956100	3.30138300

H	7.00395700	0.79848400	4.16148100
H	6.95805100	1.74459700	2.65864900
H	5.50171100	1.60722800	3.66682500
C	7.19583500	-0.90402100	1.96958100
H	6.90687200	-1.79835900	1.41614600
H	7.75152700	-0.23763900	1.30038600
H	7.86177000	-1.19487900	2.78988300

Ester product of rxn between 35 and h

C	-1.01583700	-0.79557100	0.30976700
O	-1.43119200	-0.47049600	1.40430600
O	-1.55343700	-0.36753900	-0.84957200
C	-2.66796200	0.56183900	-0.72550000
H	-3.47302600	0.06458300	-0.17753800
H	-2.33417400	1.41733200	-0.13121300
C	-3.09479200	0.96486500	-2.10388800
C	-4.15277200	0.32009500	-2.74632300
C	-2.42010900	1.98446600	-2.79326400
C	-4.54477600	0.67164400	-4.03981300
H	-4.68771000	-0.47377700	-2.23072300
C	-2.79295200	2.34670600	-4.07938100
H	-1.59247200	2.49938700	-2.31161500
C	-3.86097900	1.69113800	-4.71296400
H	-5.37381600	0.15238700	-4.50552900
H	-2.27758800	3.13742300	-4.61536400
O	-4.15578500	2.11882400	-5.97018400
C	-5.23080800	1.48923000	-6.65463100
H	-5.29351200	1.97879600	-7.62845800
H	-5.04315500	0.41764100	-6.80047200
H	-6.18093800	1.62266700	-6.12151400
C	0.18283800	-1.68816900	0.05169600
C	1.39103700	-0.81978300	-0.37209300
C	0.52952400	-2.54255500	1.27782100
H	-0.06748700	-2.34171800	-0.79445300
C	2.63470200	-1.69352800	-0.56871900
H	1.59722200	-0.07629400	0.40877200
H	1.15965800	-0.28034600	-1.29678400
C	1.80413700	-3.35739400	1.03109300
H	0.67838100	-1.88989900	2.14483300
H	-0.30174700	-3.21504900	1.51929500
H	3.50754200	-1.08852200	-0.80414900
H	2.46880800	-2.39066900	-1.40142800
H	2.10823300	-3.89489600	1.92817100
H	1.63860500	-4.08836400	0.22802900

N	2.90577000	-2.48501400	0.62942200
C	3.83943900	-2.13739800	1.57199100
O	3.93518300	-2.67800700	2.66695100
O	4.64245800	-1.14664900	1.11766900
C	5.74247300	-0.62916300	1.94085300
C	5.18995500	-0.00806200	3.22474900
H	4.75063400	-0.76810200	3.87183000
H	5.99810500	0.49480500	3.76755900
H	4.42447400	0.73860800	2.98520500
C	6.34956500	0.44632100	1.04056900
H	7.20020500	0.92091900	1.54064700
H	6.70048100	0.01054900	0.09908800
H	5.60929600	1.21954200	0.80888400
C	6.75813400	-1.73799900	2.21934500
H	6.32723400	-2.51442000	2.85274900
H	7.09172000	-2.19223600	1.27956600
H	7.63474800	-1.31614200	2.72371600

Ester product of rxn between 35 and i

C	-0.31020700	0.88159900	-0.41485900
O	-0.24487000	2.08418900	-0.24950800
O	-1.43632400	0.14880100	-0.33342900
C	-2.70647300	0.78198400	0.02454900
C	-3.15713300	1.77348000	-1.04169500
H	-4.19376800	2.07023800	-0.84462900
H	-2.53087700	2.66756100	-1.04412200
H	-3.11986600	1.31178000	-2.03440800
C	-2.66306100	1.35857900	1.43478200
H	-2.28311800	0.61289600	2.14182100
H	-2.02987500	2.24668400	1.48354000
H	-3.67776200	1.63369600	1.74460700
H	-3.38254600	-0.07824700	0.01564900
C	0.88232000	-0.01528400	-0.69739200
C	2.04914900	0.76699000	-1.31415700
C	1.32135300	-0.70756600	0.61447400
H	0.55344500	-0.79827100	-1.39305300
C	3.27793900	-0.13249400	-1.48533200
H	2.30402700	1.61024700	-0.66332400
H	1.75553600	1.18457100	-2.28432400
C	2.57184300	-1.56202300	0.37956100
H	1.54805700	0.05584200	1.36988400
H	0.50742900	-1.33053400	1.00093600
H	4.13732900	0.43664700	-1.83695100
H	3.06774100	-0.92862500	-2.21268000

H	2.92902200	-2.00061700	1.30876000
H	2.33602300	-2.38051500	-0.31450500
N	3.63908300	-0.76229900	-0.21707500
C	4.69057800	-0.24474600	0.49449600
O	5.47352700	0.58380400	0.04623100
O	4.75500900	-0.78988600	1.73219600
C	5.82079600	-0.41134800	2.66831500
C	7.18574200	-0.79545200	2.09513900
H	7.42313800	-0.19987500	1.21278100
H	7.96150800	-0.63224800	2.85167700
H	7.19686000	-1.85597400	1.81958100
C	5.49535400	-1.25694600	3.89923600
H	6.22709200	-1.06603200	4.69099600
H	4.49884000	-1.01395500	4.28315400
H	5.52068500	-2.32405700	3.65418700
C	5.72512300	1.07912900	2.99787300
H	5.94954000	1.69040100	2.12297400
H	4.71797000	1.32547200	3.35268500
H	6.43616100	1.32630300	3.79419000

Ester product of rxn between 35 and j

C	-0.49604900	-0.19824500	-0.25267200
O	-1.02981900	-1.01628700	0.46805900
O	-1.14175200	0.89499400	-0.72513400
C	-2.49348400	1.03948600	-0.27734500
C	-3.25649000	2.11752700	-1.04954200
C	-3.52645900	0.07148700	-0.86665800
H	-2.51202500	1.12321200	0.81054500
H	-2.70646900	2.49602800	-1.92079700
H	-3.64986100	2.95814600	-0.46696400
H	-3.12061000	-0.58257900	-1.64865000
H	-4.10584700	-0.52163600	-0.15193100
O	-4.29036200	1.17406700	-1.41677200
C	0.94516100	-0.22452000	-0.71419900
C	1.76066100	0.83607000	0.06348300
C	1.56569400	-1.61838300	-0.55151900
H	0.95558900	0.06250000	-1.77447600
C	3.23761200	0.77418800	-0.34159900
H	1.67733600	0.64241100	1.14065200
H	1.35926700	1.83644800	-0.12973300
C	3.05013600	-1.59905700	-0.93300700
H	1.46381700	-1.94253900	0.48982600
H	1.03004200	-2.34696500	-1.17070500
H	3.83499100	1.46869100	0.24495100

H	3.34166800	1.04551800	-1.40107300
H	3.52035500	-2.56089000	-0.73376400
H	3.16505000	-1.37115900	-2.00144500
N	3.76524800	-0.57736800	-0.17150600
C	4.50482600	-0.97185100	0.91476400
O	4.77992900	-2.13950000	1.16113300
O	4.90005100	0.09531200	1.64697300
C	5.73917600	-0.07787500	2.84045100
C	4.99601000	-0.90720700	3.88878200
H	4.85397100	-1.93403900	3.54991100
H	5.56978100	-0.91889500	4.82227600
H	4.01563200	-0.46440300	4.09797600
C	5.92497700	1.36009700	3.32363700
H	6.54328000	1.37738700	4.22706100
H	6.41708300	1.96633200	2.55572900
H	4.95775000	1.81742100	3.55799400
C	7.08294900	-0.69627600	2.45266500
H	7.56133700	-0.10314800	1.66526400
H	7.74827700	-0.70593600	3.32336200
H	6.95581300	-1.71915200	2.09588600

Ester product of rxn between 35 and k

C	-0.46699400	0.07409000	-0.36353700
O	-0.75492700	0.49917400	0.73609200
O	-1.29607900	0.11777100	-1.43199100
C	-2.57930500	0.77305000	-1.23042000
H	-2.87321800	0.60618200	-0.19218800
C	-2.45262100	2.26490800	-1.47398200
C	-3.45716000	3.11271300	-0.99320100
C	-1.38691700	2.81036000	-2.19478900
C	-3.39747700	4.48472300	-1.22864000
H	-4.28903900	2.69286800	-0.43308400
C	-1.32629100	4.18586700	-2.43062300
H	-0.60297200	2.15959300	-2.56733800
C	-2.33011400	5.02631600	-1.94984100
H	-4.18122100	5.13234600	-0.84556600
H	-0.49137800	4.59869000	-2.99033600
H	-2.28129100	6.09620500	-2.13186200
C	-3.56896800	0.09141200	-2.15108600
C	-4.51508500	-0.78972200	-1.61943000
C	-3.54521700	0.31791500	-3.53249400
C	-5.42771800	-1.43812800	-2.45366500
H	-4.53644100	-0.96790200	-0.54705100
C	-4.45014700	-0.33401700	-4.36779700

H	-2.82346600	1.01364300	-3.94965500
C	-5.39537500	-1.21209200	-3.82995200
H	-6.16136800	-2.11724600	-2.02836800
H	-4.42375400	-0.15226600	-5.43864700
H	-6.10501200	-1.71408400	-4.48173500
C	0.85091300	-0.58505200	-0.71081800
C	2.02077400	0.34449900	-0.33053400
C	0.97117400	-1.93711000	0.02603400
H	0.87150600	-0.76859600	-1.79099800
C	3.36458000	-0.35724800	-0.55172100
H	1.92683300	0.62455100	0.72457800
H	1.97991000	1.26725600	-0.92219600
C	2.35149300	-2.56081300	-0.20647700
H	0.82580400	-1.77545400	1.10037200
H	0.18779600	-2.62361200	-0.31679100
H	4.19319600	0.26892600	-0.22815500
H	3.50268600	-0.57808100	-1.61865200
H	2.47377300	-3.47080600	0.37946700
H	2.48197400	-2.80876000	-1.26791200
N	3.40873900	-1.62652100	0.17360100
C	3.95906700	-1.75482800	1.42535200
O	3.79057300	-2.73190000	2.14428100
O	4.72346000	-0.68172200	1.73116800
C	5.41259900	-0.58926800	3.02489300
C	4.38925300	-0.58191000	4.16168400
H	3.87547500	-1.54134500	4.23528800
H	4.89613600	-0.37915000	5.11177600
H	3.64521800	0.20585400	3.99802700
C	6.11919600	0.76227000	2.92634400
H	6.67929200	0.96200700	3.84570500
H	6.82044600	0.77205700	2.08513500
H	5.39322200	1.56927700	2.78108100
C	6.43019300	-1.72235700	3.16292200
H	5.93291700	-2.69068100	3.23031500
H	7.10750300	-1.73094000	2.30163400
H	7.03034600	-1.56859000	4.06679000

Ester product of rxn between 35 and l

C	-0.12694000	1.19370800	-0.67616400
O	-0.28219500	2.31376400	-0.23068500
O	-1.13511000	0.39621400	-1.08097600
C	-2.48538100	0.91817600	-0.94614800
C	-2.81115000	1.86223800	-2.11763300
C	-3.43192000	-0.27253200	-0.90710300

H	-2.52738400	1.46251600	0.00150600
C	-4.31913400	1.85966000	-2.45449800
H	-2.23176800	1.53437300	-2.98879100
H	-2.47019000	2.87087300	-1.86541000
C	-4.87216600	0.20579800	-0.61028000
H	-3.38915300	-0.78530400	-1.87618700
H	-3.09755700	-0.99160000	-0.15171100
C	-5.16002100	1.60826100	-1.19737800
H	-4.53629000	1.07473100	-3.19078100
H	-4.59644600	2.80999900	-2.92408100
H	-5.04085200	0.22203300	0.47297800
H	-5.57714100	-0.52451000	-1.02408800
H	-4.92157300	2.38020200	-0.45349100
H	-6.22849500	1.71164800	-1.41801500
C	1.20722100	0.48451600	-0.80602200
C	2.37229300	1.48024000	-0.89120700
C	1.39453300	-0.47964800	0.38904700
H	1.17328800	-0.12244200	-1.72012100
C	3.71730900	0.74573900	-0.90802900
H	2.33707400	2.15360900	-0.02814900
H	2.27541500	2.10100900	-1.78963000
C	2.77020000	-1.15211100	0.32372200
H	1.31640800	0.08358200	1.32785300
H	0.60380800	-1.23784400	0.38635700
H	4.54992200	1.44699900	-0.87360700
H	3.80991900	0.14154400	-1.82064300
H	2.94148000	-1.78514200	1.19158900
H	2.83169500	-1.78082500	-0.57502800
N	3.83119200	-0.15029500	0.24105400
C	4.55767400	0.27171000	1.32553600
O	5.29785900	1.24747800	1.31060400
O	4.36388200	-0.54531300	2.38720000
C	5.06694200	-0.31470600	3.65546900
C	6.57758700	-0.43398200	3.44967900
H	6.95257400	0.37175200	2.81739900
H	7.08556300	-0.38816300	4.41957400
H	6.82258700	-1.39432100	2.98222400
C	4.54985200	-1.45543900	4.53154900
H	5.00286200	-1.40100300	5.52684600
H	3.46194400	-1.39532400	4.64297600
H	4.79903800	-2.42636600	4.09035500
C	4.66248100	1.03968000	4.23964700
H	3.57155400	1.10839400	4.31768100
H	5.08294400	1.14735300	5.24585200

H	5.02322700	1.85968600	3.61762100

Ester product of rxn between 35 and m

C	-0.60428900	0.20559000	0.24575600
O	-0.81445200	0.72280800	1.32599200
O	-1.37957600	0.33328900	-0.84509300
C	-2.57731300	1.19344400	-0.85250400
C	-2.17744500	2.64217100	-0.57061000
H	-3.04477700	3.29553000	-0.71670300
H	-1.81742500	2.76432500	0.45208600
H	-1.39098100	2.96169100	-1.26339200
C	-3.08925000	1.04156300	-2.28368200
H	-3.99277000	1.64357400	-2.42538300
H	-2.33396700	1.37614700	-3.00258900
H	-3.33216600	-0.00414200	-2.49974100
C	-3.60295600	0.66487200	0.15102700
H	-3.80759500	-0.39506500	-0.03640600
H	-3.25021800	0.77971200	1.17700800
H	-4.54263500	1.21717200	0.03893200
C	0.60337000	-0.66059300	-0.07299500
C	1.56608800	0.10604000	-1.01006300
C	1.32719400	-1.11646600	1.20043400
H	0.23972800	-1.54033800	-0.62158400
C	2.82700100	-0.72073800	-1.28373100
H	1.85823600	1.05270500	-0.53721900
H	1.06059900	0.34589500	-1.95103100
C	2.59396900	-1.90477400	0.85054700
H	1.59939600	-0.24033800	1.79909400
H	0.66169800	-1.73284700	1.81572500
H	3.55224900	-0.15229700	-1.86458200
H	2.56624200	-1.62890800	-1.84347600
H	3.14863900	-2.17802600	1.74536600
H	2.32660100	-2.83012700	0.32179300
N	3.46137600	-1.12487900	-0.03227400
C	4.54344600	-0.40665900	0.40835000
O	5.14339600	0.41069700	-0.27971400
O	4.86467000	-0.74266600	1.67914000
C	6.01153500	-0.12495800	2.35621800
C	7.30591300	-0.47338100	1.62009700
H	7.32929300	-0.01554800	0.63036700
H	8.16561900	-0.11386200	2.19669200
H	7.39988500	-1.55956400	1.51033500
C	5.97785200	-0.78742200	3.73321800
H	6.79804600	-0.41039800	4.35285200

H	5.03248300	-0.57349300	4.24311600
H	6.08341200	-1.87375500	3.64294900
C	5.79842300	1.38462800	2.47950400
H	5.82331400	1.86725100	1.50169900
H	4.83222400	1.59389500	2.95233900
H	6.58538300	1.81707000	3.10756500

Ester product of rxn between 36 and a

C	-1.05518300	0.78345200	0.25067100
O	-1.92965200	1.44933800	0.76569600
O	-1.18580000	0.16736800	-0.93924600
C	-2.44073600	0.37746700	-1.60919700
H	-2.59350700	1.44090600	-1.81304000
H	-2.37178200	-0.18205600	-2.54291400
H	-3.27006700	0.00436300	-1.00211100
C	0.33470000	0.58148600	0.82475900
H	0.70346400	-0.41222000	0.55820200
H	0.25072100	0.64815800	1.91327100
C	1.27370400	1.65082100	0.29386800
C	1.09967000	2.98830800	0.67309100
C	2.29976700	1.32613600	-0.59886700
C	1.94412500	3.97991700	0.17559500
H	0.29451500	3.24804600	1.35493200
C	3.14756000	2.31778700	-1.09624500
H	2.43592100	0.29189200	-0.90425100
C	2.97216900	3.64717300	-0.71004700
H	1.79987300	5.01306300	0.47967100
H	3.94372200	2.05021400	-1.78559900
H	3.63175400	4.41952400	-1.09575700

Ester product of rxn between 36 and b

C	0.22317200	0.94698600	-1.52676000
O	0.36900800	1.99330300	-2.12550900
O	-0.94139200	0.27941000	-1.45067900
C	-2.07647300	0.90332600	-2.09532700
C	-3.29293600	0.03446000	-1.83210000
H	-2.20067000	1.91232400	-1.68852800
C	-4.55788200	0.61199700	-2.47692000
H	-3.10456000	-0.97568500	-2.21866000
H	-3.43628500	-0.06133900	-0.74796900
C	-5.79501300	-0.24920600	-2.21635400
H	-4.40172400	0.71291800	-3.55983400
H	-4.73022600	1.62754000	-2.09466100
H	-5.66403200	-1.26224300	-2.61657300

H	-5.99423700	-0.34054000	-1.14143400
H	-1.86681300	0.99782500	-3.16627900
H	-6.68653800	0.18236100	-2.68574000
C	1.30539300	0.25170500	-0.72035600
H	2.27018900	0.55618300	-1.13524200
H	1.20090300	-0.83167300	-0.82304300
C	1.19597400	0.65404700	0.73955500
C	0.74674700	-0.25364300	1.70378100
C	1.51073800	1.96189400	1.13132800
C	0.62521300	0.13286500	3.03983300
H	0.49192600	-1.26753600	1.40635600
C	1.38794800	2.35008500	2.46486400
H	1.84693800	2.67563900	0.38398200
C	0.94574100	1.43559700	3.42400600
H	0.27902600	-0.58442400	3.77902200
H	1.63793200	3.36681400	2.75549000
H	0.85233600	1.73712400	4.46358200

Ester product of rxn between 36 and c

C	-2.02886600	0.66973200	0.96640800
O	-2.99451900	1.23153500	1.44083300
O	-1.83156900	0.49452700	-0.35220400
C	-2.82204000	1.08743300	-1.22973400
H	-3.78980400	0.60924400	-1.04368200
H	-2.91723400	2.14852500	-0.97805300
C	-2.36865900	0.88097600	-2.64349000
C	-2.63503100	-0.43655500	-3.32708000
C	-3.41228200	0.78958900	-3.73074600
H	-1.43968600	1.38665400	-2.89690500
H	-3.15228100	-1.20428900	-2.75703800
H	-1.88118100	-0.81872200	-4.00941300
H	-3.19028100	1.25364500	-4.68730300
H	-4.45760800	0.85124800	-3.43836400
C	-0.86268900	0.11418500	1.76668900
H	-1.20569400	-0.02785900	2.79393400
H	-0.57256700	-0.85556300	1.35125900
C	0.30542600	1.08115900	1.71612600
C	0.39854700	2.12382000	2.64550500
C	1.27332300	0.98012200	0.70996400
C	1.44717300	3.04203100	2.57835400
H	-0.35563900	2.21489800	3.42297100
C	2.32283200	1.89651600	0.64183600
H	1.19997000	0.18144900	-0.02308100
C	2.41309100	2.93014200	1.57655700

H	1.50928300	3.84399800	3.30888100
H	3.07060400	1.80286000	-0.14106100
H	3.23148000	3.64288800	1.52511000

Ester product of rxn between 36 and d

C	-1.01611000	2.29615400	-0.42270000
O	-1.85558700	3.14575500	-0.20958200
O	-1.28664400	1.08643000	-0.94883000
C	-2.68707800	0.80422200	-1.19978700
H	-3.04364700	1.44058400	-2.01586900
H	-3.25364900	1.06089200	-0.29749600
C	-2.80371300	-0.64789700	-1.53224400
H	-2.40270600	-1.33707700	-0.79007400
C	-3.38489800	-1.10704000	-2.63972100
H	-3.78800400	-0.43418100	-3.39426500
H	-3.48790200	-2.17183400	-2.83180200
C	0.46070500	2.42365100	-0.09154900
H	1.05129700	1.99787800	-0.90785700
H	0.68776300	3.48900300	-0.00696000
C	0.75949200	1.70187200	1.20934200
C	1.24128000	0.38821400	1.20556400
C	0.50475500	2.32893800	2.43509900
C	1.47769000	-0.28311400	2.40577200
H	1.43096600	-0.10856500	0.25788600
C	0.73906700	1.65874300	3.63596900
H	0.12023600	3.34553400	2.44491700
C	1.22757500	0.35067700	3.62447700
H	1.85757400	-1.30100800	2.38847900
H	0.54111900	2.15881100	4.58009600
H	1.41343200	-0.17086600	4.55926700

Ester product of rxn between 36 and e

C	-2.24489100	0.46438900	2.02751200
O	-3.04273400	1.25885200	1.57553900
O	-2.53285000	-0.81591700	2.34512300
C	-3.74885300	-1.37428500	1.76740300
H	-4.08119300	-2.12639600	2.48475200
H	-4.49623300	-0.58330300	1.68948500
C	-3.40768900	-1.98237200	0.43343200
C	-3.08660600	-3.34072900	0.33698300
C	-3.30763400	-1.17408700	-0.70743700
C	-2.67377500	-3.88748800	-0.87869700
H	-3.15503000	-3.97028600	1.22101300
C	-2.88666400	-1.71698800	-1.92020800

H	-3.54356300	-0.11715500	-0.63476700
C	-2.56912900	-3.07428100	-2.00851100
H	-2.43039700	-4.94454800	-0.94242800
H	-2.80166400	-1.07929200	-2.79538700
H	-2.24180400	-3.49633300	-2.95471200
C	-0.76182800	0.73176700	2.20405800
H	-0.61565300	1.80079900	2.37230100
H	-0.37903100	0.17098400	3.06107300
C	-0.09334000	0.28391200	0.91435000
C	0.15650600	1.20438700	-0.10925900
C	0.17258300	-1.07268000	0.68884500
C	0.67728900	0.77955000	-1.33244200
H	-0.06527200	2.25611300	0.05178100
C	0.68781300	-1.49898300	-0.53412000
H	-0.04713300	-1.79644800	1.46743400
C	0.94179300	-0.57376200	-1.54879500
H	0.87009800	1.50568000	-2.11750800
H	0.87815900	-2.55563200	-0.69859000
H	1.34040200	-0.90630900	-2.50325300

Ester product of rxn between 36 and f

C	-1.75568000	0.03388100	0.67632100
O	-2.81178400	0.35505300	1.17826100
O	-1.43303700	0.28278000	-0.61084300
C	-2.43261600	1.01750600	-1.35625300
H	-3.37488600	0.45919300	-1.34453100
H	-2.61414500	1.97848100	-0.86267200
C	-1.94629100	1.20486400	-2.71563800
C	-1.56886900	1.37559200	-3.84868100
H	-1.23044300	1.52311300	-4.85094800
C	-0.60664500	-0.64048900	1.40156300
H	-1.03121600	-1.20392000	2.23614900
H	-0.10356900	-1.33338300	0.72209500
C	0.36580700	0.41282700	1.90192700
C	1.51499400	0.73085800	1.16989400
C	0.09346600	1.11613800	3.08161500
C	2.38538600	1.72605100	1.61641900
H	1.72753700	0.19684700	0.24760600
C	0.96211500	2.11178400	3.52848100
H	-0.80407900	0.88227700	3.64804300
C	2.11150000	2.41877100	2.79711600
H	3.27724800	1.95963100	1.04143800
H	0.74132900	2.64708200	4.44787300
H	2.78987900	3.19235300	3.14590600

Ester product of rxn between 36 and g

C	-1.66703000	2.20590900	0.29818700
O	-2.66511300	2.57070100	0.88341900
O	-1.68182000	1.41325700	-0.79126300
C	-2.99685200	0.95423500	-1.21165900
H	-3.49332900	0.47270100	-0.36613700
H	-3.58867500	1.82453200	-1.50991100
C	-2.83032300	-0.00434500	-2.32426300
C	-2.87109500	-1.36689800	-2.39363600
C	-2.61770600	-1.71535400	-3.76070000
H	-3.05732800	-2.03978400	-1.56793800
C	-2.43986700	-0.53699000	-4.42077000
H	-2.57708300	-2.70704800	-4.18898900
H	-2.23126100	-0.28330400	-5.44947100
O	-2.56732500	0.51375800	-3.56502300
C	-0.24138000	2.52226600	0.71523200
H	0.36345200	2.70233800	-0.17822600
H	-0.26742200	3.43321600	1.31742900
C	0.32491100	1.36384400	1.51588600
C	0.18038700	1.33073800	2.90753500
C	0.94961800	0.28937400	0.87130400
C	0.66399200	0.24904000	3.64479900
H	-0.31361500	2.15641500	3.41301200
C	1.43379800	-0.79230400	1.60692000
H	1.05178000	0.30130300	-0.21025100
C	1.29315900	-0.81503200	2.99628100
H	0.54923200	0.23858700	4.72522900
H	1.92167600	-1.61713100	1.09474000
H	1.67243400	-1.65640300	3.56954000

Ester product of rxn between 36 and h

C	-0.69002900	3.40054700	-0.27716600
O	-1.61753900	3.78330500	0.40542700
O	-0.62741100	3.51674300	-1.61984600
C	-1.90339700	3.69652900	-2.31271000
H	-2.53822800	4.35118600	-1.71400800
H	-1.63930500	4.19241700	-3.24808000
C	-2.51036600	2.34190000	-2.53751200
C	-3.27587300	1.73063500	-1.53975900
C	-2.19637200	1.59938100	-3.68520000
C	-3.70622100	0.41010900	-1.66292900
H	-3.51658700	2.28454200	-0.63818300
C	-2.62365600	0.28626400	-3.82772000

H	-1.59810500	2.05423700	-4.47110800
C	-3.36685700	-0.32358300	-2.80585200
H	-4.28097600	-0.03737100	-0.86136800
H	-2.37959200	-0.29471100	-4.71159700
O	-3.70699200	-1.62488500	-3.01452700
C	-4.40239700	-2.30313300	-1.97698500
H	-4.53815900	-3.32833700	-2.32756200
H	-3.82282900	-2.31078600	-1.04524400
H	-5.38610300	-1.85376000	-1.78907800
C	0.49969200	2.61683600	0.24653500
H	1.38739800	2.83523400	-0.35329900
H	0.68094800	2.90323400	1.28470100
C	0.11821700	1.14848600	0.14414000
C	-0.42931800	0.48096900	1.24524300
C	0.20679800	0.47707400	-1.08204600
C	-0.86293500	-0.84068800	1.12976600
H	-0.51963200	1.00183500	2.19485000
C	-0.23178700	-0.84068400	-1.20095600
H	0.60259500	0.99734800	-1.94847400
C	-0.76578800	-1.50474400	-0.09427300
H	-1.27880300	-1.34891700	1.99558800
H	-0.16614600	-1.34579500	-2.15996400
H	-1.10296700	-2.53359500	-0.18687600

Ester product of rxn between 36 and i

C	-1.43512900	0.04320300	0.74752300
O	-2.43778800	0.38168700	1.34294600
O	-1.20931200	0.21226900	-0.56632800
C	-2.15809100	0.96805200	-1.38666600
C	-2.25910300	2.41373900	-0.91515900
H	-2.79924700	3.00302900	-1.66495600
H	-2.79109500	2.48556600	0.03562700
H	-1.26000100	2.84622400	-0.79451700
C	-3.49818900	0.25125800	-1.50196700
H	-3.35039600	-0.79466200	-1.79227100
H	-4.04783300	0.28187000	-0.55939900
H	-4.10153600	0.73663300	-2.27788400
H	-1.65994300	0.94647500	-2.36040200
C	-0.22029900	-0.58557400	1.41394500
H	0.22502100	-1.32331000	0.74057700
H	-0.56253500	-1.08893600	2.32109800
C	0.78120400	0.50376700	1.74908900
C	0.72234800	1.16172800	2.98297800
C	1.73634900	0.90877000	0.80872000

C	1.61085500	2.19646900	3.27815900
H	-0.02508200	0.86131800	3.71245200
C	2.62552500	1.94272600	1.10217200
H	1.77897000	0.41139800	-0.15634800
C	2.56580600	2.58932400	2.33870300
H	1.55631900	2.69542100	4.24185700
H	3.36577200	2.24279800	0.36543600
H	3.25941400	3.39335500	2.56846500

Ester product of rxn between 36 and j

C	-1.73882200	-0.49808200	0.34474700
O	-2.87518900	-0.53705000	0.76790000
O	-1.36919400	0.24736100	-0.72012200
C	-2.40898200	1.05386600	-1.28391100
C	-2.00912000	1.67383800	-2.62387400
C	-3.49030200	0.32611300	-2.09142600
H	-2.78503000	1.74418300	-0.52718000
H	-1.05846100	1.28838600	-3.01438300
H	-2.01506600	2.76811200	-2.68135500
H	-3.29983900	-0.74717100	-2.21639500
H	-4.52625100	0.48329100	-1.77414400
O	-3.15604900	1.08425200	-3.28094100
C	-0.54965000	-1.21781500	0.94926100
H	-0.93558800	-2.06133800	1.52696400
H	0.08937700	-1.59841700	0.14760400
C	0.22881500	-0.26385400	1.83731800
C	1.40583300	0.33969300	1.38267400
C	-0.25417300	0.05879400	3.11160900
C	2.09733400	1.24174900	2.19264600
H	1.78173100	0.10024900	0.39142000
C	0.43490400	0.96125400	3.92147700
H	-1.17400400	-0.39912900	3.46583100
C	1.61363800	1.55467900	3.46410100
H	3.01376100	1.69914700	1.82996600
H	0.05198500	1.20019100	4.90979200
H	2.15219900	2.25565000	4.09565300

Ester product of rxn between 36 and k

C	-2.36643600	0.65094200	1.25415700
O	-3.26387600	1.31023500	1.73151400
O	-2.29038200	0.29479800	-0.04710500
C	-3.26984000	0.88508600	-0.94263000
H	-4.22522700	0.92677000	-0.41509700
C	-2.85118800	2.29350900	-1.32455500

C	-3.82488000	3.18645000	-1.78540900
C	-1.51666100	2.70740100	-1.26395200
C	-3.47166100	4.47521600	-2.18126400
H	-4.86387700	2.86930600	-1.83216600
C	-1.16430600	4.00037800	-1.65601500
H	-0.75442800	2.02892900	-0.89901800
C	-2.13791200	4.88686100	-2.11660000
H	-4.23788200	5.16069300	-2.53294100
H	-0.12525000	4.31127600	-1.59374000
H	-1.86269700	5.89351700	-2.41895500
C	-3.38660800	-0.04546100	-2.13063600
C	-4.58764500	-0.71514300	-2.37719100
C	-2.30026100	-0.24813700	-2.99094700
C	-4.70754600	-1.57685000	-3.46985500
H	-5.43182300	-0.56290300	-1.70938200
C	-2.41481100	-1.11442700	-4.07565500
H	-1.36804500	0.27845600	-2.81047300
C	-3.62033600	-1.77950100	-4.31956000
H	-5.64764400	-2.08971400	-3.65319400
H	-1.56589200	-1.26749200	-4.73628400
H	-3.70982800	-2.45031600	-5.16951100
C	-1.14476400	0.15258800	2.00652600
H	-0.99350200	-0.90868900	1.78718200
H	-1.34168500	0.27128000	3.07457300
C	0.06253900	0.96010000	1.57013500
C	0.98252700	0.43864600	0.65403100
C	0.21438800	2.28069000	2.00990100
C	2.03999100	1.22197100	0.18863000
H	0.86342000	-0.58090000	0.29738000
C	1.27064800	3.06493200	1.54663700
H	-0.50800300	2.69771600	2.70662900
C	2.18585000	2.53800300	0.63259100
H	2.74864000	0.80461900	-0.52148700
H	1.37491400	4.08864400	1.89497200
H	3.00840500	3.14811600	0.27005300

Ester product of rxn between 36 and l

C	-1.65096700	0.01606500	0.86231000
O	-2.63916700	0.21783600	1.53765500
O	-1.61680400	0.08178800	-0.48124800
C	-2.82768800	0.52389300	-1.15299900
C	-2.94336300	2.05686500	-1.07378500
C	-2.74863600	0.04205300	-2.59381200
H	-3.67150400	0.05064600	-0.64335500

C	-3.70114100	2.63768700	-2.28917300
H	-1.92946800	2.47118800	-1.02728700
H	-3.44333200	2.32714900	-0.13860400
C	-4.07246300	0.35261700	-3.32942000
H	-1.90797400	0.54830500	-3.08495200
H	-2.53213500	-1.03118400	-2.61894400
C	-4.74698300	1.64371800	-2.80798500
H	-2.99312600	2.86311700	-3.09749200
H	-4.17057300	3.58879800	-2.01434600
H	-4.76465900	-0.49055700	-3.21909800
H	-3.86522900	0.44661200	-4.40173900
H	-5.43752900	1.39923700	-1.98969100
H	-5.35424300	2.09713500	-3.59964600
C	-0.26994500	-0.28925400	1.41820000
H	0.22808800	-1.01793300	0.77210600
H	-0.39804200	-0.72155300	2.41308100
C	0.53450300	0.99537900	1.48943000
C	0.50433700	1.78466700	2.64480200
C	1.26804000	1.43960400	0.38242900
C	1.20540500	2.99005900	2.69882000
H	-0.07289300	1.45220300	3.50359600
C	1.96893300	2.64451700	0.43458200
H	1.28494800	0.83869000	-0.52257100
C	1.94050500	3.42292300	1.59385400
H	1.17689600	3.59017900	3.60418000
H	2.53867600	2.97476800	-0.42979100
H	2.48842100	4.36015600	1.63560600

Ester product of rxn between 36 and m

C	-1.04857900	2.32056900	-0.22813600
O	-1.91385500	3.11062500	0.09129700
O	-1.23989800	1.14997400	-0.85645900
C	-2.59137200	0.60864700	-1.09448800
C	-3.35903000	1.51958400	-2.05242800
H	-4.30828300	1.04519100	-2.32577100
H	-3.57039700	2.48728700	-1.59511200
H	-2.78267100	1.68001700	-2.97029700
C	-2.29801100	-0.74248700	-1.74380800
H	-3.23486700	-1.25958600	-1.97588500
H	-1.73542300	-0.61113600	-2.67414300
H	-1.71010100	-1.37492000	-1.07036500
C	-3.30878800	0.42315300	0.24296500
H	-2.68522600	-0.16107900	0.92850200
H	-3.53911600	1.38264000	0.70899800

H	-4.24610500	-0.12113300	0.08346200
C	0.42898300	2.50159400	0.09145900
H	1.02804300	2.17234000	-0.76260000
H	0.60516800	3.56575100	0.26340300
C	0.77224400	1.69027200	1.32644500
C	0.65254100	2.25843900	2.59990100
C	1.14860200	0.34598300	1.21591400
C	0.91736000	1.50194900	3.74236800
H	0.34813100	3.29757600	2.69388000
C	1.41380200	-0.41159700	2.35660400
H	1.23005900	-0.10576000	0.23123800
C	1.29955500	0.16462300	3.62376300
H	0.82438900	1.95774600	4.72433700
H	1.71089300	-1.45202400	2.25599700
H	1.50834800	-0.42462900	4.51235400

Amine product of rxn between 22 and a

C	-5.24579400	0.70644300	-2.62821400
C	-3.77819700	0.48344000	-2.62829800
H	-5.79400800	0.50203200	-3.54634300
H	-5.81864800	0.55439400	-1.71596200
H	-3.31519900	0.12543500	-3.54626400
H	-3.27636400	0.16828800	-1.71596300
N	-4.32108200	1.85084100	-2.59569200
H	-4.25783400	2.27037200	-3.52279400

Amine product of rxn between 23 and a

C	-1.15566900	-0.48417500	-0.13820000
C	1.15530600	-0.48501000	-0.13824200
C	0.77740700	1.00295000	0.08619500
C	-0.77666400	1.00353500	0.08610800
N	-0.00046400	-1.29131700	0.28569800
H	1.32619000	-0.67195700	-1.20652300
H	1.19718300	1.65259000	-0.68891000
H	-1.19585600	1.65338300	-0.68913900
H	-1.32677500	-0.67106500	-1.20645500
H	-0.00045400	-1.32537000	1.30605700
H	2.05655900	-0.79383300	0.39982600
H	1.16395900	1.35611100	1.04860400
H	-1.16306400	1.35712600	1.04841900
H	-2.05710700	-0.79230300	0.39995700

Amine product of rxn between 11 and a

C	-8.89307200	-0.87415100	0.68246700

C	-6.83760800	0.46510200	0.66854500
C	-7.80829500	1.47951100	1.25669900
C	-9.43358500	0.33078000	-0.08282300
H	-5.89300600	0.49760700	1.22753500
H	-9.09167700	0.28851600	-1.13002100
H	-9.30877800	-0.85897900	1.69918200
H	-8.02005400	1.21848200	2.30564400
H	-7.36601800	2.48101900	1.24634400
H	-10.53028700	0.33158400	-0.09135700
H	-9.23787100	-1.80147700	0.21317300
H	-6.61343200	0.76802500	-0.36759200
N	-7.42127000	-0.88606900	0.75334200
H	-7.05271500	-1.45323400	-0.00467300
O	-9.02319300	1.56492600	0.51108600

Amine product of rxn between 24 and a

N	-1.06867700	0.07052900	-0.04238800
C	-2.14495200	0.82573600	0.61303900
C	-1.67476200	2.23890900	0.94162700
H	-0.77892100	2.20854200	1.57156000
H	-1.42442500	2.79198200	0.02665400
H	-2.45522800	2.80134000	1.46719700
H	-0.86594500	0.49729900	-0.94651200
H	-1.39260700	-0.87311300	-0.25134500
H	-3.07351400	0.88079800	0.01653100
H	-2.40073700	0.30014300	1.54165600

Amine product of rxn between 25 and a

N	0.07064700	-0.93620800	-0.23871400
C	0.11951400	0.18769700	-1.19165200
H	-0.42268000	-0.12048000	-2.09301800
H	1.16705300	0.33087500	-1.48057600
C	-0.45232600	1.48992400	-0.66637800
C	-1.82178700	1.76514700	-0.77831900
C	0.36315700	2.41278300	0.00156300
C	-2.36491900	2.92884900	-0.23278900
H	-2.46576800	1.06123200	-1.30181100
C	-0.17468700	3.57845900	0.54895400
H	1.42979000	2.21599300	0.08874500
C	-1.54175300	3.83902800	0.43415100
H	-3.42854000	3.12870300	-0.33261900
H	0.47326800	4.28565600	1.06005500
H	-1.96199200	4.74750800	0.85671500
H	-0.89884900	-1.07875100	0.04652000

H	0.55846100	-0.66047200	0.61431000

Amine product of rxn between 26 and a

N	0.35715800	-1.08917100	-0.42883200
H	-0.35009100	-1.82008600	-0.50699000
H	1.22200400	-1.51082000	-0.76766800
C	-0.00813100	0.03268800	-1.29806200
C	-1.30786600	0.71499700	-0.85103500
C	1.00431600	1.18315800	-1.22302800
H	-0.11853700	-0.26256300	-2.35888600
C	-1.26860100	2.10329500	-1.52635700
H	-1.27478800	0.81074700	0.24171500
H	-2.20566700	0.13949600	-1.10550500
C	0.24087000	2.40266300	-1.78171000
H	1.93478600	0.97446800	-1.76409700
H	1.26149100	1.33332900	-0.16672500
H	-1.81830100	2.08670700	-2.47415400
H	-1.74234100	2.86744500	-0.90181400
H	0.42898500	2.51358400	-2.85563300
H	0.56565600	3.33434100	-1.30756400

Amine product of rxn between 27 and a

N	0.53243000	-0.71581900	0.01359700
H	-0.09363000	-1.01185500	-0.72647900
H	0.59178700	-1.41788400	0.74214000
C	0.31267400	0.58427200	0.47606200
C	-0.26650900	1.55497600	-0.35994600
C	0.71971800	0.96489500	1.76675300
C	-0.42933300	2.86472200	0.08572300
H	-0.58407600	1.27379700	-1.36153200
C	0.55251900	2.27760000	2.20208300
H	1.17118100	0.22393200	2.42270100
C	-0.02293200	3.23953800	1.36858800
H	-0.88123800	3.59792900	-0.57752200
H	0.87323500	2.54813300	3.20489900
H	-0.15311800	4.26131800	1.71219900

Amine product of rxn between 28 and a

N	0.36693600	-0.79734900	-0.39895800
C	1.63222500	-0.97322500	0.08852200
O	1.89389100	-1.75350600	0.99274400
O	2.52005200	-0.19605200	-0.57530700
C	3.95858400	-0.27348100	-0.28524000
C	4.47579100	-1.68295400	-0.57638900

H	5.56774100	-1.69918800	-0.48740600
H	4.05533500	-2.40803700	0.12190000
H	4.21319300	-1.98137300	-1.59752700
C	4.22702300	0.15643600	1.15755600
H	5.30778600	0.19955700	1.33294300
H	3.81466000	1.15476500	1.34271800
H	3.78339100	-0.54577700	1.86439100
C	4.54941300	0.73474900	-1.26961600
H	4.14088600	1.73516800	-1.09164200
H	5.63726700	0.77957900	-1.15419300
H	4.32218700	0.44784300	-2.30176100
C	-0.06762100	0.23633700	-1.33213200
H	-0.85311500	-0.18829400	-1.96623000
H	0.78262200	0.46672700	-1.97676600
C	-0.58302000	1.49367600	-0.65625100
C	-1.89023100	1.93654100	-0.87753400
C	0.24366200	2.22713900	0.20635100
C	-2.36755600	3.09273900	-0.25473900
H	-2.54046800	1.37207000	-1.54225000
C	-0.23010900	3.37885700	0.83112800
H	1.26103100	1.89082200	0.38217600
C	-1.53833700	3.81594400	0.60208600
H	-3.38670600	3.42313300	-0.43639200
H	0.42143700	3.93898600	1.49651900
H	-1.90673700	4.71404300	1.09009100
H	-0.34191600	-1.23965000	0.17122800

Amine product of rxn between 29 and a

N	1.48217400	0.41977800	0.20204200
C	1.06794600	1.59131300	0.95184900
H	1.59813700	1.63697900	1.90791900
H	1.28000200	2.51758200	0.40355600
H	-0.00619700	1.52848800	1.14088100
C	2.79635300	0.15446200	-0.03248800
O	3.69463400	0.87255100	0.40003600
C	3.07932100	-1.09284800	-0.85290400
H	3.68023400	-1.78443300	-0.25303800
H	2.17706300	-1.61230300	-1.19153600
H	3.67725500	-0.81514800	-1.72688200
H	0.77514800	-0.17197200	-0.20789500

Amine product of rxn between 30 and a

N	-1.35343500	0.06179000	-0.13260400
C	-1.46264900	-1.27676400	-0.53912000

O	-0.59493300	-1.72689400	-1.26861600
C	-2.64121800	-2.07834800	-0.05443100
H	-2.66009800	-2.12485400	1.03805700
H	-3.58282400	-1.61469200	-0.36159400
H	-2.55836200	-3.08238300	-0.47432100
C	-2.18055900	0.82588900	0.68824900
O	-3.19577400	0.39500100	1.20664700
C	-1.68851600	2.24756400	0.87007800
H	-0.68709400	2.25713200	1.31650400
H	-1.62844400	2.76267500	-0.09599000
H	-2.37974000	2.78304600	1.52216600
H	-0.52979100	0.51467600	-0.51554100

Amine product of rxn between 31 and a

N	0.38071400	-0.84680100	0.67760400
H	0.79744800	-1.34525400	1.45288500
C	0.25916100	0.52061200	0.88991500
C	-0.64280900	1.31537400	0.15760500
C	1.08451200	1.15142300	1.84394300
C	-0.70097400	2.69320700	0.37191600
H	-1.29600600	0.86054600	-0.57900300
C	1.01219300	2.52395800	2.04991800
H	1.78547200	0.54796800	2.41700500
C	0.11920100	3.31151400	1.31509700
H	-1.40501800	3.28588500	-0.20696600
H	1.65908800	2.98355300	2.79298400
H	0.06500100	4.38364700	1.47829200
C	-0.63683600	-1.59331000	-0.03598600
H	-0.37847800	-2.65485800	-0.00091800
H	-1.64812200	-1.46461500	0.38353100
H	-0.66737600	-1.29420700	-1.09011700

Amine product of rxn between 32 and a

N	-0.64264500	0.51931900	-0.03540000
S	-0.61815800	-0.92107600	0.87746800
O	-0.49250900	-0.53487100	2.28356500
O	-1.76921900	-1.68374100	0.38652200
C	0.87962500	-1.75740600	0.39317700
C	0.83301700	-2.70077000	-0.63396800
C	2.09045000	-1.39758700	0.99084200
C	2.01866700	-3.29579200	-1.05863600
H	-0.11814000	-2.97054700	-1.07996400
C	3.26457900	-1.99868800	0.54752400
H	2.10868400	-0.66470400	1.78888800

C	3.24842300	-2.95519900	-0.47849700
H	1.98923000	-4.03837900	-1.85140200
H	4.21004200	-1.72325100	1.00701800
C	4.52456100	-3.62108000	-0.92482500
H	4.79928300	-4.43482200	-0.24065900
H	4.42318200	-4.05302600	-1.92530700
H	5.36096700	-2.91392300	-0.93855700
C	0.47734900	1.40043200	0.03825600
C	1.30866500	1.57546700	-1.07387000
C	0.74028400	2.09092400	1.22709700
C	2.39195000	2.45023900	-1.00198300
H	1.10645900	1.02141000	-1.98695700
C	1.84240000	2.94156300	1.29793300
H	0.08401600	1.95068000	2.07751400
C	2.66758900	3.12920000	0.18627900
H	3.02840900	2.58800000	-1.87135300
H	2.04694100	3.47269000	2.22315700
H	-0.91184700	0.28655400	-0.98990400
H	3.51745000	3.80284000	0.24494500

Amine product of rxn between 33 and a

N	-1.09432800	0.87510500	0.14235000
C	-1.25429100	-0.52511800	0.23331400
C	-0.64497800	-1.19693800	1.30368600
C	-1.95308600	-1.26437000	-0.73067000
C	-0.74762600	-2.58133100	1.41542600
H	-0.10062500	-0.62388100	2.04962900
C	-2.07089400	-2.64783400	-0.59770900
H	-2.38266900	-0.76440700	-1.59051700
C	-1.47051700	-3.31406800	0.47124000
H	-0.26948100	-3.08678500	2.24973000
H	-2.61999900	-3.20754200	-1.34970000
H	-1.55764100	-4.39247400	0.56375000
C	-1.98635800	1.87280000	-0.19864400
O	-1.61771400	3.04159000	-0.18190800
C	-3.40253100	1.48331800	-0.56655400
H	-3.46864100	1.25248000	-1.63602800
H	-3.76387500	0.61517100	-0.00945800
H	-4.04271500	2.34560500	-0.36954500
H	-0.22034200	1.25088000	0.49437200

Amine product of rxn between 37 and a

N	-0.02539400	0.43625500	0.26801100
C	-0.35275900	-0.89468600	0.50547000

O	-1.07991100	1.89690100	-1.61014100
O	0.43704300	-1.75253200	0.84776300
C	-2.54200600	-2.24003300	0.40986300
C	-1.81496800	-1.06311100	0.26811000
C	-2.45796400	0.10851500	-0.13186100
C	-3.81687200	0.16309900	-0.40126300
C	-4.54206600	-1.02491500	-0.25714500
C	-3.91318400	-2.20973400	0.14292900
H	-2.04148800	-3.15076300	0.72114700
H	-4.29917000	1.08428200	-0.71033600
H	-5.60850700	-1.02386000	-0.45935900
H	-4.49992000	-3.11690200	0.24729600
S	-1.30154500	1.46238400	-0.22994200
O	-1.55878700	2.47276600	0.79711000
H	0.92530700	0.78589100	0.33402500

Amine product of rxn between 38 and a

N	-0.18893000	0.56123100	0.17373200
C	-0.62091800	-0.70097700	0.59980900
C	-1.21571200	1.35085500	-0.36015800
O	-1.09278400	2.47734700	-0.79722300
O	0.07780100	-1.56302200	1.09372100
C	-3.02379600	-1.72142400	0.52409000
C	-2.08583600	-0.72286200	0.30947000
C	-2.44110600	0.50257400	-0.26325000
C	-3.74736200	0.77398200	-0.64163200
C	-4.70209000	-0.22908300	-0.42861900
C	-4.34616800	-1.45642400	0.14456300
H	-2.74017200	-2.66991100	0.96874400
H	-4.01491000	1.72730500	-1.08591500
H	-5.73550600	-0.05402500	-0.71243600
H	-5.10901000	-2.21423100	0.29607200
H	0.77307300	0.87019500	0.23807100

Amine product of rxn between 39 and a

N	-1.21043600	0.17775800	0.11754900
C	-1.46124700	-1.07255000	0.67300100
C	-2.26072100	0.72224600	-0.61354800
C	-2.88109700	-1.46177700	0.27216100
C	-3.40855500	-0.28170500	-0.56018900
H	-2.84173600	-2.40158200	-0.28688600
H	-4.27995000	0.20676400	-0.11360100
O	-2.23520000	1.79726300	-1.17483300
O	-0.67468400	-1.70677900	1.34400400

H	-3.68181500	-0.55849900	-1.58277400
H	-3.46412400	-1.64965300	1.17864500
H	-0.32444700	0.65702400	0.23658900

Amine product of rxn between 40 and a

N	-0.99624000	0.00401400	0.29973900
C	-1.52953700	-1.25768700	0.03467000
C	-1.53314100	1.25824100	0.00760100
C	-2.87188100	-1.26491800	-0.66833900
C	-2.87436500	1.24591100	-0.69753600
H	-2.67069100	-1.34386600	-1.74650100
H	-3.39532900	2.16721700	-0.42490300
O	-0.92334000	2.27072800	0.30129800
O	-0.91553800	-2.26182700	0.34768300
H	-2.67162000	1.29914400	-1.77697100
H	-3.39033500	-2.18058700	-0.37285300
C	-3.69428800	-0.00681500	-0.37904800
H	-3.99103400	0.00520200	0.67690200
H	-4.61438700	-0.01457000	-0.97124700
H	-0.09005300	0.01009600	0.76069800
